# Recent progress of emitting long-wavelength carbon dots and their merits for visualization tracking, target delivery and theranostics

**DOI:** 10.7150/thno.80579

**Published:** 2023-05-21

**Authors:** Chao Li, Jiamin Huang, Liwen Yuan, Wenqing Xie, Yupeng Ying, Chengzhe Li, Yahang Yu, Yuanhu Pan, Wei Qu, Haihong Hao, Samah Attia Algharib, Dongmei Chen, Shuyu Xie

**Affiliations:** 1 National Reference Laboratory of Veterinary Drug Residues (HZAU) and MAO Key Laboratory for Detection of Veterinary Drug Residues, Huazhong Agricultural University, Wuhan, Hubei 430070, China.; 2 Key Laboratory of Prevention & Control for African Swine Fever and Other Major Pig Diseases, Ministry of Agriculture and Rural Affairs, Huazhong Agricultural University, Wuhan, Hubei 430070, China.; 3 Department of Clinical Pathology, Faculty of Veterinary Medicine, Benha University, Moshtohor, Toukh 13736, QG, Egypt.

**Keywords:** Carbon dots (CDs), Long wavelength, Visualization tracking, Imaging, Target delivery

## Abstract

As a novel strategy for *in vivo* visualization tracking and monitoring, carbon dots (CDs) emitting long wavelengths (LW, 600-950 nm) have received tremendous attention due to their deep tissue penetration, low photon scattering, satisfactory contrast resolution and high signal-to-background ratios. Although, the mechanism of CDs emitting LW remains controversial and what properties are best for *in vivo* visualization have not been specifically elucidated, it is more conducive to the *in vivo* application of LW-CDs through rational design and ingenious synthesis based on the appreciation of the luminescence mechanism. Therefore, this review analyzes the current tracer technologies applied *in vivo* and their advantages and disadvantages, with emphasis on the physical mechanism of emitting LW fluorescence for* in vivo* imaging. Subsequently, the general properties and merits of LW-CDs for tracking and imaging are summarized. More importantly, the factors affecting the synthesis of LW-CDs and its luminescence mechanism are highlighted. Simultaneously, the application of LW-CDs for disease diagnosis, integration of diagnosis and therapy are summarized. Finally, the bottlenecks and possible future directions of LW-CDs in visualization tracking and imaging *in vivo* are detailly discussed.

## 1. Introduction

Non-invasive visualization tracking and imaging for disease diagnosis as well as reconnoitring the targeted delivery and transport of biotherapeutic agents *in vivo* have been explored as one of the most high-performance tools in the field of biomedicine 1. Over the past several years, this technique has also attracted tremendous attention both in clinical diagnostics and therapy, e.g., pathogen detection 2, diagnosis and therapy of cancer 3, and drug-targeted delivery monitoring 4. *In vivo* visualization tracking and imaging generally require low toxicity, good stability, high absorption coefficient, strong fluorescence strength and visualization within the biotransparency window (650-950 nm) of the tracers. In order to realize the visualization tracer and imaging *in vivo*, various strategies containing magnetic resonance imaging (MRI), nuclear medicine imaging, and optical imaging (e.g. fluorescence, luminescence, Raman and photoacoustics) have been successively proposed 5-8. These tracers and imaging technologies have their own unique advantages. For example, MRI with high resolution is converted by different contrast media such as iron oxides 9, Mn^2+^ ions 10, rare earth chelates 11, and heteronuclear magnetic resonance of ^19^F 12. The imaging reporter genes could be used to visualize single cells in a homogeneous background 13. Nuclear medicine imaging has a high sensitivity and controlled duration time of imaging. The radionuclides with a long half-life, such as ^99m^Tc, ^123^I, ^67^Ga and ^111^In could be considered 14-15, when the tracking and imaging should last for a long time. In contrast, ^18^F, ^11^C, ^13^N and ^15^O are the preferred markers with short half-lives 16-17. Nevertheless, the low spatial resolution 18, possible radiation hazards 19 and potential toxicity of the contrast medium 20 limit the applications of radionuclide imaging and magnetic resonance imaging as tracking techniques *in vivo*. The near-infrared (NIR) fluorescence imaging aroused interest in recent years that can penetrate deeper tissues compared to visible light. The NIR fluorescence emission wavelengths divided into the NIR-I region of 700-900 nm and the NIR-II region of 1000-1700 nm are generally considered to be harmless to the organism and greatly reduce background fluorescence interference. These characteristics confirm the meaningful application prospects of NIR fluorescence as an imaging and therapeutic agent in living organisms. At present, the existing imaging agents are often based on organic dyes, precious metal nanoparticles (NPs), and semiconductor oxides 21-22. However, organic dyes have weak thermal stability and photostability. The precious metal NPs and semiconductor oxides are awfully difficult to excrete from the kidney system, thereby posing the risk of visceral deposits and the potential poisonousness of heavy metal elements 23. Therefore, more effective imaging agent exploration is urgently needed.

Carbon dots (CDs) are generally considered a new kind of carbon zero-dimensional nanomaterials. They have sp^2^ hybridized structure containing carboxyl, hydroxyl and aldehyde groups, which enables them to have better solubility and specific interaction with bio-interface *in vivo*. More importantly, multi-color fluorescent CDs could be constructed by some surface functional groups such as C-O and C=N 24, which also contributed to the synthesis of bright red/NIR CDs. Most CDs have excitation-dependent emission, which is often related to the generation or induction of the band gap and surface defects. Although the fluorescence emission mechanism remains debated 25, CDs emitted fascinating and bright red/NIR fluorescence can be regulated through various strategies such as the changing chemical composition 26, particle size 27 and introducing surface defects 28. Moreover, CDs have some other obvious advantages for imaging *in vivo*, e.g., high quantum yield (QY), excellent biocompatibility, water solubility, resistance to swelling and photobleaching and easy of excretion. For example, pristine CDs (EM: 644 nm) derived from citric acid (CA) pyrolysis had little effect on cell viability at a concentration of 2 μg/mL 29. Nickel-pPCDs prepared by using p-phenylenediamine and nickel ions (Ni^2+^) as raw materials had excellent anti-photobleaching properties. The red fluorescence intensity from Ni-pPCDs remained above 65% of its original fluorescence strength after 1 h continuous laser irradiation at 552 nm 30. The cationic CD (+20 mV) modified with 4-carboxybutyl triphenylphosphonium can emit different fluorescence when combined with double-stranded DNA and single-stranded RNA in living cells, which can be employed for real-time monitoring of DNA and RNA 31. More importantly, these properties of deep tissue penetration, low photon scattering, satisfactory contrast resolution and high signal-to-background ratios possessed by red/NIR CDs make them more suitable for *in vivo* tracking and imaging. Lesani et al. developed two-photon red fluorescence CDs with penetration depths up to 280 μm in porcine skin tissue 32. Hua et al., synthesized the red wavelength-emitting CDs with imaging resolutions up to 80 nm 33. It cannot be hardly found that these features enable CDs as excellent candidates for fluorescent labeling and tracer imaging.

It is well-known that visualization of the target sites is of milestone significance for the diagnosis and treatment of diseases, which allows for therapeutic medicine with more precision and lower side effects. Therefore, a suitable targeted imaging agent in combination with contemporary therapeutic tools may be one of the most promising strategies for precision medicine applications. The research found that CDs combined with photothermal therapy (PTT) and photodynamic therapy (PDT) can effectively kill cancer cells and minimize the interactions of CDs with non-target tissues and only exert therapeutic activity where it is needed. As ordinary cells and body systems are unaffected, there are fewer side effects and faster recovery times 34. The red emissive CDs anchored chlorin e6 (Ce6) (0.56% of mass) possessed superior photothermal (PT) conversion efficiency (46%) and efficient singlet oxygen production under a 671 nm NIR laser 35. The CDs prepared by hypocrella bambusae (HB) can effectively generate heat (27.6%) under 635 nm laser irradiation 36. CDs have higher conversion efficiency and efficient singlet oxygen production due to emitting NIR wavelengths for PTT and PDT therapy. Moreover, the common tracking technology *in vivo* in biomedical engineering is mainly via visual tracking of carrier-loaded fluorescent dye, which may cause false positives in target sites that cannot be accurately quantified due to carrier damage or dye leakage. However, the carrier with itself visual performance can effectively avoid the above possible problems to conduct more accurate quantification because the carrier destruction will lead to fluorescence disappearance. Thus, many CDs are widely reported for targeted drug/gene delivery with simultaneously holding the capacity of imaging-track. The research demonstrated that the targeted drug/gene delivery system simultaneously with imaging-trackable ability is widely reported for CDs. For example, negatively charged red fluorescent CDs prepared by thiophene phenylpropionic acid polymers and loaded with positively charged coptisine exhibited obvious target imaging in cancer cells 37. The long wavelength (LW) emission PEI-CDs synthesized by using 1, 2, and 4-triaminobenzene as the carbon source and polyethyleneimine (PEI) as the surface modifier held effective siRNA delivery with imaging-trackable ability 38.

**Figure 1:**
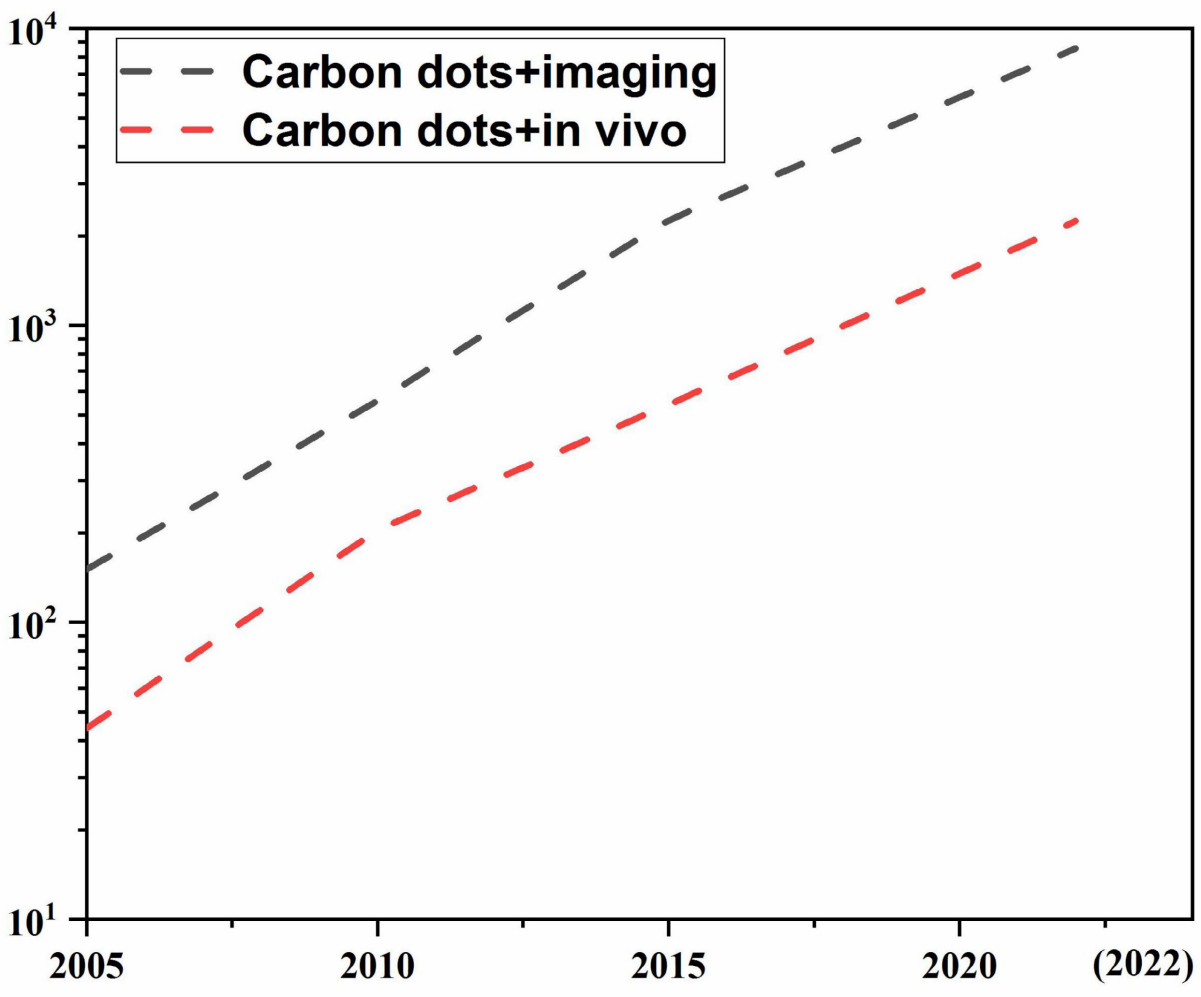
CDs-related publications. The black and red line represents data by searching keywords of “carbon dots, imaging” and “carbon dots, *in vivo*”, respectively. (update to October 31, 2022).

It is not hardly concluded from the above reports that CDs have many advantages applicable to the visualization tracking and imaging *in vivo.* However, the excellent properties (e.g., unique optical properties, fluorescence stability, easy excretion etc.) of CDs for *in vivo* applications and unique features (e.g., deep tissue penetration, low photon scattering, and high contrast resolution and signal-to-background ratios) of emitting LW (LW-CDs: the wavelength range is defined as 600-950nm) as well as the synthesis mechanism to produce these excellent properties have not been systematically summarized. Therefore, we searched related publications about CDs for application *in vivo* in PubMed, Scopus, Web of Science, and Cochrane Central register using relevant keywords (carbon dots and imaging) or (carbon dots and *in vivo*). Figure 1 shows the rapid development of CDs in biomedicine in recent years, especially in the last 5 years, the number of articles published has increased exponentially. However, there are only a few articles about LW-emitting CDs explored for *in vivo* imaging. Based on these limited and related publications, this review first summarizes the development of current tracer technologies and their properties. Secondly, the physical mechanism of emitting LW fluorescence for *in vivo* imaging including reflection, scattering, absorption, and autofluorescence is elaborated. Thirdly, the latest research progress of CDs used as tracers and thier outstanding performances for *in vivo* application are in detail analyzed. Subsequently, the merits of emitting LW-CDs for tracking and imaging are emphasized. Next, the factors affecting their synthesis and luminescence mechanism as well as their application potential in the diagnosis, integration of diagnosis and treatment, and drug/gene target delivery are concisely summarized. Finally, the bottlenecks of emitting LW-CD for visualization tracking and imaging *in vivo* and the possible future directions are proposed. This review will provide effective regulatory strategies to inspire research interest in exploring novel-emitting LW-CDs, thereby promoting their applications in life sciences.

## 2. Current tracking techniques *in vivo*

Tracer imaging is an important diagnostic way of monitoring *in vivo* response and evaluating the outcome of targeted therapy. *In vivo* tracking imaging technology has achieved outstanding progress from 20^th^-century radiography and y-scintigraphy to current radionuclide imaging technology, magnetic resonance imaging, computed tomography and optical imaging 5,7,9,39. Currently, diverse imaging techniques have been explored to address the demand for chemical identification. They commonly use electromagnetic energy to detect biological materials. Bioluminescent agents, traditional fluorescent dyes, isotopes, and nanomaterials have been successfully developed as imaging agents. For example, a small ultra-red fluorescent protein NPs synthesized by An et al. can be used for non-invasive tumor imaging *in vivo* for 7 days 40. Elisa groups evaluated the ability of five different optical and nuclear tracers (including red fluorescent protein, near-red fluorescent dye, radiolabel, luciferase, and radioisotope) to track extracellular vesicles (EV). All five tracers showed bright imaging *in vivo*, among which radioactivity was the most accurate tracking method for EV 41.

However, some disadvantages of these traditional imaging agents limit their clinical application. For example, the most frequently used fluorescent dyes (coumarins, rhodamines and anthocyanins) and proteins are prone to photobleaching and susceptible to being interfered with *in vivo* background fluorescence 42. Luciferase can be used as a marker for a variety of enzyme genes due to its specificity and sensitivity, unfortunately, it requires an exogenous substrate to emit light 43. The emerging nanomaterials are difficult in large-scale preparation, especially for the long afterglow NPs 44. The semiconductor quantum dots have certain cytotoxicity 45. The upconversion NPs have low luminous efficiency 46. The magnetic NPs are prone to agglomeration 47. In short, these materials have certain deficiencies in their tracking and imaging applications *in vivo*. The characteristics, advantages, and disadvantages of various imaging agents are systematically summarized in Table 1. It is not hardly concluded that imaging agents with good biocompatibility, photostability, strong fluorescence, and visualization in the bio-transparent window need to be further explored.

## 3. Physical mechanism of emitting LW fluorescence for *in vivo* imaging

**Figure 2:**
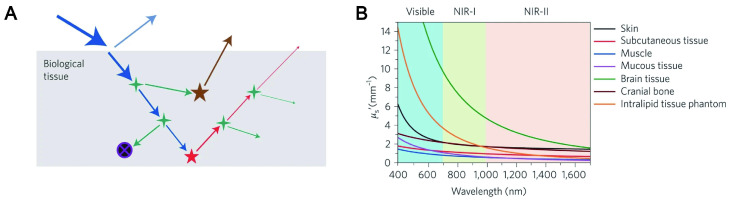
Light-tissue interactions. (A) Excitation light (blue), interface reflection (cyan), scattering (green), absorption (black circle with purple cross) and autofluorescence (brown) all contribute to the loss of signal (fluorescence, red) and the gain of noise. (B) Reduced scattering coefficients of different biological tissues as a function of wavelength in the 400-1700 nm region. Adapted with permission from 65. Copyright 2019, Springer Nature.

NIR fluorescence imaging offers unparalleled advantages over visible spectroscopy due to the physical mechanism of photon-tissue interaction. In order to obtain satisfactory imaging, it is necessary to preserve each photon as much as possible, and the excitation photon needs to reach a specific depth to excite the three-dimensional distribution of fluorophore molecules in the biological tissues. Simultaneously, the emitted photons can avoid tissue interference and are collected by the photodetector. The four processes related to excitation photons and emission photons of penetrating biological tissue are analyzed (Figure 2A-B) 65-66.

### 3.1. Reflection

Reflectance refers to the phenomenon that light changes the direction of propagation and returns to the original matter when it travels to a different matter. Owing to the difference in refractive index between the medium and the surface tissue of the object, a small proportion of the excitation light is reflected at the interface. Reflections generally do not cause a severe signal loss because the curvature and roughness of the surface are less than the imaging feature, and the excitation irradiance is near normal. The actual problem is the reflection process of the emitted light. Due to the rough surface of the interface, random reflections of the emitted light generate a lot of background noise. To address this problem, the refractive-index matched medium has been used in practical imaging processes, especially for the application of super-resolution techniques. Furthermore, based on the Fresnel equation, the reflection process depends on the angle of incidence and the difference in refractive index, which is independent of wavelength since the index of refraction varies very little with wavelength. As a result, the dramatic advances in NIR imaging across the visible spectrum cannot be responded in the reflection process.

### 3.2. Scattering

Scattering is a phenomenon that the secondary radiation wave is distributed when the surface curvature is large or even not smooth. Scattering events will occur when excitation and emission photons encounter experimental tissue composed of homogeneous matter. Relevant studies have shown that almost all biological tissues are inversely proportional to wavelength, and the wavelength-dependent index varies with the tissue models and different tissues. In addition, the scattering coefficient is largely wavelength-dependent with an inversely proportional relationship. The reduction in dispersion coefficient is accompanied by a reduction in background noise, which means that LW can significantly reduce the interference of background noise. In the NIR region, both excited and emitted photons can propagate into deep tissue with an improved signal-to-background ratio. Zhang et al. synthesized pure red luminescent CD (EM: 678 nm) used by formic acid, CA and urea. A bright two-photon red FL signal with negligible photon scattering effect can be clearly observed in the vasculature of the mouse ear 40 min after injection 67. The study also found that CDs emitting at 745 nm had a high signal-to-noise ratio (S/N) of up to 18, which exhibited deeptissue penetration and low photon scattering 68.

### 3.3. Absorption

The absorption of light refers to the phenomenon that the energy of the absorbed photons jumps from a low energy state to a high-energy state under light. Biological tissues consist of many heterogeneous biomolecules that convert photons into heat. In some special organizations, the heat conversion effect is remarkable. This effect will reduce photon generation efficiency and create additional problems, such as thermal reactions. Since absorbance is wavelength-dependent in various tissues, the currently available solution is to empirically measure the substance under study 69. An effective strategy is to use liquid water, which contributes significantly to the elimination spectrum. In the NIR region, workers usually set appropriate optical window boundaries for deep tissue imaging to handle the absorption peaks of liquid water.

### 3.4. Autofluorescence

Autofluorescence refers to the spontaneous fluorescence generated when the external excitation light irradiated tissues, cells and living substances. Tissue autofluorescence is the last significant obstacle that can limit depth imaging. Fluorescence photons from different sources including tissue autofluorescence photons and object fluorescence photons tend to diffuse together and create horrific background noise. Fortunately, relevant studies have revealed that LW fluorescence molecules can gradually reduce the level of tissue autofluorescence. It was confirmed that an autofluorescence-free window appeared when the NIR-II channel was extended to rodent tissue. It is worth noting that the autofluorescence-free window will facilitate *in vivo* fluorescence imaging with minimal background interference of tissue origin, especially when imaging organs rich in endogenous fluorophores such as the liver. It was found that CDs synthesized by using CA and urea with an emission wavelength of 650 nm can penetrate about 1 cm thick tissue and were hardly disturbed by background autofluorescence 70. Similarly, NIR-II CDs synthesized using watermelon juice have been shown to be effective probes for *in vivo* bioimaging due to their lower tissue background fluorescence interference 71.

## 4. General properties of LW-CDs for* in vivo* imaging

**Figure 3:**
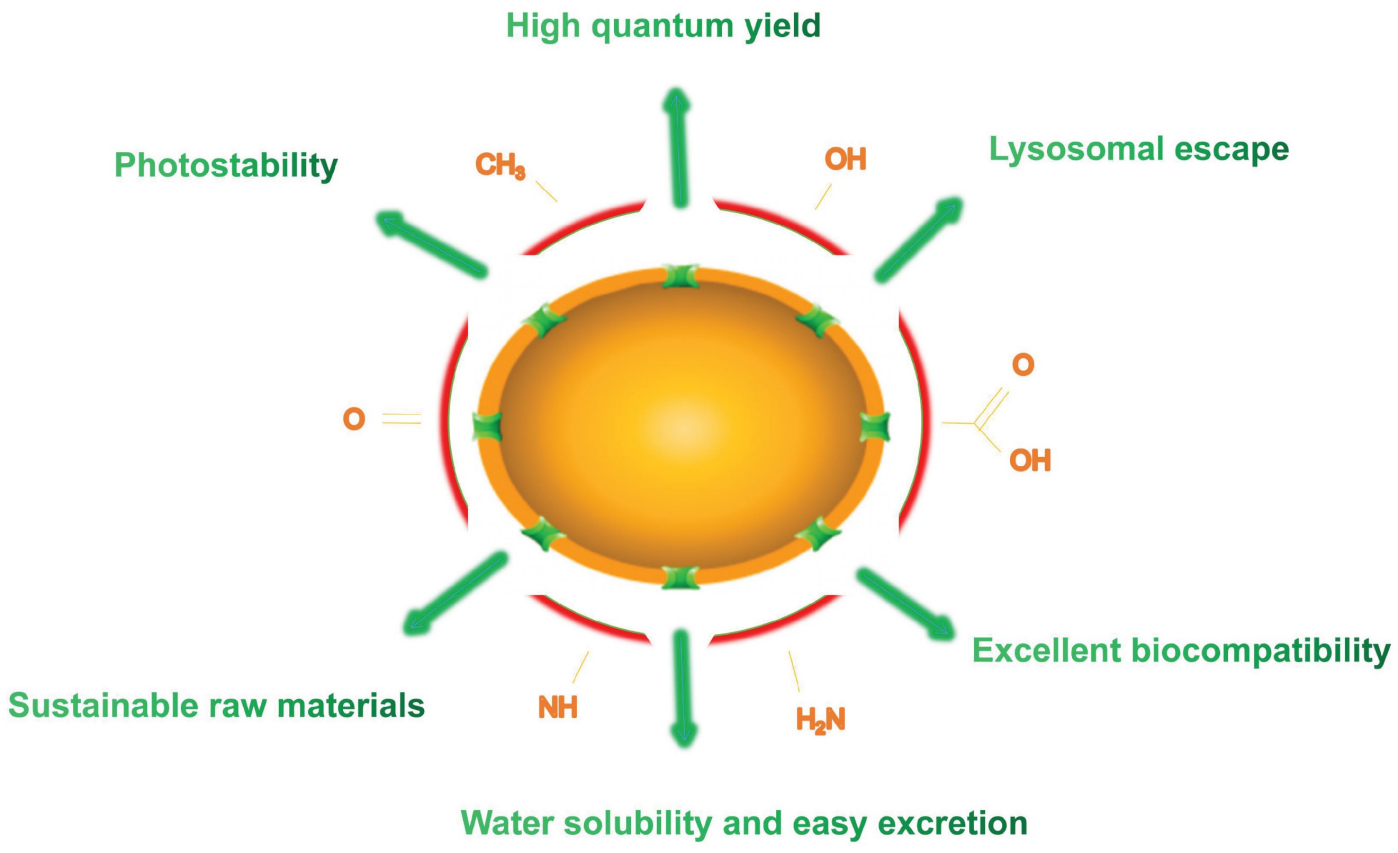
Schematics of emitting LW- CDs.

In the past few years, an extensive study has focused on the properties and applications of CDs composed of carbon that it is a rich, non-toxic and major component of organisms. This structure lays the foundation for their long-term tracking* in vivo*. Compared to traditional fluorescent dyes and fluorescent NPs, CDs hold excellent properties in high QY, photostability, excellent biocompatibility, sustainable raw materials, easy excretion and lysosomal escape (Figure 3). In this section, we summarize and elaborate on the general properties of conventional CDs and LW-CDs to better understand the properties of CDs applied *in vivo*. Since the characteristics of biocompatibility, sustainable raw materials, easy excretion and lysosomal escape of LW-CDs are not essentially different from CDs emitting visible light, these properties are not analyzed separately for LW-CDs in this section.

### 4.1. High QY

**Figure 4:**
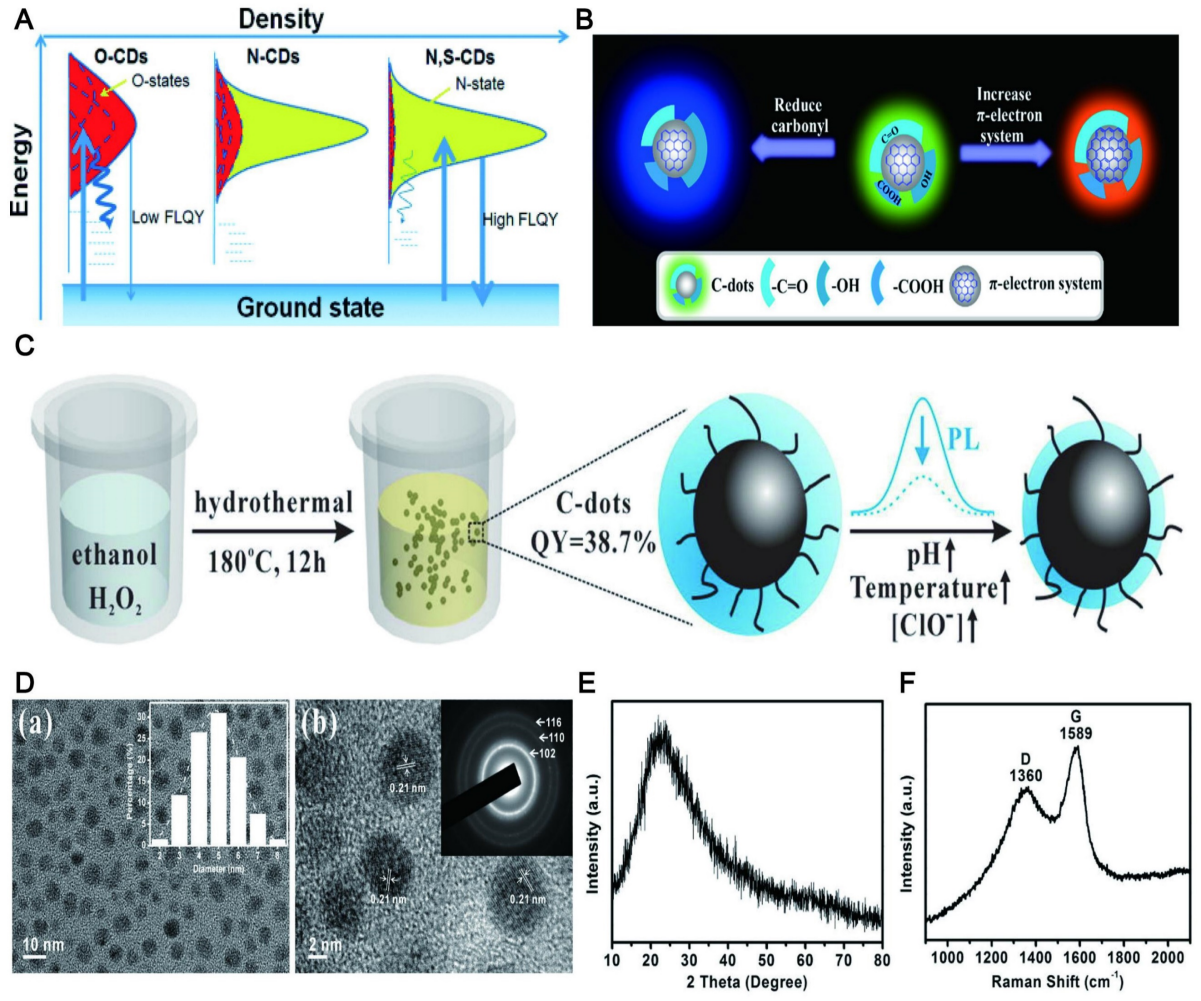
The high QY of CDs. (A) Electrons are excited from the ground state and trapped by the surface states and excited electrons return to the ground state. Adapted with permission from 76. Copyright 2013, John Wiley and Sons. (B) The QY of CDs was affected by the π-electron system and the O-H group. Adapted with permission from 88. Copyright 2018, American Chemical Society. (C) A synthetic strategy for highly QY CDs by hydrothermal treatment of ethanol in aqueous hydrogen peroxide (H2O2) solution. (D-F) TEM image, XRD pattern and Raman spectrum of the CDs, indicating high crystallinity of the CDs. Adapted with permission from 76. Copyright 2015, Elsevier.

QY refers to the utilization rate of light quantum in photochemical reactions. QY is one of the most important merits of CD. The high QY is necessary for fluorescent CDs with LW emission to realize their application *in vivo*. In the early stage, the QY of CDs is often below 15%, which severely limits their application. Significant progress has been made in increasing QY through surface passivation and doping 72,73. Relevant studies 74 confirmed that nitrogen (N) or N/sulfur (N/S) doping led to a high QY of CDs with a yield of up to 78%. The QY of N-doped CDs was dramatically improved when the N entered the framework via intramolecular dehydrolysis. The mechanism of high QY was derived from the effective binding of N to various functional groups (CO, NH, CN, COOH and COC) 75. The functional groups of the CDs could reinforce the conjugation degree of the conjugated system, increase the probability of electron transition from the ground state to the lowest excited singlet state, and eventually improve the QY of CDs (Figure 4A) 76. Similar heteroatom doping includes phosphorus (P), boron (B), selenium (Se), silicon (Si), chromium (Cr), chlorine (Cl), zinc (Zn) and fluorine (F) 77-83. For example, the QY of CDs synthesized by using CA and thiourea was only 11.4%, while the QY of the CDs doped by B can reach 25% 84. The QY of N/P co-doped CDs was as high as 43.2%, while the QY of CDs alone doped by N was only 29.0% 77. This phenomenon may be contributed to the modulation of the chemical and electronic properties of CDs induced by N/P co-doping. The QY of Se/N co-doped CDs could reach up to 52% 85 due to the low electronegativity of Se. The QY of the CDs co-doped by P and Cl was 2 times higher than the single doping 82.

Notably, the QY of the doped CDs may be further improved through strong hydrogen bonding. The QY of the resultant N-doped CDs dispersed in ethanol was increased to 50.3% relative to those dispersed in water (13.5%) 86. This possibly resulted from C=O, O-H and N-H of N-doped CDs surfaces can form strong hydrogen bonds with ethanol molecules, thereby stabilizing the excited states for more efficient radiative recombination. Similarly, the QY of N-doped CDs dispersed in polyvinyl alcohol (PVA) solution was increased to 47% 87. Recently studies proposed that the coupling of the π-electron system to the O-H group decides the size of the energy gap and the QY 88. For example, its QYs were increased by 6-fold when the O-H content was decreased from 16.3% to 10.6% for CDs-NaBH_4_-NaOH. This might be because the O-H group is the main factor to increase the composite ratio of non-radiation to radiation (Figure 4B).

Another meaningful way for improving the QY of CDs is via preparing them into the high crystalline state that can avoid the complex post-treatment of surface passivation and heteroatom doping that requires special N-containing organic precursors. The synthesis method did not need any doping and modification by using catechol as a carbon source 89, and the prepared CDs did not require any post-treatment. The QY of the CDs can reach 32% (Figure 4C), while the QY of high crystalline CDs obtained by the hydrothermal method of ethanol with hydrogen peroxide without further passivation or heteroatom doping dramatically increased to 38.7% 90. The high QY may be due to the highly splendid crystalline nature, which reduced the nonradiative electron-hole recombination center and thus resulted in strong fluorescence (Figure 4D, E, and F).

In addition, CA, folic acid (FA), urea, and thiourea have also been reported to prepare CDs with high QY. The abundant heteroatom of these natural products facilitates the synthesis of heteroatom-doped CDs, thereby eliminating the demand for additional sources of heteroatom. For example, Ding 91 et al. produced red luminescent CDs with QY as high as 53% by heating a formamide solution mixed with CA and ethylenediamine. It was also reported that highly luminescent CDs synthesized by employing FA as a single precursor displayed a high QY of 94.5% in water 92. The commonly used heteroatom-containing precursors include ethylenediamine 93, L-cysteine 94, PEI 95, β-alanine 96, 1,2,4-triaminobenzene 97, diethylenetriamine 98, ethanolamine 99, and ammonium citrate 100. These heteroatom-containing materials improve the QY of CDs with varying degrees since they are affected by the level of heteroatom content. In summary, the QY of CDs is affected by doping, solvent, high crystalline state and heteroatom (summarized in Table 2).

Compared to most above-mentioned UV/blue light-triggered CDs that exhibit visible light emission, the CDs with NIR emission (especially NIR-II) in the range of 650-1700 nm generally face lower QYs. For example, the CD developed by Li with 900-1200 nm emission wavelength has a low QY (0.4%) 101. The highly efficient red CDs synthesized by surface oxidation of large-sized CDs only showed 1.8% QY 102. Therefore, it is very necessary to summarize and develop LW-CDs with high QYs. It is found that the QY of LW-CDs can be significantly enhanced by avoiding the presence of oxygen-containing functional groups during the synthesis. Yuan et al. presented that the QY of LW-CDs was enhanced by 42% by avoiding the presence of carboxyl groups, and the narrow emission spectrum of red-emitting CDs was successfully narrowed down to 30 nm 103. Due to the lack of these oxygen-containing functional groups, the poor water solubility of LW-CDs and the inconvenience of *in vivo* application may also be caused. Some researchers have proposed that the use of highly conjugated aromatic amine molecules (tris(4-aminophenyl)amine, TAPA) and the introduction of oxidative radical reagents can balance the high QY and ideal water solubility of CDs. The QY of the as-synthesized red narrow-emitting CDs (FWHM = 27 ± 1 nm) was up to 84% 104. In addition, the enhanced QY strategies of conventional CDs described above also apply equally well to LW-CDs, e.g., dispersive solvent effects. Liu et al. found that the QY of LW-CDs synthesized by using p-phenylenediamine or o-phenylenediamine as C and N sources was as high as 52.4% with the increase of solvent polarity 105. Additionally, the red-emitting CDs synthesized by using different precursors p-phenylenediamine and isocyanatopropyltriethoxysilane hold a high QY of 99% 106.

### 4.2. Photostability

The excellent photostability of CDs is contributed to their long-term imaging *in vivo*, while long emission wavelengths often have weak fluorescence intensity and possible photo instability. At present, rare research has been implemented on the photostability of CDs, particularly CDs emitted by LW. Compared with commercially available photoluminescent dyes 107, CDs have higher stability and are very resistant to photobleaching. The photostability of CDs is commonly determined by evaluating whether the fluorescence intensity decays 108.

**Figure 5:**
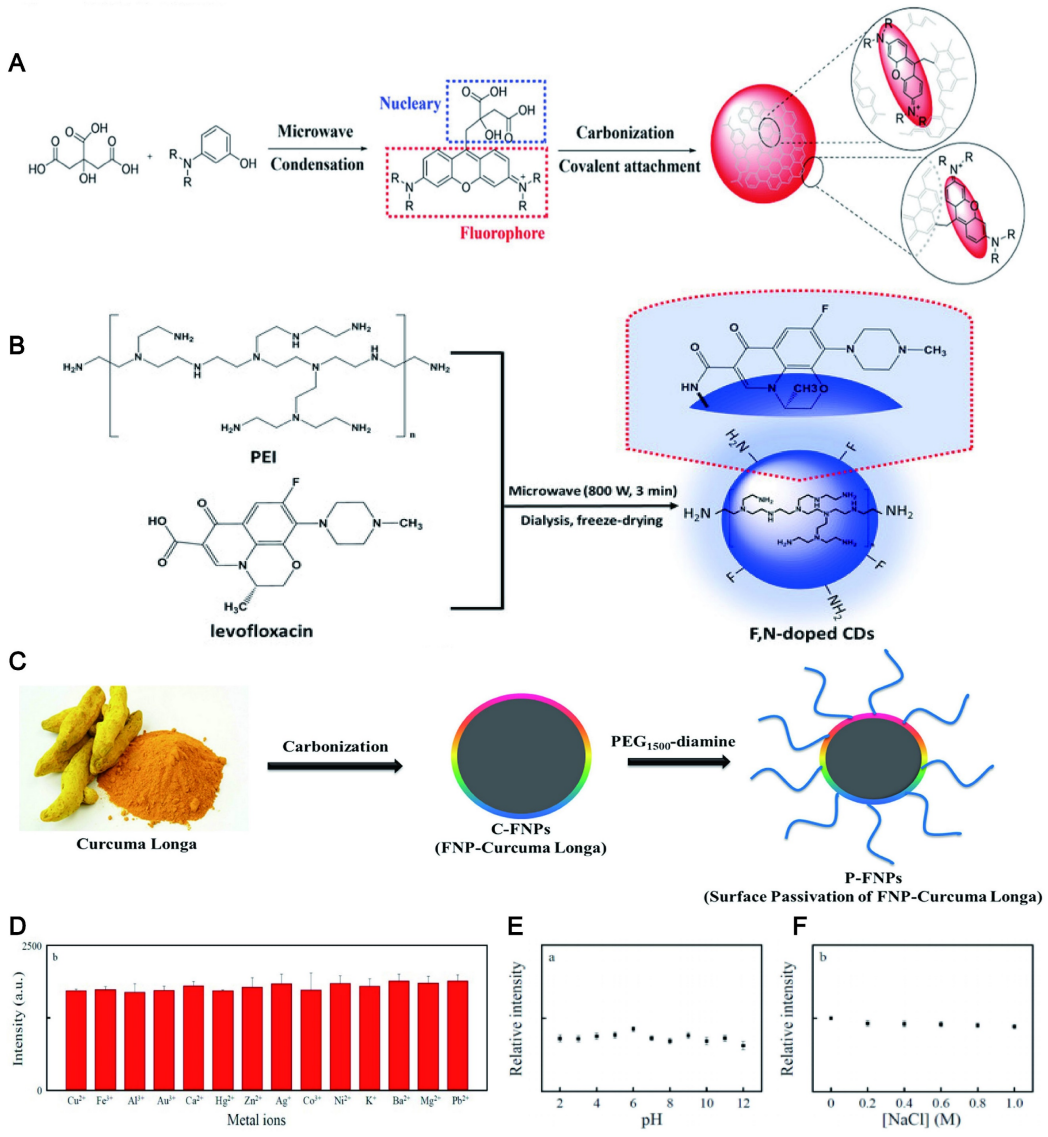
The fluorescence stability of CDs. (A) Synthesis of CDs emitting LWs and being rich in hydrophilic groups. Adapted with permission from 111. Copyright 2019, John Wiley and Sons. (B) Synthesis of F, N Co-Doped CDs. Adapted with permission from 114. Copyright 2018, John Wiley and Sons. (C) The synthesis of carbonized Curcuma longa (C-FNPs) and surface passivation with PEG. Optical stability research of the BN-CDs under (D) various pH value, (E) 365 nm UV irradiation time and (F) ionic strength. At the excitation/emission wavelengths of 520/616 nm. Adapted with permission from 122. Copyright 2019, Springer Nature.

The ideal photostability may be ascribed to that a large number of hydrophilic groups on the surface of CDs can improve their stability in an aqueous solution, thereby enhancing the fluorescence stability 109. Similarly, the fluorescence intensity of the CDs rich in the carboxyl group and hydroxyl group with 3-aminobenzoic acid exhibited overall stability by laser irradiation from 7 to 180 min 110. Recently, the pH-induced hydrophilic CDs synthesized by using CA and m-aminophenol as precursors possessed high photostability and tunable LW fluorescence (Figure 5A). What was even more gratifying was that the fluorescence intensity of CDs was kept almost constant when stored in the refrigerator for approximately 100 days 111. N-CDs with high hydrophilicity commonly exhibit excellent photostability. Moreover, further doping of CDs based on hydrophilicity may promote fluorescence stability. A recent report proposed that N-CDs and N, P-CDs were stable in solution at least for 6 d under laboratory lighting. To evaluate the impact of P-doping on photostability, the accelerated photobleaching study was conducted. The result displayed that the photostability of N-CDs was significantly reduced by 25% compared to the N, P-CDs after 60 min exposure 112. It can be found that more photostability was obtained by doping the N-CDs with the P element. The researcher reported that F with maximum electronegativity could enhance luminescence stability by forming strong hydrogen bonds 113. F, N-CDs can keep their stability at room temperature for about 90 days using levofloxacin and PEI as F and N sources (Figure 5B) 114. The B, N-CDs 115, S, N-CDs 116, N, Cl-CDs 117, P, Cl-CDs 118 and others also have been confirmed to have excellent light stability after doping. In addition, the fluorescence properties of CDs combined with superparamagnetic iron oxide NPs (SPIONs) can be developed to obtain long-lasting fluorescence and excellent magnetic resonance (MR) imaging capabilities 117.

Passivation also can improve the stability of CDs, because their high reactivity and vulnerable surface groups are shielded under the polymer layer such as poly(ethylene glycol) (PEG) 119, PEI 120, PVA 121, polyvinyl pyrrolidone (PVP), etc 122. For example, covering amine groups in curcuma longa-NPs (C-NPs) by using PEG engendered a longer fluorescence lifetime than C-NPs (Figure 5C) 119. Similarly, the fluorescence intensity of the CDs via carbonizing PVP and L-Cysteine was extraordinarily stable even in high ionic strength (1.0 M NaCl) and various pH conditions (2.0-12.0) (Figure 5D, E and F) 122.

Besides, it is discovered that the synthetic route can affect the light stability of CDs. Specifically, even under a strong light source, the CD prepared by the "top-down" method (e.g., by using acid to peel carbon nitride) has extremely high light stability 123. In contrast, CDs prepared by using "bottom-up" methods (usually by hydrothermal or microwave methods) are relatively susceptible to photobleaching due to a large number of surface defects 124.

In the past several years, the literature reports on the fluorescence stability of LW-CDs gradually increased. Recent studies have found that embedding LW-CDs into a rigid matrix Y(OH)_x_F_3-x_ can significantly improve their photostability, thermal stability, chemical stability and temporal stability 125. Similarly, embedding LW-CDs into the lipid bilayer of giant unilamellar vesicles can recover within 5 s after photobleaching and maintain photostability 126. In order to probe the relevant mechanisms of fluorescence stability, Terracina et al. investigated the photostability of LW-CDs by *in situ* observing their time-resolved fluorescence degradation or light absorption changes under equivalent experimental conditions and laser irradiation. It was found that the interaction between the fluorescent unit and the carbonaceous CD core not only affects the excited state decay properties of the former, but also has a protective effect on photobleaching 127. In addition, the study found that the chemically inert emissive aromatics as the center can prevent the photochemical reaction and structural damage of the LW-CDs. Wu et al. synthesized triglyceride-converted CD by pyrolysis, and the amphiphilic CD underwent self-assembly to form a liposome-like structure with a structural orientation similar to that of phospholipids. The fluorescence intensity of the CDs remained unchanged after continuous UV irradiation for 10 min. As a control, Hoechst 33,342 showed a 50% decrease in fluorescence intensity after 1 min of UV exposure 128.

In summary, the photostability of CDs (including LW-CDs) can be improved by a series of strategies including enhancing the hydrophilicity, doping with heteroatom on the surface of CDs, and passivating by using certain high molecular polymers to mask highly reactive groups on its surface (summarized in Table 3). Thus, the photostability can be adjusted by the emission state of the surface to meet the requirement for *in vivo* visualization track.

### 4.3. Excellent biocompatibility

**Figure 6:**
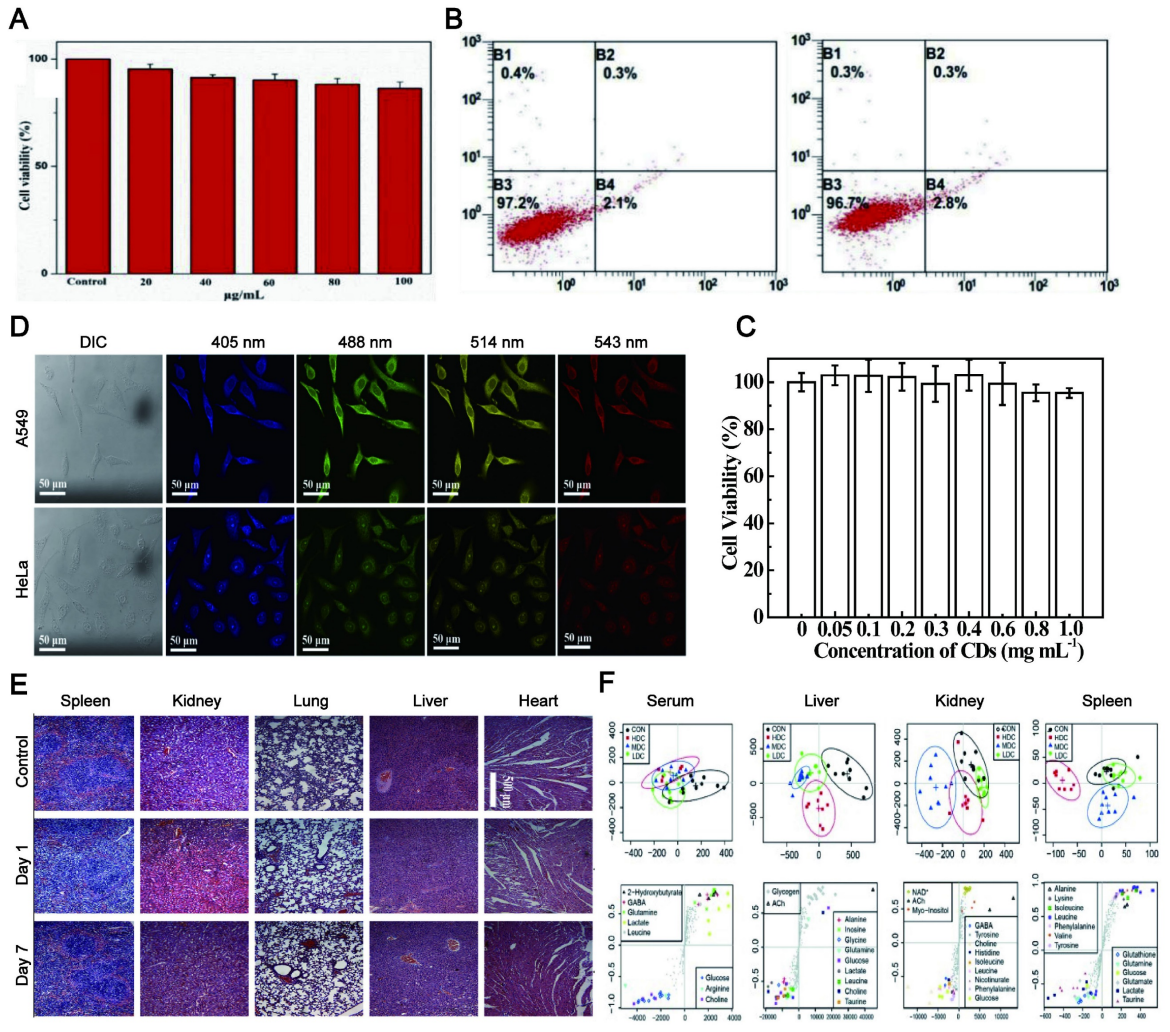
The excellent biocompatibility of CDs. (A) The proliferation rate of HeLa cells incubated in CDs over 48   h with different concentrations of the fluorescent CDs. Adapted with permission from 131. Copyright 2018, Elsevier. (B) Flow cytometry analysis of HeLa cells after the incubation with 100 μg/mL of CDs for 24 h. Adapted with permission from 132. Copyright 2020, Elsevier. (C) Cell viabilities after incubation with different concentrations of CDs for 24 h. (D-E) Fluorescence images of A549 and HeLa cells treated with CDs and H&E stained tissue slices (Heart, liver, lung, kidney and spleen) of mice injected with CNDs aqueous suspension. Adapted with permission from 134. Copyright 2020, Elsevier. (F) OSC-PLS-DA analysis of the NMR data from serum and tissue extracts of the control and C-dot treated groups. Adapted with permission from 135. Copyright 2018, Oxford University Press.

Biocompatibility, including tissue compatibility and blood compatibility, is the prerequisite for *in vivo* tracking imaging. The CDs exhibit excellent biocompatibility and good water solubility. Since the synthesis of CDs is based on carbon-based materials, the biocompatibility of LW-CDs is not significantly different from those emitting visible light. Therefore, the biocompatibility of emitting visible light and LW-CDs will not be analyzed separately here. In most studies, the cytotoxicity of CDs is significantly lower than graphene oxide nanomaterials and QDs 129. The research described that *Puerariae lobatae Radix*-CDs were nontoxic to LO2 cells and RAW264.7 cells at a dose below 250 μg/mL 130 and for Hela cells below 100 μg/mL (48h) (Figure 6A) 131. The viability of Hela cells was merely declined by about 5% even when it was incubated with a relatively high concentration (1.0 mg/mL) of CDs for 24h (Figure 6B-C) 132. The cytotoxicity of CDs is far lower than that of semiconductor quantum dots which can cause neutrophil cell death and platelet aggregation 133. In mice, no pathological damage was observed in various organs (including the heart, liver, lung, kidney, and spleen) after the injection of CDs (Figure 6D-E) 134. Last, *in vivo* toxicology of CDs with different concentrations studied by ^1^HNMR-based metabolomics revealed that CDs did not cause any noticeable tissue damage and biochemical parameter alteration (Figure 6F) 135. The fluorescent CDs synthesized by α-Cyclodextrin (0.43 g) and KH_2_PO_4_ showed almost no adverse effect on blood components when the concentration was below 0.1 mg/mL. The CDs did not impair the coagulation function after intravenous administration at a dose not exceeding 50 mg/kg 136. In addition to emitting LW-CDs, studies have depicted that other colors (blue 137, green 138, yellow 139, and orange 140) of photoluminescence (PL) CDs also exhibit excellent biocompatibility and low toxicity.

Although CDs are less toxic than other imaging agents, such as semiconductor quantum dots and graphene oxide nanomaterials, some of them exhibit dose-dependent growth inhibition on the cells when the exposure concentration is too high. Here “CDs” include carbon nanodots, carbon quantum dots and carbonized polymer dots, because graphene quantum dots (although they also belong to the big family of CDs) are usually more toxic than most of CDs as reported in literature. For example, the cell proliferation was reduced by 60 ± 5% when it was exposed to 25 mg/mL concentrations of CA-CDs, while the cell growth inhibition was not observed when the concentration of CA-CDs was lower than and equal to 7.7 mg/mL 141. Besides, the toxicity of CDs strongly depends on the fabrication materials. For example, the citrate-derived CDs did not produce any distinct impact at a concentration of 2 mg/mL, while the cinnamon-CDs, chilli-CDs and turmeric-CDs brought about 35%, 50% and 50% reduction in the activity of HK-2 at a concentration of 2 mg/mL, respectively 142. The cytotoxicity difference of CDs derived from materials might be ascribed to the functional group discrepancy on the surface of the CDs 143. Their toxicity order was: no doped CDs< N doped CDs< N, S doped CDs 144. That was consistent with previous reports that different carbon nanomaterials exhibited different toxic effects 145. At present, the materials commonly used in the preparation of CDs are less toxic to cells or organisms. They include grass 146, egg 147, soya milk 148, algal blooms 149, beer 150, potato 151, instant coffee 152, sucrose 153, CA 154, banana juice 155, amino acids 156, bombyx mori silk 157, glycerol 158 and N-acetyl cysteine 159. These materials are present everywhere in life with good biocompatibility. Therefore, there are few reports about the toxicity mechanism of CDs. Recently, it is reported that the toxic effect of ginger CDs is related to increased ROS production 160.

The current data above provide valuable safety information for the clinical application of fluorescent CDs. Compared with traditional dyes, semiconductor materials and even carbon-based QDs, CDs have more potential promising for *in vivo* tracing due to their relative safety. For example, graphene QDs exhibited obvious cytotoxicity at very low concentrations (IC50 = 1.5 µg/mL), which is not suitable for *in vivo* tracing 161. The further understanding of its potential toxicity and related toxicity mechanism can better promote its clinical application.

### 4.4. Sustainable raw materials

**Figure 7:**
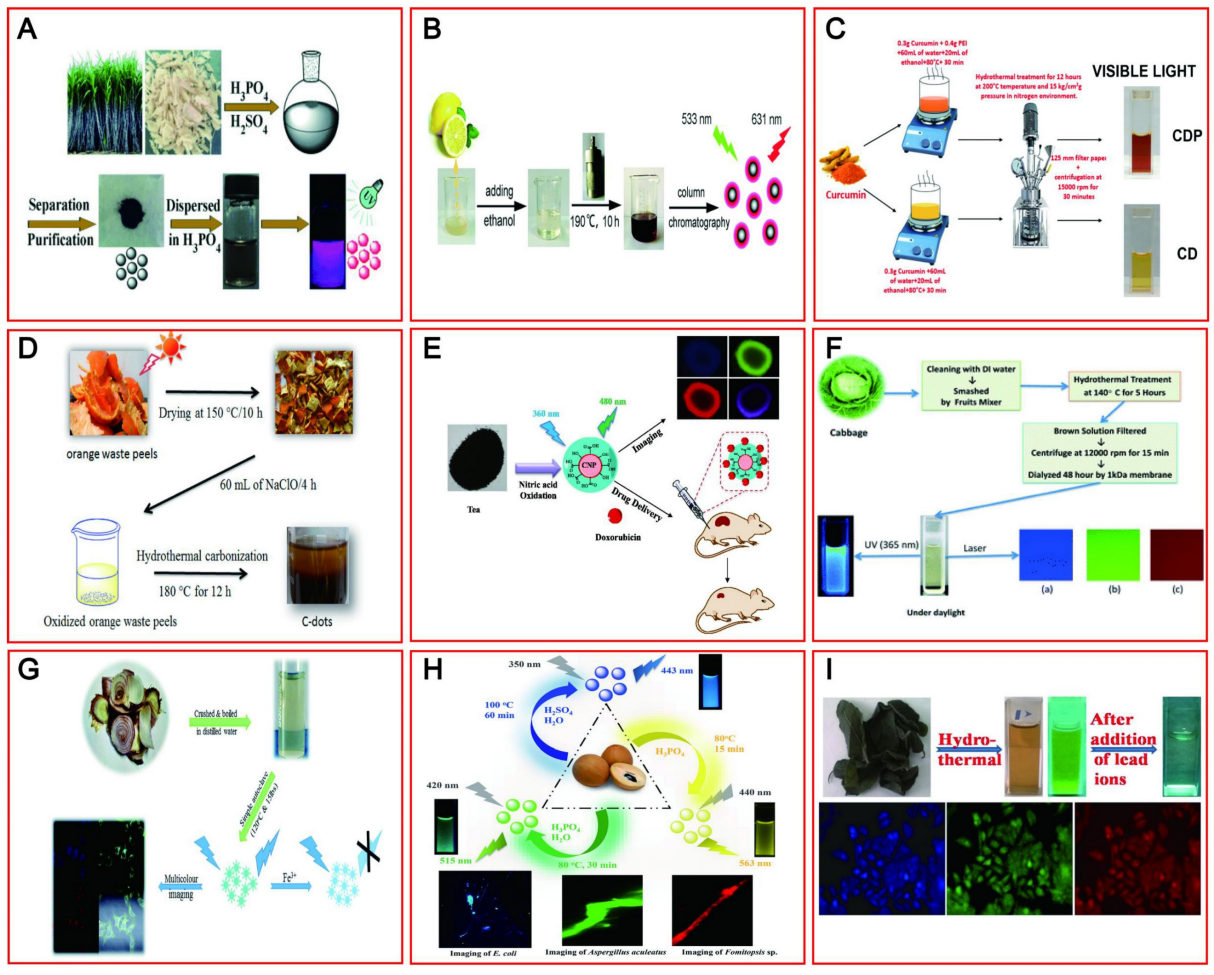
The natural raw materials for synthesizing CDs. (A-I) A process for synthesizing CDs emitting LWs from various sustainable natural raw materials such as sugar cane bagasse, lemon juice, curcumin, orange waste peels, tea, cabbage, onion waste, ocimum sanctum and manilkara zapota fruits. Adapted with permission from [162, 164, 165, 166, 167, 169, 170, 171 and 172]. Copyright 2015, 2012, 2018, 2013, 2017, 2019, 2017, 2015 and 2016, John Wiley and Sons, Royal Society of Chemistry, American Chemical Society, American Chemical Society, Elsevier, Elsevier, Elsevier, Royal Society of Chemistry and Royal Society of Chemistry.

The initial synthesis of CDs is confirmed by the use of carbonaceous materials. However, based on environmental protection and biocompatibility, green renewable CDs (including LW-CDs) have gradually become more popular relative to chemically derived CDs. To achieve cost-effective, simple, and environmentally benign synthetic methods, various green precursors including bagasse 162, Carica papaya juice 163, lemon juice 164, pomelo peels 165, curcumin 166, orange waste peel 167, and black tea 168 have been widely investigated as raw materials to prepare satisfactory CDs with outstanding optical and electronic properties (Figure 7A-I). The pollen, cabbage, apple, and allium fistulosum-derived CDs with emitting blue, green, and red light at different excitation wavelengths have shown promising fluorescent bioimaging applications in colon cell lines (LoVo), human keratinocyte cancer cells (HaCaT), fungal and bacteria cells as well as breast cancer cell lines 169,170. Similarly, onion waste 171, osmium sanctum 172 and manilkara zapota 173 have been used as raw material to prepare the CDs by emitting red fluorescent (summarized in Table 4). Many green and environmentally friendly materials have also been used to synthesize CDs with emitting LW *in vivo*. Importantly, these materials could be continuously regenerated from nature. The sustainable and pollution-free of raw materials will promote more green CDs development and provide the guarantee for the widespread application of CDs in medical fields.

### 4.5. Easy excretion

As a promising *in vivo* tracer, the rapid clearance from the body should be taken into account to ensure their biosafety. The existing tracers including organic dyes, precious metal NPs and semiconductor oxides have been confirmed by most studies to be harmful to humans, in part due to their hard excretion 174. CD and its metabolites do not cause harm to the body due to their easy excretion in different ways. Urine and feces elimination are the main excretion routes (summarized in Table 5).

**Figure 8:**
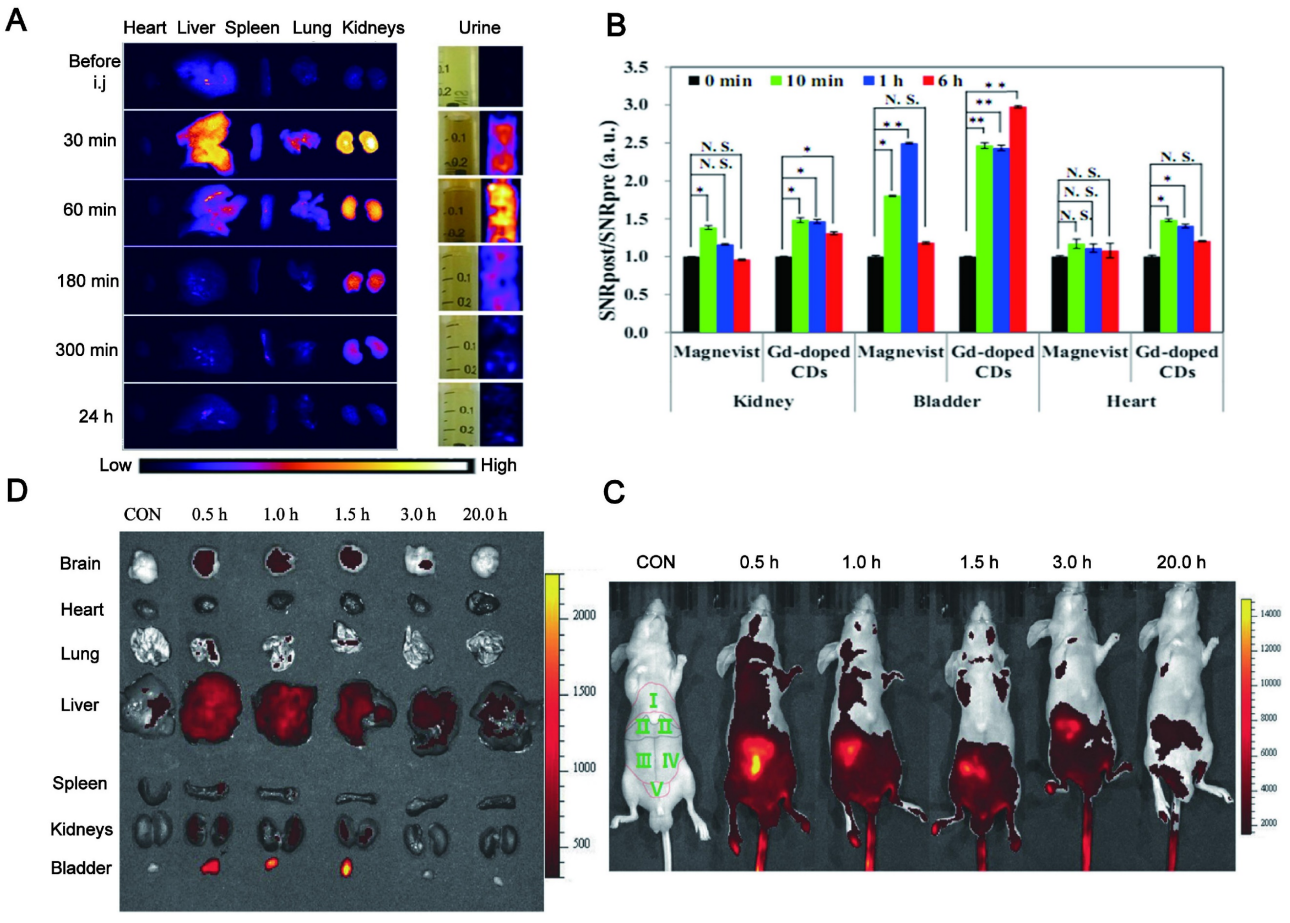
Metabolic pathways of CDs include the liver and kidney. (A) NIR fluorescence images of dissected major organs from mice. Adapted with permission from 175. Copyright 2017, Springer Nature. (B) Quantification of signal changes (SNR ratio) in kidney, bladder, and liver at different time points after administration (n = 3). Adapted with permission from 176. Copyright 2017, Elsevier. (C) Real-time *in vivo* imaging of nude mice with intravenous injection of CDs. (D) Real-time *ex vivo* imaging of nude mice with intravenous injection of CD. Adapted with permission from 182. Copyright 2017, John Wiley and Sons.

Some CDs have been reported to be mainly and easily excreted through the kidney filtration system (Figure 8A) 175. Du et al. showed a novel therapeutic nanosystem based on gadolinium-doped CDs (Gd-doped CDs) can quickly accumulate in the kidneys and be cleared within 12 h (Figure 8B) 176. Song groups found that the majority of CDs are excreted from the kidneys within 24 h 177. In zebrafish, the CDs prepared by employing glucose and ethylenediamine as the precursors and passivated agent can be excreted through the gut partly and mainly by the kidneys 178. It was reported that 65% of CDs synthesized by employing watermelon juice were excreted from the urine within 6 h post-injection, while the highest fluorescence intensity was displayed in the liver after 24 h and tended to have dwindled after 48 h (35%). These results revealed the major renal clearance and minor hepatobiliary excretion for CDs 179. It was found that the CDs were mainly accumulated in the kidneys at 1 h after injection via three different routes (intravenous injection, subcutaneous injection, and intramuscular injection) and almost completely excreted from the kidneys within 24 h, while only a small amount went to the liver 180.

Additionally, some CDs are cleared from the body mainly through feces. Singh et al. reported that the CDs synthesized by using an aqueous extract of beetroot were mainly cleared from the body by feces when administered by tail vein injection in mice 181. The strong fluorescence signal of CDs synthesized from o-Phenylenediamine in the urine, liver upper segment and small intestine of mice after tail vein injection was gradually reduced with time. The fluorescence signal in the dissected gastrointestinal tract was moved to the lower part of the intestine, indicating that the CDs could be drained from the biliary tract to the duodenum and then excreted in the form of feces (Figure 8C-D) 182. In fact, the large NPs (more than 20 nm) subjected to a high capture by the reticuloendothelial system (RES) and macrophages, and rapidly accumulated in the mononuclear phagocytic systems such as liver and spleen, resulting in a quick excretion from the body by the feces 183. According to the clinic requirement, the CDs can be modified to convert their excretion pathway. For example, PEG-modified CDs greatly reduced the capture of RES 36 and thus be quickly and effectively removed from the body through urine excretion.

### 4.6. Lysosomal escape

**Figure 9:**
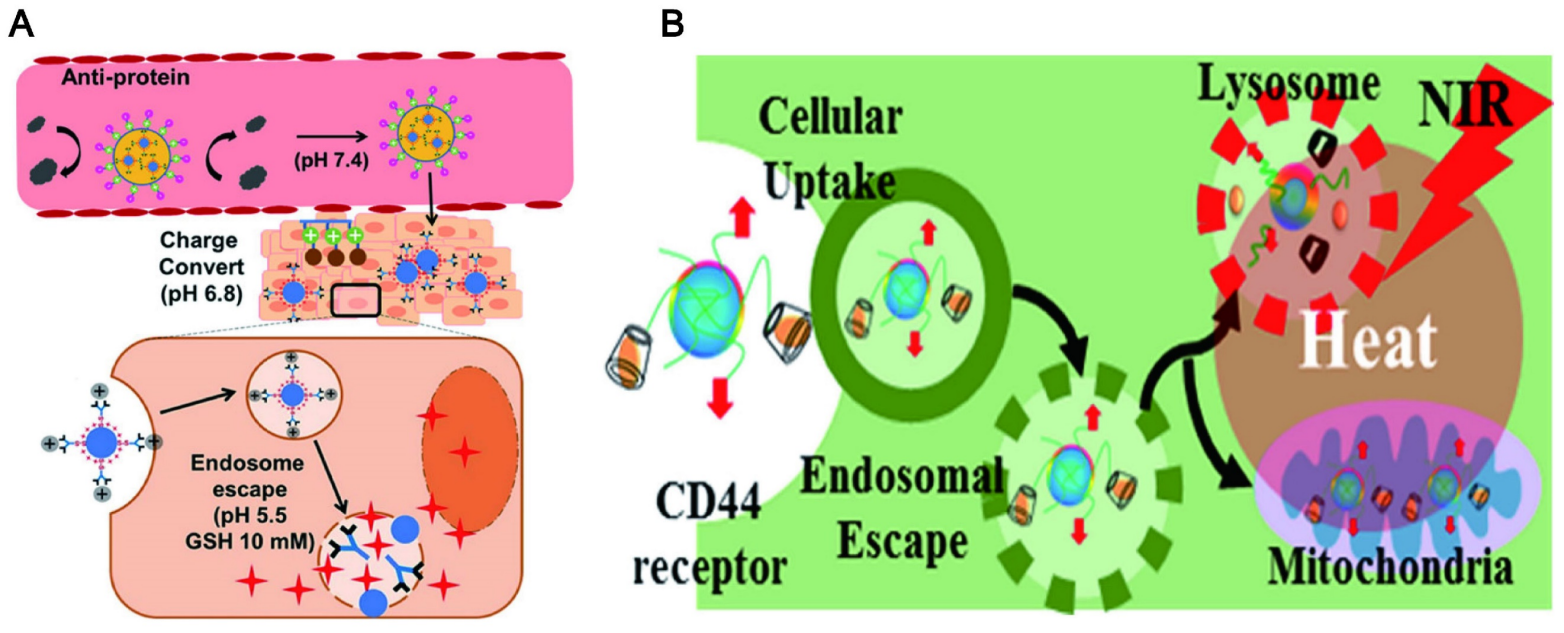
Lysosomal escape of CDs. (A) Endosomal escape of the CDs at low pH conditions and high GSH concentration. Adapted with permission from 185. Copyright 2018, American Chemical Society. (B) The target delivery of CDs via endosomal escape. Adapted with permission from 187. Copyright 2018, American Chemical Society.

Generally, the failure of many nanomaterials for delivering drugs is contributed to that they are easily captured by the macrophages and then accumulate in lysosomes 184. This phenomenon prevents the NPs from reaching the target in a complete form. It is a promising characteristic for drug delivery systems to escape from lysosomal degradation. Black tea synthesized CD showed a clear and uniform distribution in the nucleus and cytoplasm of HeLa cells, and a few typical spotting of lysosomal accumulations 168. This indicated that the CDs can avoid lysosomal entrapment. The escape of CDs from the lysosomal is up to the design of CDs and is also closely related to a functional modification. The zwitterionic decoration poly(carboxybetaine methacrylate) 89 was widely used to modify CDs for lysosome escape by reducing nonspecific protein adsorption. This is ascribed to the facile protonation of the surface guanidine groups of functionalized CDs in acidic media (endolysosome). The proton sponge effects ensure efficient endosomal escape and rapid drug release at low pH conditions and high GSH concentration (Figure 9A) 185. It was also reported that coating with a macrophage membrane on the surface of CuSCD (hollow-structured CuS NPs composed of CDs) can predominantly accumulate in the liver and spleen at a relatively low level due to the escaping ability from RES 186. In another way, the protonation of certain amino acids at low pH can trigger the interaction between the peptide and the endosomal lipid bilayer to form pores, which led the membrane fusion and lysis resulting in endosomal escape (Figure 9B) 187.

## 5. Merits of emitting LW-CDs for tracking and imaging

### 5.1. Deep tissue penetration

**Figure 10:**
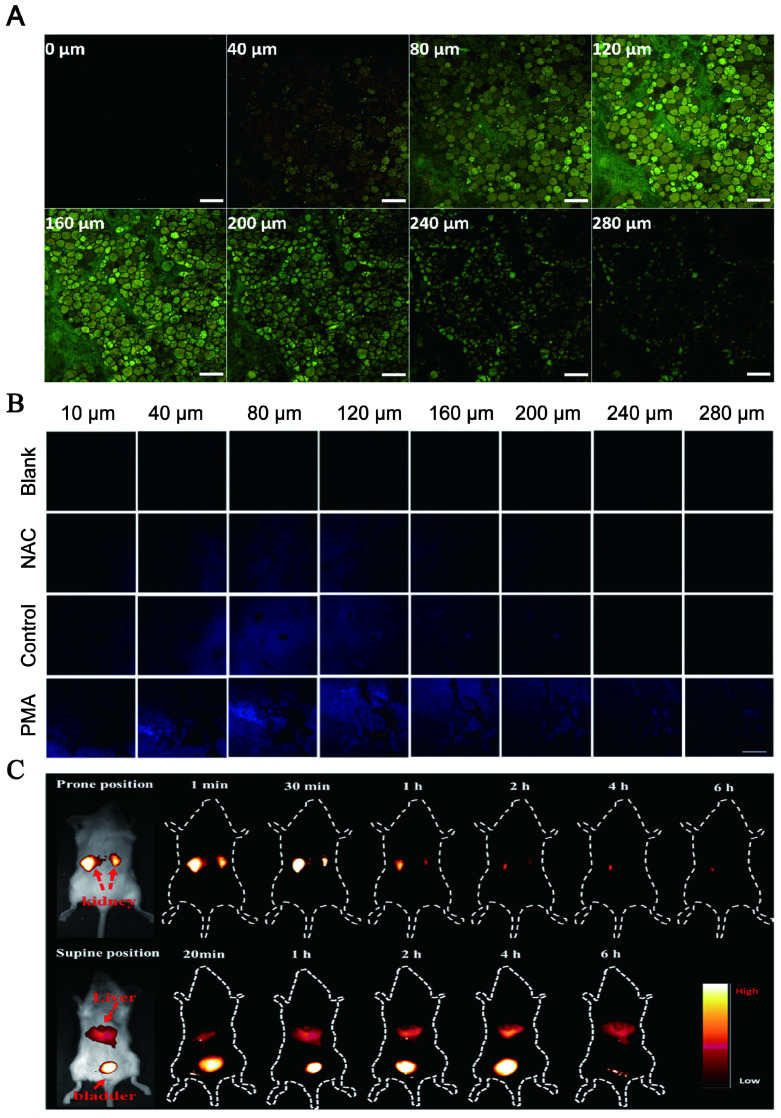
Deep tissue penetration of LW-CDs. (A) Light-tis two-photon-excited Z-stack images of pigskin tissue with an excitation wavelength of 750 nm (scale: 200 μm). Adapted with permission from 188. Copyright 2020, American Chemical Society. (B) fluorescence imaging of liver tissue slices. The scale bar is 200.0 μm. Adapted with permission from 189. Copyright 2021, Royal Society of Chemistry. (C) Time-dependent bioimaging of mice treated with CDs (20 μg/g) through the tail vein. Adapted with permission from 187. Copyright 2019, American Chemical Society.

Optical imaging has traditionally lacked penetration depth when used in the visible spectrum due to light-tissue interactions. Multiphoton NIR fluorescent technology has been explored to help optical bioimaging break through the limitation of imaging depth from 100 μm to 2 mm with very high fluorescence excitation collection efficiency. NIR CDs are promising candidates for *in vivo* imaging due to their deep tissue penetration. Lesani et al. developed a two-photon double-emission fluorescent multifunctional probe by conjugating fluorescein isothiocyanate on N-doped CD surfaces. The versatile probe showed excellent high-resolution imaging capabilities in deep tissue with penetration depths up to 280 μm in porcine skin tissue by using two-photon fluorescence imaging (Figure 10A) 188. Zhong et al. developed a two-photon carbon quantum dot-based dual-mode bioimaging nanoprobe for two-photon fluorescence imaging of endogenous H_2_O_2_ in the tumor microenvironment with a two-photon tissue penetration depth of 280 μm (Figure 10B) 189. Similarly, the near-red CDs synthesized by Liu et al. have a tissue penetration depth of about 240 μm 190.

Although two-photon-induced NIR emission of CDs has excellent penetration depth, its use is limited by the excitation of expensive and complex femtosecond pulsed lasers. NIR emission wavelengths are generally divided into two windows: NIR-I (650-900nm) and NIR-II (900-1700nm). Conventional NIR wavelengths (NIR-I, 650-900 nm) are considered the first biological window because it reduces the absorption and scattering of NIR by blood and water in living organisms. The signal-to-noise ratio (SNR) of bioimaging can be significantly improved in the NIR second region (NIR-II, 900-1700 nm), also known as the second biological window. The study found that NIR-II emission CD with high QY and good biocompatibility under the excitation of the NIR-I window can be realized and the application is more rapid and convenient. Li and his group presented a facile method to synthesize CDs with efficient NIR-II emission (EM: 925 nm) under 808 nm laser excitation by using watermelon juice as the carbon source (Figure 10C) 187. The CDs not only have good tissue penetration depth but also can be used for *in vivo* PTT therapy due to their high PT conversion efficiency. The study also found that compared with NIR-I PTT, NIR-II PTT can penetrate about 1 cm thick tissue under 1064 nm laser, so it has a good therapeutic effect on non-superficial tissues and tumor tissues with relatively complete vascular systems *in vivo* 186.

### 5.2. Low photon scattering

The photon penetration mainly relies on light scattering, absorption, and spontaneous fluorescence of tissue during fluorescence imaging of mammalian tissues. The high photon scattering will limit its biomedical applications. According to Mie theory and Monte Carlo simulations of scattering, the photon scattering scale is described as μs′ ≈ λ^-*w*^, where *w* ranges from 0.2 to 4 in different tissues 191. Therefore, fluorescence imaging in the NIR window with longer wavelengths can achieve lower photon scattering. Consequently, the increasing demand for NIR fluorescence with strong penetration in deep tissues and reduced tissue absorption means that fundamental science needs to make major advances in better imaging instruments and new fluorophores.

**Figure 11:**
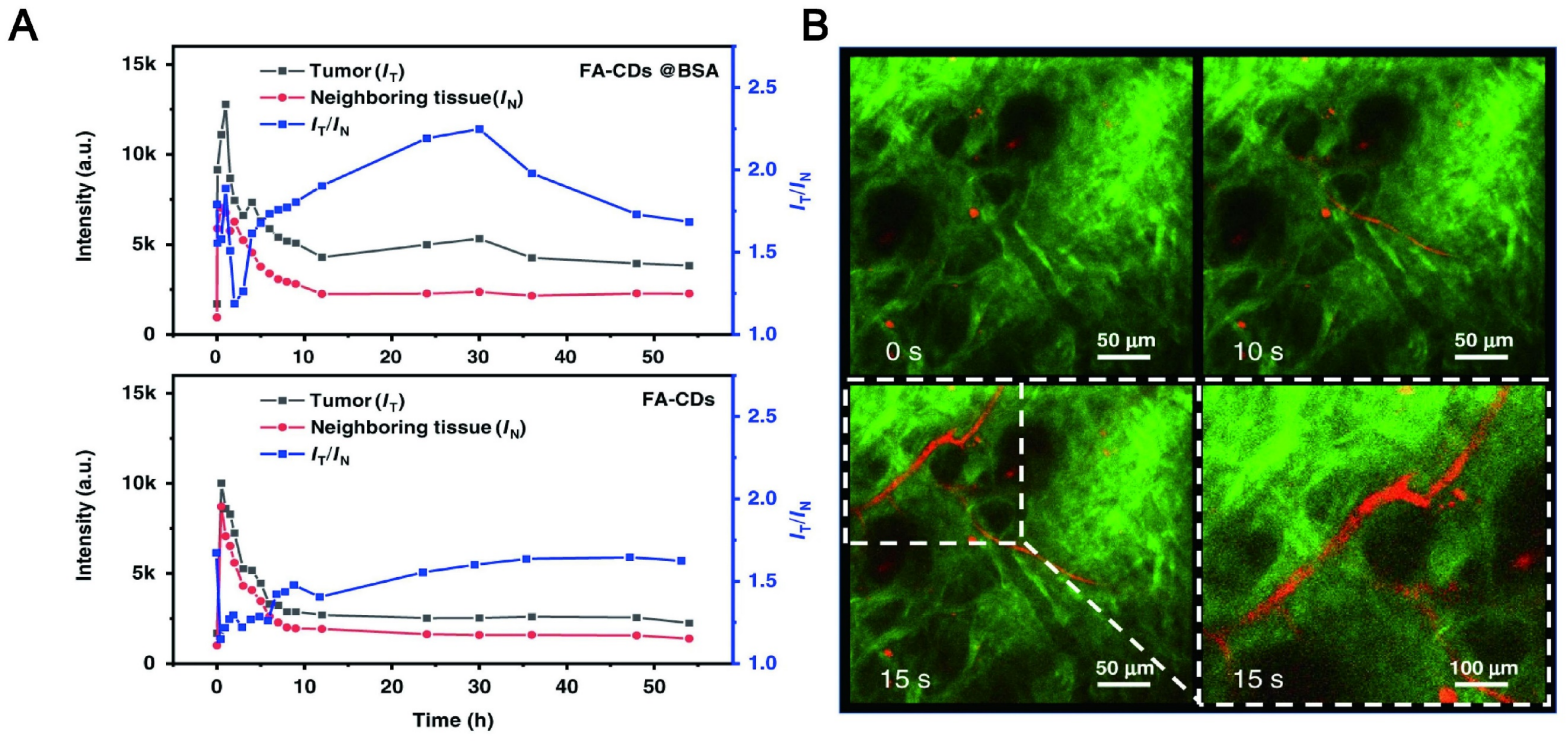
Low photon scattering LW-CDs. (A) STED image and (B) confocal image of a representative A549 cell stained by Ni-pPCDs. B. Resolution of IFM image is 200 nm pixel^-1^. Adapted with permission from 192. Copyright 2022, Springer Nature.

NIR CDs as a new type of fluorescent agent that may be a promising candidate for reducing photon scattering. Zhang synthesized two-photon and three-photon fluorescent CDs with emission wavelengths of approximately 680 nm and 690 nm via bovine serum albumin-bound pure red-emitting CDs excited by FS pulsed lasers at 1150 nm and 1550 nm. The CDs can be uniformly dispersed in water. After intravenous administration to mice for 12 hours, the FL signal contrast (IT/IN) of the red FL intensities in the tumor site (IT) and near tissue (IN) of the CDs-treated mice can reach a value of greater than 2, which means that tissue absorption/scattering and background interference can be almost negligible (Figure 11A-B) 192. Zhao and his team found that stable LW fluorescence-emitting CDs synthesized by using an appropriate carbon source glutathione can clearly distinguish the strong autofluorescence of biological samples. In the mouse model, the concentration of CDs showed a good positive correlation with fluorescence intensity, and brighter signals were obtained for up to 22 hours. As a control, the Cy5 probe exhibited a relatively much lower signal intensity due to interference and absorption of the tissue background 193. Zhang et al. synthesized CDs with a NIR emission band at 745 nm by fusing large conjugated perylene derivatives under solvothermal treatment. Both two-photon NIR angiography and *in vivo* NIR fluorescence bioimaging showed excellent imaging capabilities of the CDs and low tissue background absorption 194.

### 5.3. High contrast resolution

**Figure 12:**
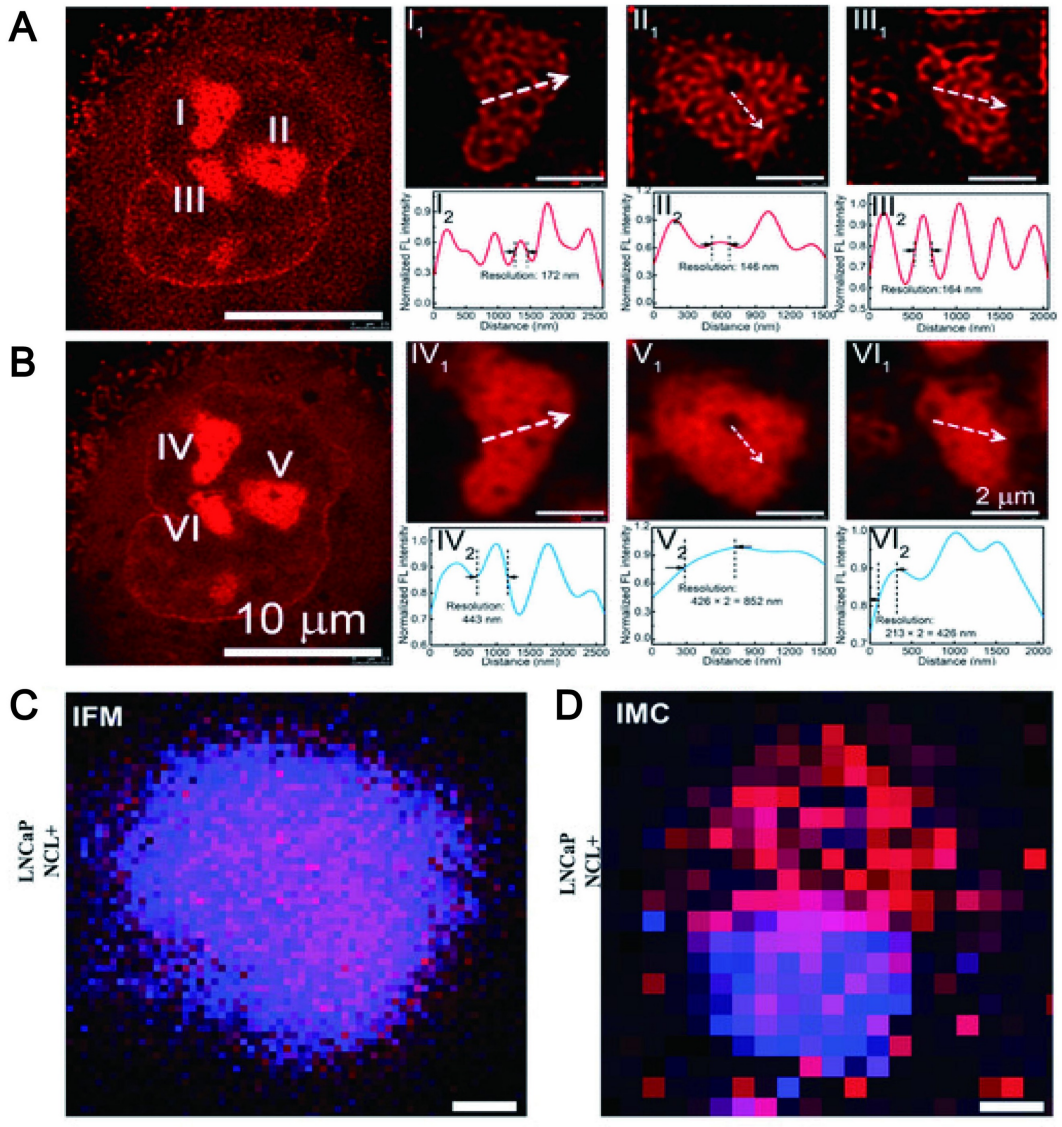
High contrast resolution of LW-CDs. (A) STED image and (B) confocal image of a representative A549 cell stained by LW-CDs. Adapted with permission from 202. Copyright 2019, American Chemical Society. (C) Resolution of IFM image is 200 nm pixel-1 and (D) IMC image is 1 µm pixel-1. Adapted with permission from 203. Copyright 2021, John Wiley and Sons.

*In vivo* imaging with a high spatial and temporal resolution is an effective strategy for the development of new therapeutics for disease such as assessment of the vasculature and hemodynamics of small vessels. It is well known that conventional fluorescence imaging methods enable visualization of individual aggregates with a spatial resolution of approximately 250 nm. However, it is difficult to characterize species beyond the optical diffraction limit. At present, the emergence of many super-resolution imaging techniques, such as stimulated emission depletion microscopy, light-activated localization microscopy, stochastic optical reconstruction microscopy, structured illumination microscopy, etc., has overcome the diffraction limit, greatly improving spatial resolution. The key to overcoming the resolution limitations imposed by diffraction is based on the random switching of individual fluorophores between the "on" (fluorescent) and "off" (non-fluorescent) states. In this method, high-precision positioning requires a low-duty cycle and high brightness 195. Currently, some fluorophores have been extensively studied such as organic dyes 196, fluorescent proteins (FP) 197 and quantum dots 198. However, for organic dyes, thiols and oxidants are required to tune the blinking behavior of the fluorophore. These additives enable fluorophores to achieve efficient blinking behavior, but can also cause strong cytotoxicity 199. FPs are always required for efficient protein expression and tend to dimerize or aggregate, leading to artifacts in protein localization 200. For QDs, being in a state for long periods can lead to poor localization 201. Additionally, the applications of other organic molecules and inorganic nanomaterials are limited by poor fluorescence stability and toxicity. The study found that CDs formed by carbon-based nanomaterials can display high-resolution imaging *in vivo* by emitting NIR fluorescence (700-1400 nm) to achieve precise spatial temporal resolution. For example, fluorescent CDs synthesized by using phenylenediamine (pPDA) with emission wavelengths up to 700 nm can be formed by adding nickel ions (Ni^2+^) as catalysts. The red wavelength-emitting CDs enabled nucleolar targeting, and the unique fluorescence emission of Ni-pPCD endowed it with imaging resolutions up to 80 nm. Zooming in on the marker positions revealed full widths of 172, 146, and 164 nm, which were significantly better than 443, 852, and 426 nm in conventional confocal images (Figure 12A-B) 202. The lanthanide-doped CDs (LD-CDs) as fluorescent probes can emit at a wavelength of 627 nm. LD-CDs labeled EpCAM and NCL proteins in LNCaP cells to provide more precise spatial resolution for these functional proteins at optical resolution (200 nm pixel-1) (Figure 12C-D) 203.

In addition, researchers have proposed that one-photon fluorescence may suffer from several drawbacks: poor photostability, easy photobleaching, and shallow tissue penetration. Two-photon fluorescence can increase penetration depth, suppress background interference, and have a higher spatial and temporal resolution. Studies have found that CDs synthesized using 2,4-Diaminotoluene, FA and betaine allowed direct observation of chromatin structure with sub-diffraction resolution (90 nm) at very low excitation (<1 μW) and depletion power (<5 mW). The dual-mode nanoprobes based on two-photon red/NIR fluorescence CDs greatly benefit the spatial resolution of imaging 204. Similarly, Zhong et al. developed a two-photon CD-based dual-mode bioimaging nanoprobe for two-photon fluorescence imaging of endogenous H_2_O_2_ in the tumor microenvironment, with a two-photon tissue penetration depth of 280 μm 189.

### 5.4. High signal-to-background ratios

Signal-to-background ratios refers to the ratio between the net analysis signal and the background interference signal. Fluorescence in the visible spectrum produces background noise that results in a lower signal-to-noise ratio than bioluminescence. Although different techniques can be used to separate the background light, it is difficult to completely eliminate the background noise due to the limitation of fluorescence characteristics. These background noises result in lower sensitivity of *in vivo* imaging systems. Compared to the visible spectrum widely used for biological imaging, the broadly defined NIR region can provide deeper tissue optical imaging and improve signal-to-background ratios. Zhang et al. used small aliphatic molecules to obtain R-CDs (EW: 646 nm) with PLQY up to 65.5% by introducing an electron-donating source and controlling the doping amount of graphitic nitrogen. A single longitudinal mode solid-state CD laser with a signal-to-noise ratio of 14.8 dB is realized 205. CDs exhibit higher signal-to-noise* in vivo* as the emission wavelength increases. The study found that when the emission wavelength of CDs was increased to 745 nm, the bright NIR fluorescence signal from the mouse intestine after gavage injection exhibited a high signal-to-noise ratio (S/N) of up to 18, which proved that in deep tissue penetration. Transparent *in vivo* NIR imaging demonstrates its ability to penetrate deep tissue 184.

It is worth noting that the specific targeting of NIR fluorescent CD-binding functional materials may be a good strategy for the visualization of lesion sites with high spatial resolution and signal-to-noise ratio. The study found that hyaluronic acid as the natural ligand of CD44 receptor-conjugated carbon quantum dots can achieve targeted aggregation of breast cancer cells. This CD achieved a high level of tumor specificity via local or systemic injection, as judged by a strong signal-to-noise ratio between the tumor and surrounding tissue *in vivo* 206. Similarly, tumor cells (MCF-7 and K150) can be targeted by using transferrin-conjugated CDs to realize *in vivo* visualization with a high signal-to-noise ratio 207.

Overall, NIR CDs are more attractive for *in vivo* imaging. NIR CDs can effectively enhance tissue penetration depth, reduce light scattering, increase the spatial resolution of local tissues and improve the signal-to-noise ratio, etc., making them promising candidates for excellent imaging agents in the future.

## 6. Factors affecting the LW emission of CDs

An outstanding feature of CD_S_ is the PL characteristics. Nevertheless, the majority of CDs emit in the visible region range from blue to orange, which extremely impedes their application in life science. The intense emission of CDs in far-red and NIR regions has attracted more attention from researchers. The red luminescence mechanism of CDs is not yet fully understood due to the diversity and complexity of the structure and composition of CDs. The factors affecting the luminescent properties of CDs can be roughly divided into the following points concluded from the existing studies: synthesis method, precursors, size, surface state, concentration and pH value of CDs. Therefore, systematically summarizing the factors affecting the infrared luminescence of CDs is an important strategy for the efficient acquisition of CDs suitable for *in vivo* image applications.

### 6.1. Synthetic strategy

Generally, the synthetic route of emitting LW-CDs can be divided into two main methods: top-down and bottom-up strategies. Top-down approaches are achieved by exfoliating small carbon NPs from larger mass carbon materials, for instance, arch discharge, laser ablation, chemical oxidation synthesis, etc. The bottom-up approaches involve the preparation of CDs by stepwise chemical fusion of small molecules or polymers after dehydration and carbonization 208,209. It is reported that the synthetic routes including precursors and reaction methods have a great influence on the fluorescence performance of the prepared CDs 210,211. The precursors and the preparation process of the aforementioned LW-emissive CDs are summarized to find some regularities for guiding the synthesis of CDs (summarized in Table 6). Among the various synthetic methods, the hydrothermal method is the most widely used because it is simple to prepare and suitable for laboratory research without the requirement of complicated equipment, followed by the solvothermal method. Except for the synthetic routes, a suitable carbon source is very critical for the optical performance of CD 212. It was reported that the appropriate aromatic structure is beneficial to LW emission which can inherently reduce the energy gap by creating a large sp^2^ domain. Researchers discovered that the introduction of the aromatic molecules of dopamine (DPA) can cause a significant red shift (emission 710 nm) of CDs fabricated by oPDA and DPA 213, while the CDs derived from pPDA and aspartic acid (ASP) emitted 535 nm 214. The CD synthesized by using 2,5-diaminotoluene sulfate (DATS), a similar structure to pPDA, had a red emission performance 215. The CD prepared by CA and ethanolamine showed both molecular state and carbon nucleus state 216, while the CDs prepared by CA and ethylenediamine showed three different emission types of molecular state, aromatic domain state, and carbon nucleus status 217. Therefore, various precursors have a dramatic impact on the mechanism of luminescence and further affect its LW emission performance.

### 6.2. Particle size

**Figure 13:**
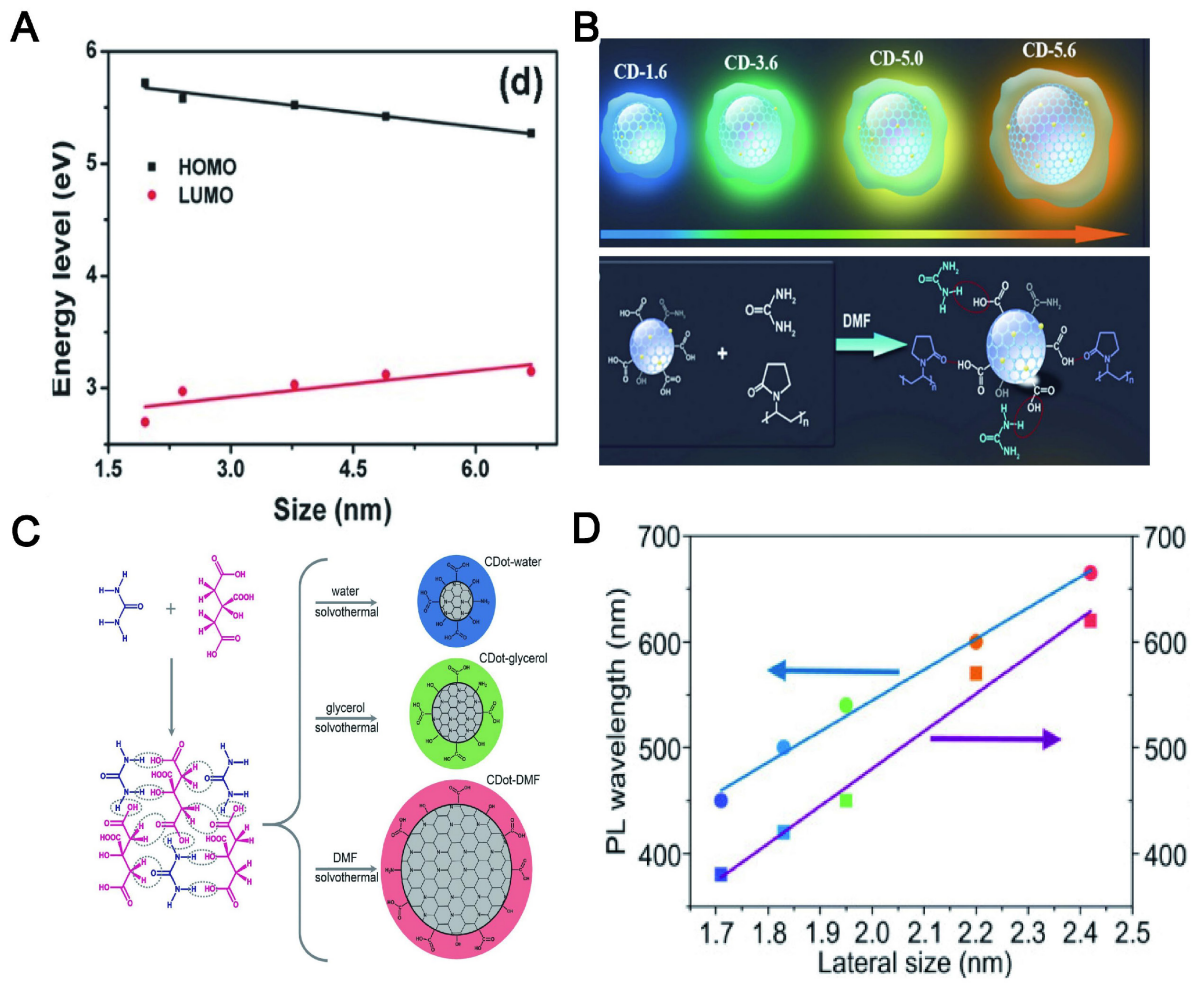
The influence of size on the red shift of CDs. (A) The increase in the particle size leads to a red shift of the emission wavelength by reducing the energy gap of CDs. (B) Schematic representation of four different sizes of CDs with the different extent of π-conjugated domains of the resulting CDs. The colors represent the real PL color of the CDs. Seeded growth reaction leading to the formation of CDs and particle size changes. Adapted with permission from 246. Copyright 2021, John Wiley and Sons. (C) Correlation between HOMO and LUMO energy levels of CDs and their particle sizes. The downtrend in HOMO and LUMO levels leads to red shift of CDs. Adapted with permission from 247. Copyright 2021, John Wiley and Sons. (D) The degrees of dehydration and carbonization are gradually increased in water, glycerol, and DMF, resulting in the increased sp2-domains and red-shifted absorption and emission bands, which agree well with their increased particle sizes. Adapted with permission from 248. Copyright 2021, John Wiley and Sons.

Although the PL mechanism of CDs is still under debate, the research community has temporarily accepted two possible emission sources. One of them is the state of the carbon nucleus based on the band gap, which contains conjugated π domains. For this mechanism, the size of CDs, or more accurately the size of the sp^2^ domain contained, will affect their PL position. The other is functional groups/structures on CDs surface, which is described in the following "surface state". The increase of the particle size can reduce the energy gap of CDs, resulting in a red shift of the emission wavelength, which is mainly due to the band gap transition of the conjugate π domain in the sp^2^ carbon structure core (Figure 13A) 245. For instance, the color-tunable fluorescence of CDs under UV excitation was realized by augmenting the size, whose color depended on the size of the π-conjugated domains (Figure 13B) 246. Moreover, the emission band of CA-CDs was red-shifted from 448 to 550-638 nm 247 when the particle size was increased from 1.7 to 2.8-4.5 nm (Figure 13C). A similar phenomenon was reported that the corresponding PL wavelength of CDs using o-phenylenediamine as the precursor was shifted from blue to red with its size increased from 1.7 to 2.4 nm (Figure 13D) 248. In short, the "particle size" here refers to the internal effective conjugation length or sp^2^ domain size in the carbon core that results in a change in the π-π* energy gap, exhibiting size-dependent PL emission behavior, rather than the physical size of the CD.

### 6.3. Surface state

The surface state refers to the formation of the quantum state of the surface electrons into discrete energy levels or very narrow energy bands due to its defects, adsorption of matter and other reasons. The LW emission of CDs is the relevant functional groups/structures on its surface. However, the surface state is complicated because it involves the graphite nuclei within the CDs and the surface functional groups. It has been proposed that all CDs have a common sp^2^ domain core and are surrounded by chemical groups rich in N and oxygen on the surface 249. The fluorescence emission mechanism of CDs has revealed that the energy transfers from the initially excited graphite nuclei to surface defects and emits fluorescence 250. Surface defects are mainly caused by surface oxidation, which can serve as a trapping center for excitation and induce PL emission 219. Therefore, the higher the level of surface oxidation of the CDs, the higher the emission efficiency. The research exhibited that fluorescence emission originated from surface defects, which can cause the red shift through surface oxidation 251. Zhang et al. used a solvothermal synthesis route by using precursors rich in carboxylic acid and amine to prepare CDs and found that when the content of oxygen-rich groups was increased from 17% to 29% and the oxygen-carbon ratio was increased from 0.20 to 0.34, the emission of CDs was shifted from blue to red, indicating that the emission wavelength of CDs is positively correlated with the degree of oxidation 250. Jiang et al. reported that CDs with and without tartaric acid (TA) rich in carboxylic emitted green and red fluorescence, respectively 252. Further research found that the addition of TA will raise the level of surface oxidation and carboxyl groups on the prepared CD, which was considered to be the reason for their emission of red shift. Because the band gap of the chemical structure strongly depended on the degree of oxidation and carboxylation. This is also consistent with previous studies that the carboxyl groups coupled to the sp^2^ carbon skeleton will cause obvious local distortions, thereby reducing their energy gap and causing a red shift 253.

**Figure 14:**
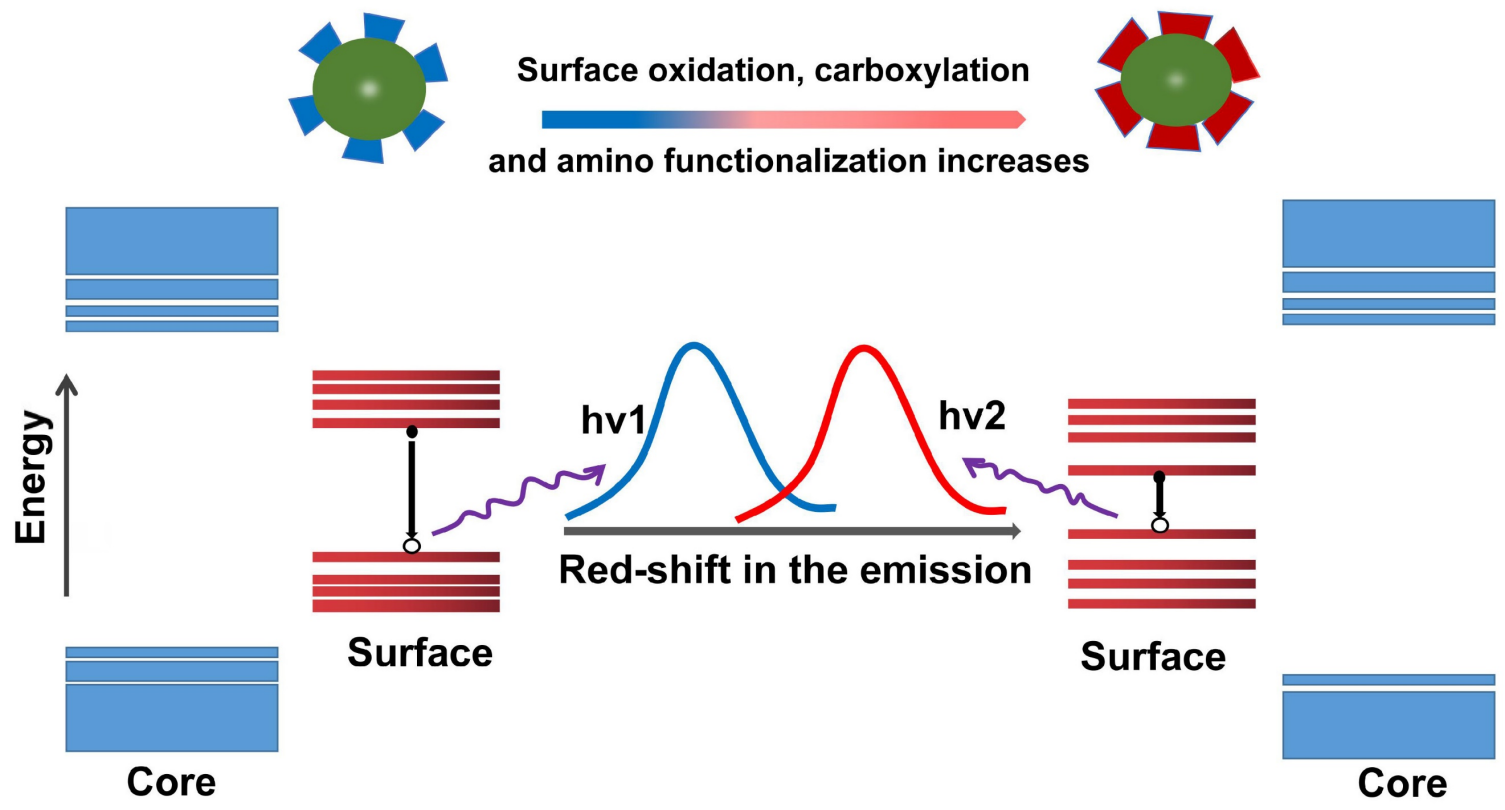
The influence of the surface state on the red shift of CDs. CDs undergo red shift and emit LWs through different pathways such as their surface oxidation, carbonization and amino functionalization.

The amino functionalization of CDs is another strategy for redshift. For example, the PL peak of the CDs synthesized by polycyclic aromatic hydrocarbons (PAH) molecules were transferred to longer wavelengths, which was positively correlated with the number of amino groups. The red shifts of the CDs contributed to the charge transfer from the highest occupied molecular orbital (HOMO) to the lowest unoccupied molecular orbital (LUMO) transition at high degrees of functionalization 254. Shao et al. controlled the amino functional groups on the surface of CDs by adding different proportions of urea as N sources and found that the redshift of the maximum emission fluorescence was increased when the number of amino groups increased 255. In summary, the surface state of the CDs is considered to be an important factor for adjusting the LW emission, which comes from the narrowing of the band gap (Figure 14).

### 6.4. Heteroatom doping

**Figure 15:**
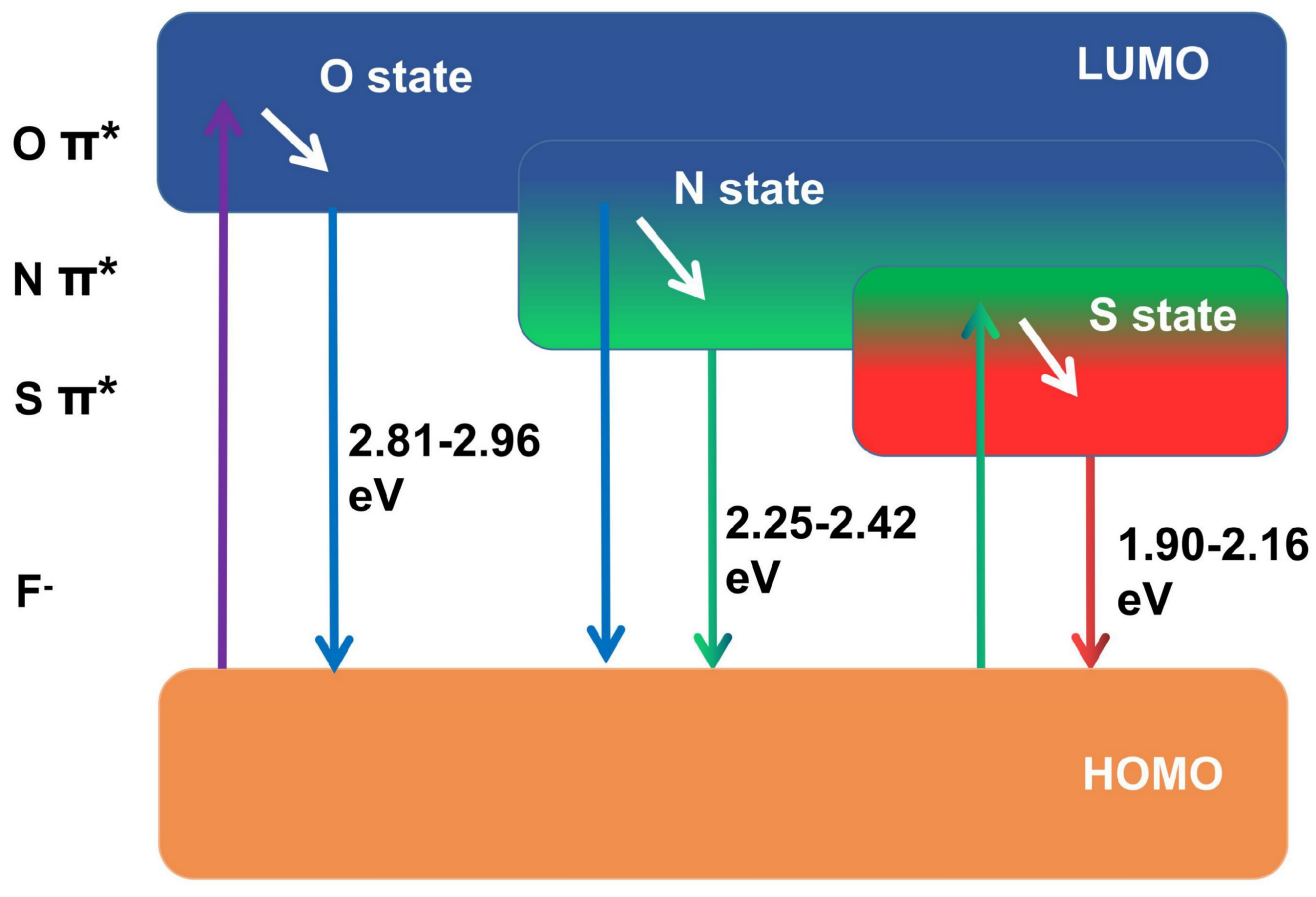
The influence of heteroatom doping on the red shift of CDs. The HOMO level of CDs increases with doping, which leads to a red shift.

The heteroatoms are relative to the main atoms C and H, which generally refers to fewer atoms N, S, P, etc. Heteroatom doping has been confirmed as a practical protocol to adjust the PL of the original CD. This mechanism is attributed to the red shift induction of heteroatom through modulation of the electronic effect of CD electronegativity (χ). Commonly used dopants include N, S, O, F, P, etc. These dopants are divided into electron withdrawing carriers (χ> 2.58) and electron donor carriers (χ< 2.58) according to the electronegativity value and carbon as the dividing point. In molecular orbital theory, the dopant in the host allows the introduction of energy states in the energy gap between the HOMO and the LUMO 256. For the doped CD, the lower χ is beneficial to the red shift spectrum due to the electron donor effect and the lower light excitation energy (Figure 15) 257. Senkovskiy et al. used polythiophene-3-boronic acid (PTB) to synthesize B-doped CDs with bimodal characteristics. Compared with B-free CDs, B-CDs showed obvious red-shifted fluorescence emission, which was due to the reduced energy gap caused by the surface defect state generated during the B doping process 258. A similar mechanism was found to be based on B doping and further preparation of B and N co-doped CDs. It was discovered that these co-doped CDs had higher fluorescence and tunable NIR photoresistance than N-doped CDs. Because the configuration of C and N in CDs was changed by B doping, surface defect states were generated 259. In addition, Bao et al. found that S doping introduced a lower energy level, thereby reducing the optical band gap and contributing to the strong absorption band from the red to NIR region and the NIR emission under excitation at 655 nm 175.

In addition to the mechanism of redshift caused by the above-mentioned electron donor group, as the electron-accepting group with the greatest electronegativity, F has a strong electron-withdrawing tendency, which can reduce the energy levels between the HOMO and LUMO and then tend to redshift 260,261. The F sources used for preparing F-CD include 4,5-difluoro-1,2-phenylenediamine, fluorinated diglycidyl ether, tetrafluoroterephthalic acid, fluorine sodium, ammonia fluoride, levofloxacin and 5-Fluorouracil 262-265. Dong et al. used 1,2-diamino-4,5-difluorobenzene as the F source to prepare F-CDs by the one-pot solvothermal method 262. F doping can realize the red- shift of the emission wavelength by at least 50 nm. Ye et al. used a microwave-assisted method to produce F-CD with red fluorescence. The fluorescence red shift was attributed to the reduction of the surface state energy gap caused by F-doping 265.

### 6.5. Solvent and concentration

**Figure 16:**
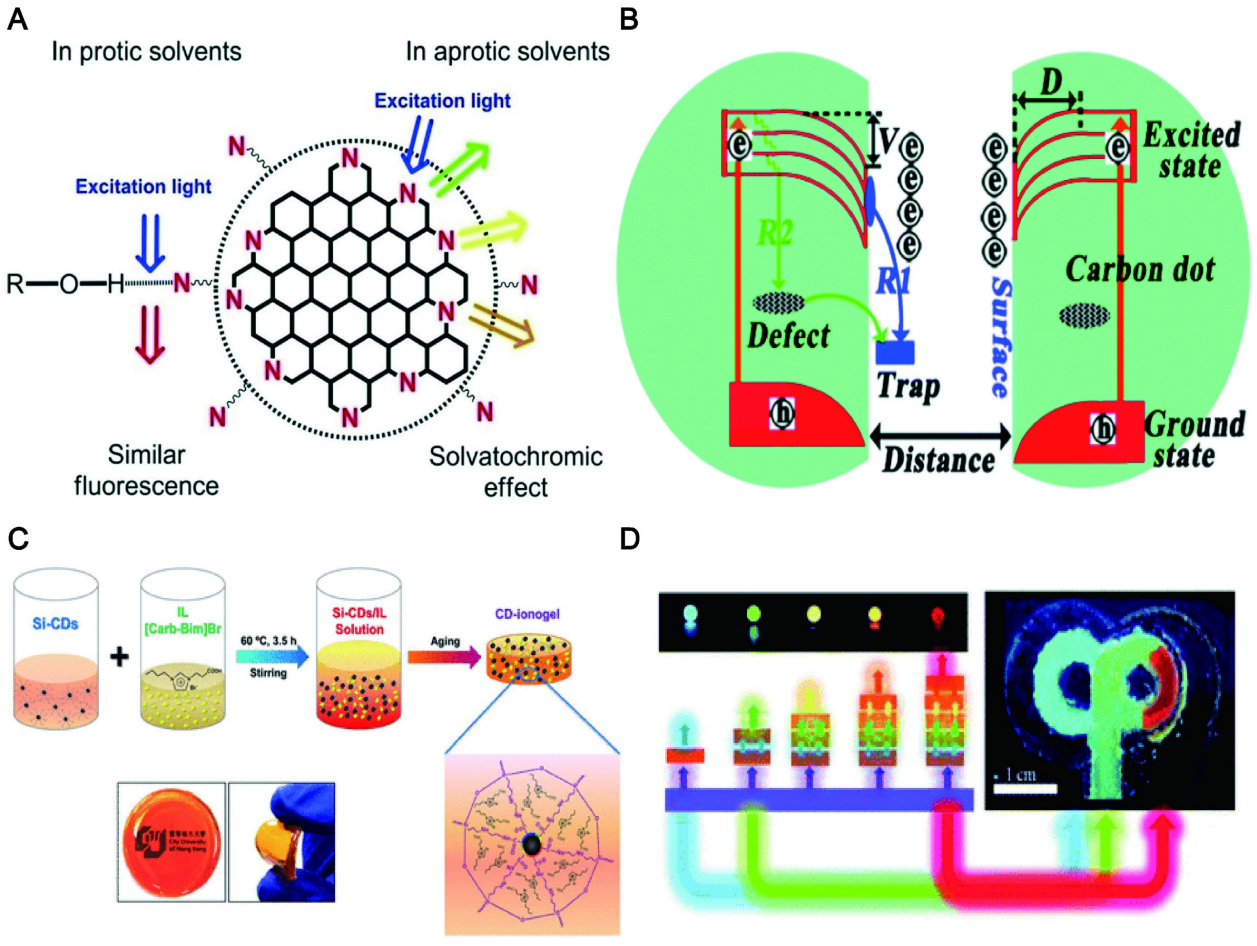
The influence of solvent and concentration on the red shift of CDs. (A) The solvent-dependent photoluminescence is regarded as a dipole-induced surface electronic state change. As the solvent polarity increases, the dipole moment affects the surface electronic structure and reduces the energy gap, resulting in the red shift of the fluorescence emission wavelength. Adapted with permission from 266. Copyright 2017, Royal Society of Chemistry. (B) Mechanism of PL emission tuning with concentrations. Concentration-induced morphological changes influence energy traps associated with surface states, which was a mechanism of CDs with excitation-dependent PL properties and red shift. Adapted with permission from 268. Copyright 2017, Royal Society of Chemistry. (C) Schematic illustration of Si-CDs dispersed in an ionogel at high concentrations. Adapted with permission from 269. Copyright 2014, American Chemical Society. (D) CDs with an emission color ranging from blue to red, which is measured by the thickness of the down-conversion layers and CDs doping concentration. Adapted with permission from 270. Copyright 2015, Royal Society of Chemistry.

Additionally, CDs exhibit solvent-dependent PL and concentration-dependent PL. For example, the CDs prepared by using p-phenylenediamine can emit broad-spectrum lights (ranging from dark green to red colors) independent of the excitation wavelength when it was dispersed in various solutions (H_2_O, acetone, CCl_4_, CHCl_3_, DMF, toluene and CH_3_OH). The emission wavelengths changed to red with the increase of solvent polaritydue to the surface electronic state induced by the dipole (Figure 16A) 266. It was found by Jing et al. that the irreversible blue shift of CA CDs prepared in protic solvents contributed to the high-pressure-induced additive reactions between the solvent molecules and the electron-withdrawing groups on the CD surface. As a control, the reversible redshift of CA CDs synthesized in aprotic solvents (N, N-dimethylformamide) furnished evidence of enhanced π-π stacking in the CD cores 267.

CDs exhibited diverse morphologies with the change of concentration in the solvent, resulting in regulable PL emission across the visible spectrum. The morphological changes induced by concentration influence the energy traps relevant to surface states, which reduced their surface electric potentials (V) and thus limited the electron shift to the surface. Therefore, the wavelength of CDs was shifted gradually from 630 to 400 nm as their concentration was decreased in solution (Figure 16B) 268. The research found that the thickness (0.1-10mm) of the CD-ionogel synthesized by a sol-gel method achieved a full-color fluorescence emission. This remarkable red-shift effect was attributed to the combination of light multiple absorptions and the excitation-dependent PL of Si-CDs dispersed in an ionogel at high concentrations (Figure 16C) 269. Sun et al. reported that the thickness of the down-conversion layers and the doping concentration of CDs in the polymer matrix determined the emission colors ranging from blue to red (Figure 16D) 270. Due to the tunable PL (regulated to far-red/NIR) of CDs, it will be beneficial for the targeted tracer imaging of different tissues and organs in the body.

### 6.6. Other factors

**Figure 17:**
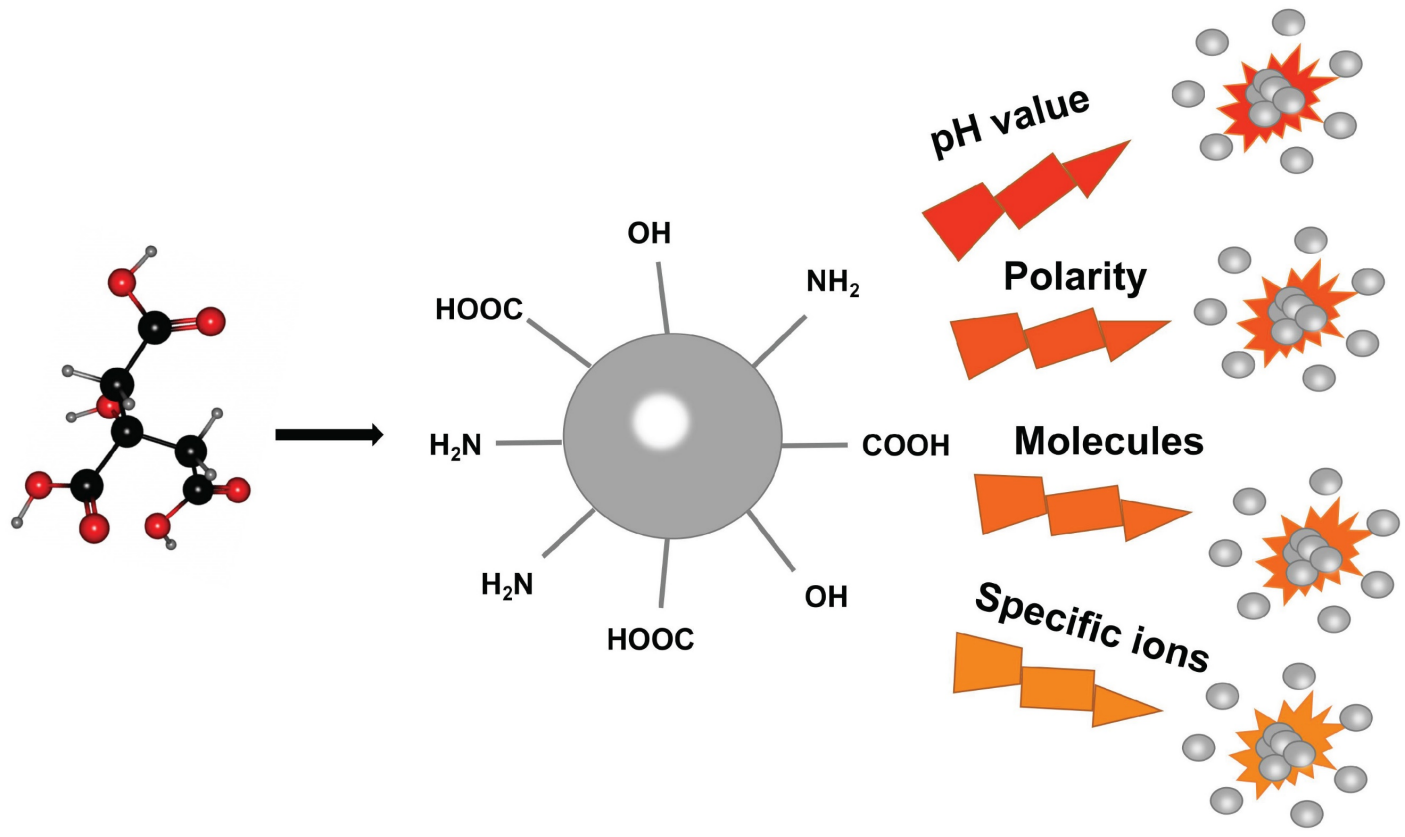
The influence of other factors on the red shift of CDs. The CDs exhibit a red shift unrelated to excitation and a red shift of CDs commonly related to the surrounding environment such as pH value, polarity, molecules, or specific ions.

In addition to the factors mentioned above, the pH value 271, polarity 272, molecules (e.g., ascorbic acid 273 or sugar 274) or specific ions (e.g., Ni^2+^ 50 or Cu^2+^ 275) of the surrounding environment will affect the PL characteristics of CDs (Figure 17). Ju et al. reported that the N-CDs fabricated by employing o-phenylenediamine with a facile method under acidic conditions emitted red fluorescence while the N-CD synthesized under neutral and alkaline conditions emitted orange fluorescence 271. The emitting red light of N-CDs under acidic conditions may be contributed to the protonation and deprotonation of N doping in rigid carbon framework structures 276. Hua and his group studied the effects of various metal ions of Ag^+^, Cu^2+^, PtCl_4_^2-^, and Ni^2+^ on the fluorescence of CDs. The CDs synthesized by using Ni^2+^ as a precursor exhibited excitation-independent emission (nearly 605 nm), excellent photostability and polarity sensitivity 50. Wu et al. proposed that the excitation wavelength-independent red emission band can be controlled by the polarity of the solvent through intermolecular hydrogen bonds between the solvent and the CDs 272.

## 7. Visualization tracking and target delivery

### 7.1. Disease diagnosis

Early detection is a critical stage of prognostic strategies for various life-threatening diseases. The mortality rate could be enormously reduced if they are diagnosed at an early stage. As a novel carbon-based material for fluorescent emitting, LW-CDs have become one of the most effective means to detect the pathological microenvironment *in vivo* due to their excellent luminous, transmembrane transport performance, good biocompatibility, low toxicity, as well as visual assessment of biomarkers for disease. The surface-doped or functionalized CDs solve the issue of being unable to image other nanocarriers (e.g., liposomes, micelles, dendrimers, and meso porous silica NPs) as well as the instability and toxicity of fluorescent dyes and semiconductor quantum dots* in vivo*. Peng et al. reported that the CDs synthesized from carbon nano-powder had high affinity and specificity combined with calcified bones of zebrafish. The chemical modification of CDs by EDC/NHS did not interfere with its bone binding affinity and specificity *in vivo* and achieved stable imaging *in vivo* 277. This was consistent with Li's report that CDs had a specific affinity with calcified bones for a marked enhancement of luminescence, which is critical for identifying, diagnosing and treating bone diseases 278. Cao and his research groups also demonstrated the potential diagnostic application of CDs *in vivo* by tracking and imaging. In his study, a relatively straightforward demonstration was to track the migration of CDs through lymph vessels in mice following the paw injection. The strong fluorescence emissions from the CDs were observed in the axillary lymph nodes 24h post-injection, further confirming that the CDs had the migration performance with preservation of their fluorescence properties *in vivo* 279. This also revealed the rationality of CDs being used as benign contrast agents for optical biological imaging.

**Figure 18:**
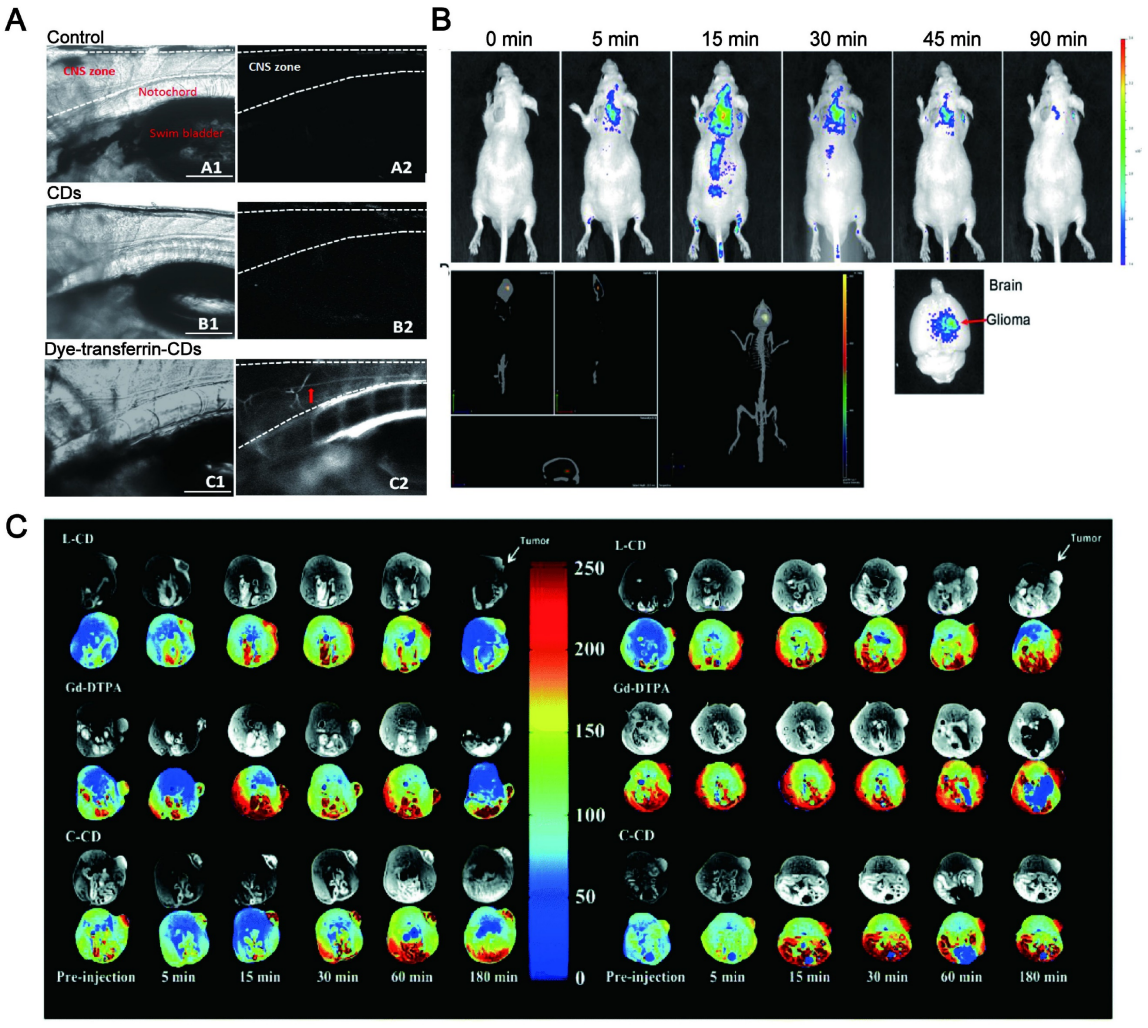
*In vivo* diagnostic applications of CDs. (A) The bright CDs can be visualized in the central nervous system, which provided a strategy for the diagnosis of diseases in the brain. Adapted with permission from 282. Copyright 2016, Elsevier. (B) *In vivo* and *ex vivo* imaging of glioma-bearing mice after tail intravenous injection of CDs. Adapted with permission from 285. Copyright 2015, American Chemical Society. (C) The *T*_1_ weighted MR phantom images (A) of CDs and Gd-DTPA via tumor injection (left) and intravenous injection (right) at different times. Adapted with permission from 303. Copyright 2019, Royal Society of Chemistry.

More importantly, the CDs are visualized in the diseased parts via target migration by functional modification 280. For example, CDs with abundant amino functional groups had good lysosomal targeting performance and thus achieved ultra-fast lysosomal imaging of cells in zebrafish to monitor the apoptosis status of cells. Thus, it can provide aidance for early diagnosis of neurodegenerative diseases, lysosomal storage diseases and several immune system diseases 281. It was more interesting that CDs rich in carboxyl groups conjugated with transferrin can across the blood-brain-barrier (BBB) in zebrafish through receptor-mediated endocytosis 282,283 and thus the bright CDs can be visualized in the central nervous system (CNS) (Figure 18A). This provided another strategy for the diagnosis of diseases in the brain. It was also reported that CDs synthesized by using tryptophan 284 or aspartic acid can enter the brain tissue without further receptor -conjugation (Figure 18B) 285. Therefore, it demonstrated a great potential that CDs can be used as imaging diagnose for brain diseases. Importantly, CDs might be promising materials for cancer diagnosis. Yang et al. described that positively charged CDs synthesized by FA and PEI through a one-step hydrothermal method can effectively reduce the cytotoxicity of PEI and selectively image the folate receptor (FR) in biological systems to isolate positive cells from normal cells 286. Song's team also reported that FA-combined fluorescent CDs could be used to distinguish the HeLa cancer cells 287. Liu et al. modified the CD with FA to enhance the optical spatial resolution of cell imaging 288. Other studies have also confirmed that fluorescent CD combined with FA can be used as a recognition tag to identify cancer cells 289,290. Recent studies revealed that the luminescent FA-CDs prepared by active dry yeast can noninvasively penetrate the cell membrane and enter the cytoplasm of HepG2 cancer cells through receptor-mediated endocytosis. FA-CDs can effectively identify FR-positive cancer cells from the normal cell mixture 291. CDs can be also used for the specific identification of other cancer markers such as microRNA-2 292, exosomes 293, carcinoembryonic antigen 294, carbohydrate antigen19-9 (CA19-9) 295, CA15-3 296, CA125 and CA15-3 297,298, desmin 299, HER2, MCF-7 300, alpha-fetoprotein 301 and mitochondria 302. These studies depicted that CDs can be used as a valuable tool for early cancer diagnosis. More significantly, the novel L-CD/C-CD prepared from Gd (iii) salts/complexes, cationic polymers and CA can monitor the gene delivery process in real-time. The experiments were performed in mice bearing mammary tumors with the clinical reagent Gd-DTPA as a control. The result revealed that the continuous effect of C-CD was better, and the enhanced signal was still clearly visible after 180 min. It also confirmed that the EPR effect can make the accumulation of MR signals in the tumor which showed the advantage of CDs in tumor diagnosis (Figure 18C) 303.

Except in the early diagnosis of human and animal diseases, some studies have reported that the research of CDs in plants showed the application prospects of CDs in the diagnosis of plant diseases. Ling and his research groups reported 304 that labeling indole propionic acid (IPA) receptors in plant tissues can further track the signal transduction process of IPA. First, the carboxyl-modified CDs were prepared by cleaving CA at high temperature, and then the IPA-modified CDs were prepared by coupling the amino group of tryptophan with the carboxyl of prepared CDs. The prepared CDs can avoid the green fluorescent background of plants, and realize the labeling and imaging of IPA binding protein in plant tissues.

### 7.2. Integration of diagnosis and treatment

**Figure 19:**
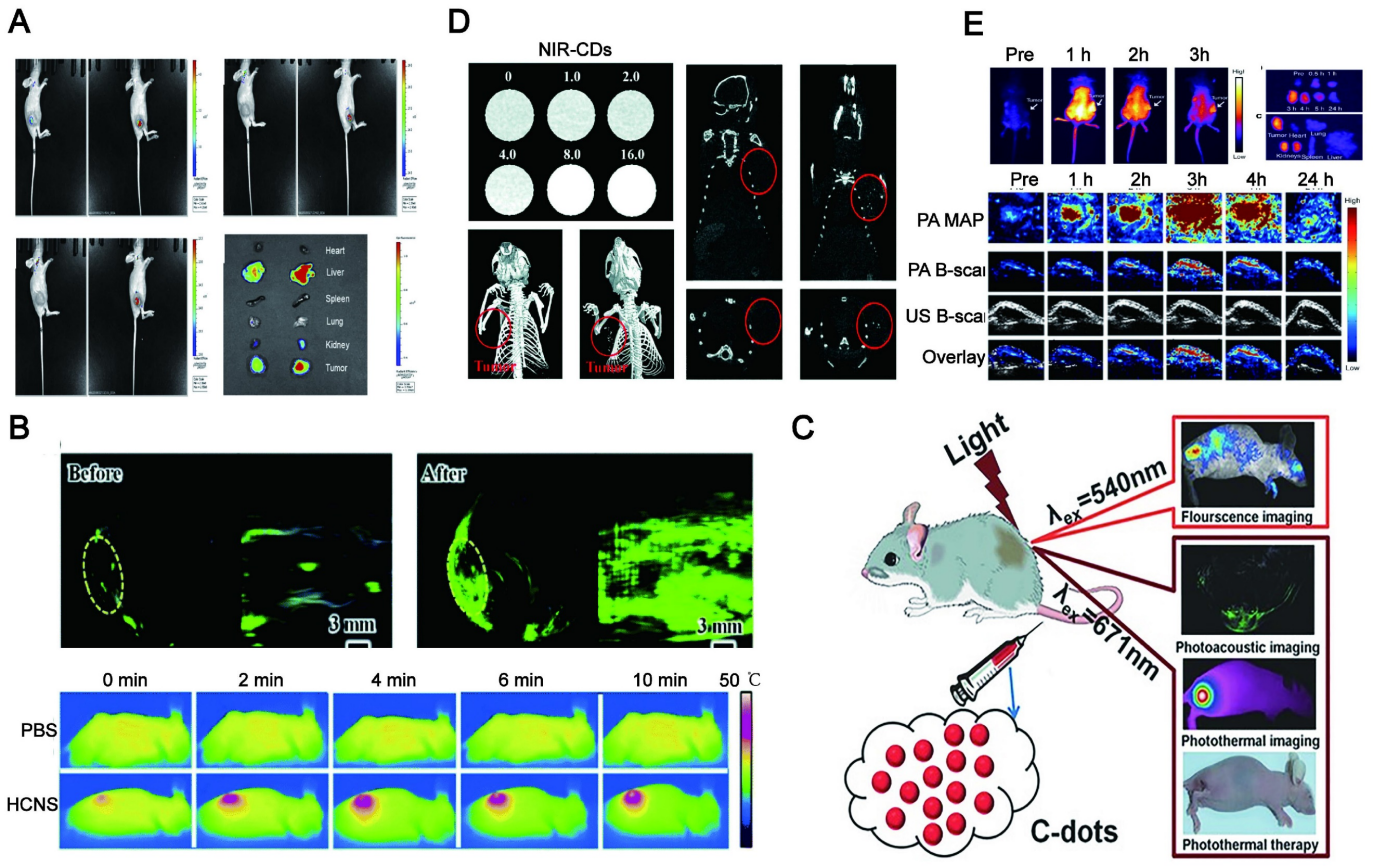
CDs as a new strategy for integrated application of diagnosis and therapy* in vivo*. (A) *In vivo* PL images of nude mice treated with an intratumor injection of CDs. Adapted with permission from 306. Copyright 2019, Elsevier. (B) Photoacoustic images and PT images of the HeLa tumor-bearing mice after the intravenously injected with CDs. Adapted with permission from 324. Copyright 2019, Royal Society of Chemistry. (C) CDs act as multifunctional fluorescent, photoacoustic, and thermal theranostics for simultaneous diagnosis and therapy of cancer. Adapted with permission from 325. Copyright 2015, John Wiley and Sons. (D) CT images of NIR-CD/MoS2 solutions with different concentrations and CT images of mice before and 24 h after i.v. injection with NIR-CD/MoS2. Adapted with permission from 328. Copyright 2019, Royal Society of Chemistry. (E) NIR fluorescence images of mouse bodies, H22 tumors and major organs after intravenous injection of CDs at various time points. Adapted with permission from 175. Copyright 2018, Springer Nature.

The combination of visualization *in vivo* and treatment strategy has become a promising method to obtain the best therapeutic effect and precision. It has obvious advantages in comparison to a single diagnosis or treatment method. Specifically, the integration of diagnosis and treatment has great potential in terms of patient stratification and personalized medicine, real-time monitoring of drug treatment processes and feedback on drug treatment effects 305. Compared with small molecules (fluorophores) and traditional semiconductors (inorganic quantum dots), LW-CDs exhibit some special advantages. It is reported that the CDs with NIR fluorescent encapsulated into liposomes can easily display the distribution of liposomes *in vivo* (Figure 19A) 306. CDs modified by cholera toxin acting as fluorescent neural tracers could be absorbed and retrograde transported by neurons in the mice's peripheral nervous system 307. Some bacterial diseases can also be treated by selecting the synthesized precursor of CDs 308. The individual CDs are commonly helpless for serious diseases such as tumors and cancer due to their lacking targeting ability without surface functional group properties (rich in carboxyl, amino and hydroxyl groups). Nevertheless, these functional groups can form a conjugated structure during CD synthesis. The drugs 309, photosensitizers (PS) 310, deoxyribonucleic acids 311, peptides and biomarkers (such as FA) 312 molecules can be loaded on the CD through the hydrophobic effect and π-π interaction. CDs doping and modification can be combined with the current therapeutic tool (PTT, PDT) since they can simultaneously act as tracers, photosensitizers and PT agents. It is not hard to find that CDs have bright prospects in the integration of diagnosis and treatment of diseases.

PTT is a promising method of cancer treatment. Ideal PTT drugs should absorb longer-wavelength radiation for the thermal ablation of cancer cells. A variety of organic and inorganic materials such as indocyanine 313, phthalocyanine 314, diketopyrrolopyrrole 315, cocaine 316, porphyrin 317, Au NPs 318, Pd NPs 319, metal sulfide 320, iron oxides 321, and Graphene quantum dots 322 have confirmed to be the potential NIR absorbers. However, they can't trace and image *in vivo.* This prevents them from monitoring treatment effects in real-time. The CDs have the dual effects of thermal ablation and trace. It was reported 323 that the cytoplasmic dissolution and nuclear deformation were observed under 980 nm laser irradiation for N-doped CDs located at the cytoplasm of cancer cells for 5 min and 10 min. It was mainly ascribed to the enhancement in intracellular temperature. As reported in many pieces of literature, this PT effect may trigger certain cellular responses that led to a transformation of plasma membrane function and activation of heat-sensitive proteins in cancer cells (Figure 19B) 324. More intriguingly, the CDs with red emission can be simultaneously used as fluorescence tracers, photoacoustic (PA) imaging and hyperthermia agents in tumor diagnosis and treatment in living mice (Figure 19C) 325. The photo-thermal conversion efficiency of the prepared CDs under 671 nm laser irradiation was as high as 38.5%. Importantly, red FL imaging can be used with excitation at 350-600 nm, corresponding to PA imaging and PT treatment at 671 nm. The NIR CDs not only have better bioimaging, but also provide more efficient PTT. The NIR-CDs prepared by a cyanine dye and PEG-800 held a high PT conversion efficiency of 38.7% and NIR fluorescent imaging *in vivo* 326. The S, Se-codoped CDs independent of the excitation wavelength showed a PT conversion efficiency as high as 58.2% and bright fluorescence imaging 327. The PTT and PCT performances of 0D/2D/0D sandwich heterojunction were further enhanced by using 0D N-doped CDs and 2D MoS_2_ nanosheets. This method further improved the NIR absorbance and thus realized a high PT conversion efficiency of 78.2% (Figure 19D) 328. Recently, a simple solvothermal method was reported to prepare the NIR CDs with high PT conversion efficiency (59%) by using CA, urea, and DMSO and visualization imaging (Figure 19E) 175.

The multi-functional CDs not only achieve integration of diagnosis and treatment, but also support more precise targeted imaging and therapy *in vivo*. The multi-functional LW-CDs can combine with specific biomarkers for targeted diagnosis and treatment. For example, both the integrin V family overexpressed in the membranes of tumor cells can be used as signals for early detection of the tumor 329. The CD-based nanocomposites modified by the ligand of integrin V can be explored as multifunctional reagents for PA imaging and PTT. The temperature of Integrin α_v_β_3_ modified CD nanocomposites was increased up to 14.1 °C under an 808 nm pulsed laser for 5 min, while the native solution did not exhibit a notable PT effect 330. Importantly, the nanocomposites could target the cell membranes of superficial malignant tumors due to the specific recognition of arginine-glycine-aspartate (RGD) ligands with integrin α_v_β_3_ receptors 331.

In addition to ligand modification, some stimuli-responsive CDs have been recently explored for targeted imaging and therapy. A functional nanocomposite system consisted of CDs embedded with a magnetic Fe_3_O_4_ core and a mesoporous silica shell was reported to be used as an imaging probe and PT agent. The nanocarriers release paclitaxel (PTX) in answer to NIR radiation due to the local heating of CDs. This responsive composite nanosystem can achieve both the chemotherapeutic effects of PTX and PDT therapeutic effects by heat-killing cancer cells 332. Similarly, a multi-stimulus response system that combined infrared thermal imaging of cells with thermochemotherapy was recently explored to target tumors 333. A pH/redox/NIR multi-stimulus response system was also constructed 334.

Multifunctional CDs can also be used in PDT for effective disease diagnosis and treatment. The fluorescent CDs (DPP CD) of diketopyrrolopyrrole could maintain the ability of DPP to produce singlet oxygen and greatly inhibit tumor cell growth under laser irradiation (540 nm), and also realize the visualization of the target site 335. Similarly, the QY of singlet oxygen in copper-doped CDs reached up to 36%, and achieved PDT and imaging at the same time 336. Hypoxia was an important feature of the tumor microenvironment (pO2 ≤ 2.5 mmHg), which greatly limited the efficacy of PDT 337. The currently reported "photosensitizer-upconversion NP" mode was considered to provide a poor solution for PDT, because of the low QY (usually less than 3%) and the severe tissue overheating caused by 980 nm laser 338. It was worth noting that the light-driven water-splitting method based on CDs had been tried to solve the problem of hypoxia at tumor sites. The mechanism for introducing CDs was to increase red region absorption to trigger the *in vivo* water-splitting process. The wide band gap of the separate water-splitting materials (eg. TiO_2_) caused the rapid recombination of photogenerated electron/hole pairs 339, which were poor to the response of infrared light. As known, element-doped CDs can increase the therapeutic effect of PDT. For instance, CDs doped by C_3_N_4_ enhanced its red region absorption and thus trigger water-splitting *in vivo* 340. Mn^2+^ doped CDs can impart strong magnetic resonance capability (r_2_/r_1_ ratio of 5.77) 341. The functional CD synthesized by different precursor molecules played an important role in PDT. Li et al. reported that porphyrin-based CDs (TPP-CD) had effective PDT activity after irradiation 342. The main reason for the gap between the two groups may be attributed to that N - H, C - N, and C - O bonds existed in TPP CDs 343.

Currently, the special properties of CDs enable the combined application of PTT and PDT as an ideal therapeutic strategy. Studies have shown that multifunctional nanocarriers fabricated by the assembly of iron oxide-modified CDs (Fe_3_O_4_-CDs) and black P quantum dots (BP-QDs) could produce the synergy of PTT and PDT 344. Interestingly, the introduction of SiO_2_ could improve the chemical stability of the nanocomposite in the physiological environment, and prevent the absolute extinction of its fluorescence 345. Another study embedded PS and PT agents in nanocarriers, reducing the risk of therapeutic drugs entering the blood circulation after intravenous injection 346. The HB-CDs prepared by using HB as biomass materials could highly generate singlet oxygen (0.38) and PT conversion efficiency (27.6%) as well as imaging under 635 nm laser irradiation 55. This enables HB-CDs a potential bifunctional delivery vehicle for synergistic PDT/PTT treatment of tumor cells. Synergistic therapy of PDT/PTT often uses different excitation sources to respond to PDT and PTT, respectively, but the problem of high-power laser irradiation for PTT has not been properly dealt with. Recently, Sun and his research team proposed a new concept for collaborative PTT/PDT. They anchored a small amount of photosensitizer Ce6 onto CDs with red fluorescence that possessed superior PT and PD characters under the same wavelength of 671 nm NIR laser 54.

It can be found that the combination of CDs with PTT and PDT treatment strategy has great application prospects. In addition, LW-CDs can significantly enhance the imaging and therapeutic effect of PTT and PDT.

### 7.3. Visualization of drug-targeted delivery

**Figure 20:**
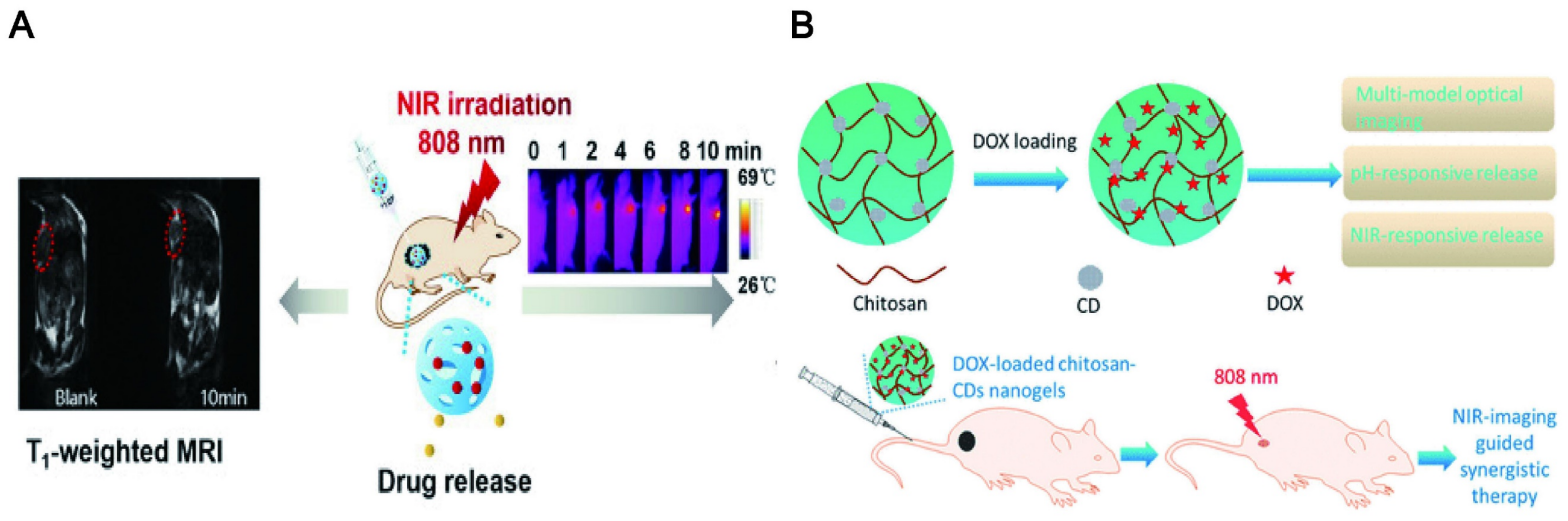
Visualization of drug-targeted delivery. (A) MRI guided PT chemotherapy of Gd@CDs. Adapted with permission from 351. Copyright 2021, Royal Society of Chemistry. (B) Schematic depiction of DOX-loaded CCHNs for therapeutic application. Adapted with permission from 352. Copyright 2017, American Chemical Society.

The CDs with a small size (<10 nm), easy surface modification, LW fluorescence emission and water-solubility have become the preferred carrier for targeting and tracking imaging *in vivo*. The CDs combined with the drug by non-covalent bonds, covalent bonds, electrostatic interaction and hydrazone bonds 347-350 can establish a stable delivery system *in vivo*. This delivery system was visualized in the lesion site and triggered the drug release through different pH in the internal environment. Jiang et al. synthesized NIR gadolinium-doped CDs using 3,4-dihydroxyhydrocinnamic acid, 2,2'-(ethylenedioxy)bis(ethylamine) and gadolinium chloride as raw materials. Gadolinium-doped CDs loaded with the chemotherapeutic drug doxorubicin hydrochloride (Dox) showed clear visualization imaging by MRI and rapid drug release in tumor sections (Figure 20A) 351. Wang and his research groups reported a chitosan-CDs hybrid nanogel for simultaneous NIR imaging and NIR/pH dual-reactive drug release for enhanced therapeutic efficacy (Figure 20B) 352. To further specifically monitor the carrier that is transported to tumor cells to release the drug, the LW-CDs with H-switchable zwitterionic surfaces were designed. It can reduce the absorption of non-specific proteins to extend the circulation time by maintaining the negative surface under neutral physiological conditions. When the pH of the tumor microenvironment was below 6.8, it was positively charged to promote the uptake of tumor cells. This is consistent with a pH-responsive antimicrobial coating mechanism, where esterases fail to trigger cargo release in normal tissues. This design guaranteed cargo target delivery into tumor cells and monitored visually 353. The conversion of surface charge via pH change can also guide the targeted release of drugs. For example, the grafting of polyanionic dimethylmaleic acid onto cisplatin-loaded LW-CDs, which converted to a positive charge in the tumor microenvironment (pH=6.8) and increased the high affinity for cancer cell membranes that facilitated efficient internalization of the prodrug cisplatin 354. Similar conclusions also emerged in Ma's research 178. They demonstrated that a significant selective nucleolar staining was presented in LN229 and Hela cell lines when LW-CDs carried positive and negative charges. By comparison, highly negatively charged LW-CDs failed for nucleolar imaging. Lately, pH/redox dual-response LW-CDs were designed to trigger the targeted delivery of anticancer drugs. The benzyl-imine bond of LW-CD was hydrolyzed in the tumor environment to expose the RGD peptide and thus demonstrated efficient uptake through the recognition interaction of RGD ligands with cancer cell receptors. After internalization by cancer cells, cisplatin prodrug-loaded LW-CDs were visualized in the cytoplasm of cancer cells, exhibiting a therapeutic effect and released drugs 355.

The use of overexpressed receptors of cancer cells can also activate composite materials to achieve visualized targeted delivery and release of drugs. Gao and his research groups 349 reported a complex fluorescent probe for targeted cancer cell imaging that was fabricated by electrostatic interaction to assemble PEI-modified CDs and hyaluronic acid-crosslinked DOX. This composite system could target and penetrate cancer cells by exploiting the high affinity of hyaluronic acid for the CD44 receptor overexpressed in tumor cells. Simultaneously, hyaluronic acid-crosslinked DOX could be cleaved into smaller fragments by activation of the high express hyaluronidase of the tumor microenvironment, which promoted the rapid release of DOX. Similar studies have been reported by using transferrin 283, FA 229, recognition of epidermal growth factor receptors 356. Overall, LW-CDs enable drug delivery to target sites. What is even more fascinating is that the LW-CDs can also realize the visual monitoring of drugs *in vivo*.

### 7.4. Visualization of gene-targeted delivery

**Figure 21:**
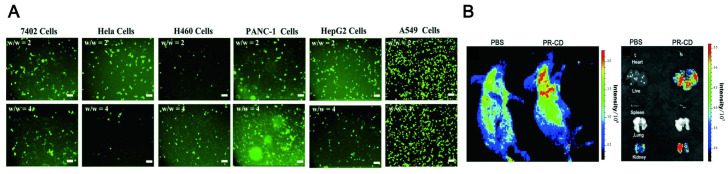
Visualization of gene-targeted delivery. (A) Fluorescence microscopy images of pEGFP-transfected A549 cells. Adapted with permission from 360. Copyright 2019, Royal Society of Chemistry. (B) Fluorescence imaging of the nude mouse treated with CDs *in vivo*. Adapted with permission from 367. Copyright 2021, Royal Society of Chemistry.

The higher transfection efficiency, tunable PL and negligible toxicity enabled CDs to conduct as imageable and trackable gene nanocarriers compared to various types of traditional nanoplatforms, for example, polymeric nanomaterials 192, precious metal NPs 357, carbon nanostructures 358, and biological nanomaterials 359. Studies have shown that CDs derived from cationic polymers can enhance gene delivery and cell imaging (Figure 21A) 360. This mechanism was that the structure of the nucleic acid material was composed of negatively charged phosphate groups, and the cationic polymer combined with the nucleic acid into particles with a positively charged structure through electrostatic interaction. The interaction of these positively charged particles with negatively charged components (including proteoglycans) on the cell membrane resulted in adsorptive endocytosis to form endosomes 361. The synthesis of cationic CDs can be modified by different precursors or surface passivators, for example, positively charged polymers with amines including polyethylenimine 362, chitosan 363, ethylenediamine 364, and poly(amido amine) 365. Higher transfection efficiency promotes the application of CDs in gene delivery. Construction of amphiphilic CDs (ACDs) by coupling hydrophobic alkyl epoxides to the amino groups of PEI-modified CDs surface enabled ACD to have higher transfection efficiency than lipid-like substances. More importantly, ACD achieved a multi-functional carrier that could effectively transfect survivin siRNA toward A549 cells and visualized the efficient uptake by cells for slow release of DOX 366. Positively charged CD synthesized by using PEI and FA can be used for the selective imaging of FA receptors in biological systems. The positively charged cationic PEI can link them to plasmid DNA and effectively transfect therapeutic plasmids into cells 286.

Interestingly, PEI was extensively studied as a cationic polymer gene carrier, that combined with LW-CDs can achieve high transfection efficiency and visualized delivery to target sites. Chen et al. synthesized LW-CDs using rhodamine and PEI, which was 162-fold more transfection efficiency than the "gold standard" transfection reagent PEI 25 kDa. Meanwhile, the CD can also be used for intracellular tracking of the gene delivery process (Figure 21B) 367. However, a comparison of CDs/pSOX9 NPs and PEI-CDs/pDNA complexes found that the former had, even more, higher transfection efficiency. It was attributed to the rupture of the cell membrane by PEI, resulting in cell necrosis and fragmentation of the mitochondrial membrane, which eventually led to the apoptosis of the cells. Compared with PEI, cationic and small-sized CDs enhanced the attachment of pDNA to cell membranes without any cellular damage 368. Therefore, ethylenediamine 369 and spermine 370 instead of amine compounds were introduced into the preparation process of LW-CDs for surface passivation. In conclusion, LW-CDs as gene carriers are an efficient and feasible strategy that can visualize the delivery process and achieve precise target site delivery of genes.

## 8. Conclusion and future perspectives

LW-CDs with smaller sizes and carbon-based materials often have excellent biocompatibility and safety after entering the body. They can penetrate the cell membrane and enter the nucleus to effectively carry drugs or genes. The doped CDs will not cause fluorescence decline to transfer *in vivo*, which is of great significance for real-time monitoring *in vivo*. Special optical properties (stable and regulable) enable CDs more suitable *in vivo* as visualization materials. It is worth noting that the functional groups on the surface of the CDs can be grafted with other groups by covalent, non-covalent or electrostatically adsorbed to achieve targeting delivery and combine PTT and PDT to show significant prospects in the visualization treatment of diseases. Although, emitting LW-CDs applied *in vivo* have evolved from a single carrier to a functional composite nanosystem, it needs to overcome more difficulties to realize the practical applications of CDs *in vivo*.

First, a large number of precursors and synthesis methods have been studied to prepare CDs, but only a small amount of prepared CDs can achieve LW emission. Another, although the degree of surface oxidation, heteroatom doping, and isolated sp^2^ domains of particle size is developed to explain the mechanism of LW emission, more instructive theories and methods should be established to fine-tune the properties of CDs such as high QY and photostability. Most basically but importantly, CDs show excellent safety than other nanomaterials. However, studies have reported that a certain concentration of CDs will result in a decrease in cell viability. The concentration-dependent toxicity of CDs is closely related to the synthetic precursors. For example, CDs prepared by employing puerariaelobatae radix, and adenosine 5′-triphosphate as precursors have certain toxicity and synthesized by CA show almost no toxicity. The toxicity mechanism of CDs needs further exploration.

Secondly, various properties of CDs reveal their huge application prospects *in vivo*, but there are still few reports about CDs on their diagnosis and treatment effects against diseases *in vivo*. This main reason can be divided into several aspects: (1) Although CDs emitting LW into the body can avoid background fluorescence interference, the fluorescence properties are susceptible to interference from the complex internal environment of the body (such as pH, enzyme), thereby the short fluorescence lifetime cannot achieve diagnostic and therapeutic effects. Recent research found that NIR-phosphorescent CD with long-lived triplet states can significantly enhance sonodynamic therapy 371. (2) The combined application of CDs and new medical strategies such as PTT and PDT has been reported to achieve a certain synergistic effect *in vitro*, while the relationship between the demand fluorescence emission intensity of CDs *in vivo* for therapeutic effects and safe fluorescence intensity on the normal cells was still not clear. (3) The grafting of CDs with biomarkers can achieve targeting, the relationship between the number of CDs that reach the site and the efflux threshold of the body's immune system needs to be further explored.

Thirdly, the delivery of drugs or genes by CDs is often encapsulated inside, but there are few studies on whether the encapsulation of CDs is stable *in vivo*, such as whether it leaks or the number of leaks before reaching the target site. In addition, the current encapsulation rate is still low. The synthesis method and precursor selection are needed to further explore. Simultaneously, the influence of the size, shape, surface charge and functional groups on the target delivery performance *in vivo* should be more clarified.

It can be concluded that the following strategies are needed to promote the application of LW-CDs in the future: (1) The fluorescence properties of emitting LW-CDs such as fluorescence intensity, fluorescence stability, QY and its influencing factors (solvent, pH) should be further clarified. (2) It is urgent to understand the changes in CDs in the microenvironment to prepare the best CDs. (3) The photo-thermal conversion efficiency and ability to generate singlet oxygen are closely related to the choice of precursors. Understanding the mechanism that generates high efficiency and ability is conducive to maximizing the synergy between CDs and PTT or PDT. (4) The drug encapsulation capacity of CDs should be improved to achieve more efficient drug and gene delivery. As the above-mentioned key issues are solved, the LW-CDs will show bright prospects for targeted drug delivery, diagnostic imaging, and visual therapy to improve human healthcare.

## Figures and Tables

**Table 1:** Merits and defects of various *in vivo* imaging agents

		Merits	Applications	Defects	References
Fluorescent proteins		1. High sensitivity 2. Non-toxic	1. Detection and tracking	1. Prone to transduction failure 2. Darker color	48,49
Fluorescent dyes	Coumarins	1. High QY 2. Large Stokes shift 3. Good light stability 4. Easy modification	1. Detection of various ions and active substances 2. Fluorescent labeling of nucleic acid and protein molecules	1. Short emission wave 2. Easy to be interfered with background fluorescence	50,51
	Rhodamines	1. High QY and molar extinction coefficient 2. Excellent Water solubility	1. Single particle tracking 2. Super-resolution imaging 3. Detection	Absorption and emission wavelengths are only in the visible region	52,53
	Anthocyanins	1. NIR fluorescence 2. Good biocompatibility 3. Low toxicity	1. Tumor targeting 2. Imaging *in vivo* 3. Biosensing	1. Poor light stability 2. Prone to photobleaching 3. Low fluorescence QY 4. Low solubility in water	54,55
Bioluminescence	Luciferase	1. Strong specificity 2. High sensitivity 3. Accurate quantification 4. Non-toxic 5. Easy to penetrate membranes barrier	1. Markers of multiple enzyme genes 2. Tracer stem cells	1. Requires an external source to give the substrate to emit light 2. Only two-dimensional images	56
Nanomaterials	Long afterglow NPs	1. No excitation 2. Avoid background interference 4. Strong tissue penetration ability 3. No toxic	1. Biosensing detection 2. Imaging *in vivo* 3. Drug delivery and treatment	1. Difficult to prepare for NIR materials 2. Difficult to modify 3. Poorly water-soluble	57
	Semiconductor quantum dots	1. Adjustable size 2. High yield 3. Good light stability 4. Easy modification	1. Imaging 2. Sensor photovoltaic	1. Metal ions 2. Toxic 3. Insoluble in water	58
	Upconversion NPs	1. High chemical stability 2. Light bleaching resistance 3. Good water solubility, Low toxicity 4. Long fluorescence lifetime	1. Bioimaging 2. Biodetection, 3. PDT 4. Drug delivery	1. Low luminous efficiency 2. Large size	59
	Magnetic NPs	1. Unique magnetic properties 2. Good biocompatibility 3. Large specific surface area 4. Easy to modify	1. Magnetic resonance imaging 2. Hyperthermia 3. Targeted drug delivery	1. Prone to reunion 2. Low use efficiency	60
	RNA aptamer	1. No background interference 2. Simple preparation	1. Cell RNA imaging	1. Highly dependent on non-physiological ion concentration 2. Low light stability	61,62
Isotopes	Radionuclide	1. Real-time non-invasive tracing 2. Targeting various biological molecules *in vivo*	1. Stem cell therapy 2. Tumor cell labeling and therapy 3. Cellular immunity research	1. Low spatial resolution 2. Radiation generation 3. Expensive detection equipment	63,64

**Table 2:** The strategy for improving the QY of CDs

	Materials	QY (%)	Mechanism	References
Doping	Urea and thiourea	78	Electron transition from the ground state to the lowest excited singlet state	74
	NH_3_ and boric acid	43.2		78
	Si	97.32		80
	Cr	20		81
	Phosphoric and hydrochloric acids	15		82
	Zn	32.3		83
	Boric acid	25		84
	Se	52		85
Hydrogen bonding	Ethanol solution	50.3	The stabilization of the excited state for more efficient radiative recombination	86
	PVA solution	47		87
High crystalline state	Catechol	32	Reduces the non-radiative electron-hole recombination center	89
	Ethanol	38.7		90
Heteroatom-precursor	CA	53		91
	FA	94.5		92
	Ethylenediamine	73		93
	L-cysteine	78		97
	PEI	54		95
	β-alanine	14		96
	1,2,4-triaminobenzene	50.3		97
	Diethylenetriamine	27.7		98
	Ethanolamine	21.85		99
	Ammonium citrate	67		100

**Table 3:** The strategy for improving the photostability of CDs

	CDs	Precursor	Performances	References
Hydrophily	N-CDs	P-phenylenediamine (p-PD) and ethanol	19 times of continual irradiation (6 min at every turn)	109
	N-CDs	CA and m‐aminophenol	Continuous laser irradiation for 30 min	110
Co-doped	N, P-CDs	Malic acid, ethylenediamine and phosphoric acid	60 min exposure under 350 nm UV	112
	F,N-CDs	Poly(ethylene imine) and levofloxacin	Stablity at room temperature for three months	114
	B,N-CDs	Cresyl violet and boric acid	Continuous ultraviolet light irradiation for 20 h	115
	S,N-CDs	Allium sativum peels	Continuous exposure to UV light for 60 min	116
	N,Cl-CDs	l-Ornithine hydrochloride	Being stored 1 year at ambient temperature	117
	P,Cl-CDs	Phosphoric and hydrochloric acids and maltose	Continuous excitation at 390 nm for 100 min	93
Passivation	PEG-CDs	PEG_6000_, pyrene and nitric acid	Photostability after 30 s of irradiation using a laser for 557 nm	119-120
	PEI-CDs	Glycerol and branched PEI25k	Stable in the pH range of 5-12	121-122
	PVA-CDs	Polyvinyl alcohol and waste tea residue	Stability under the UV light	123
	PVP-CDs	Poly(vinylpyrrolidone) and L-Cysteine	Maintain stability for 1 h under continuous UV light (365 nm) illumination	124

**Table 4:** The natural raw materials for synthesizing CDs

Precursor	Excitation wavelength (nm)	Emission wavelength (nm)	References
Bagasse	372	627	162
Carica papaya juice	Excitation-dependent	Multicolor	163
Lemon juice	Excitation-dependent	Multicolor	164
Orange peels	460	590	162
Curcumin	Excitation-dependent	Multicolor	166
Orange waste peel	Excitation-dependent	Multicolor	167
Black tea	490	590	168
Bee Pollens	Excitation-dependent	Multicolor	169
Cabbage	Excitation-dependent	Multicolor	170
Onion waste	Excitation-dependent	Multicolor	171
Osmium sanctum	Excitation-dependent	Multicolor	172
Manilkara zapota	Excitation-dependent	Multicolor	173

**Table 5:** Metabolic pathways of CDs

Metabolic pathway	Precursor	Excitation wavelength (nm)	Emission wavelength (nm)	References
Kidney	CA and urea	Excitation-dependent	Multicolor	175
Kidney	Glycine	Excitation-dependent	Multicolor	176
Kidney	Atlantic salmon	Excitation-dependent	Multicolor	177
Kidney	Dry taxus leaves	350-680	670 and 720	178
Kidney	Glucose and ethylenediamine	Excitation-dependent	Multicolor	179
Kidney	Gadopentetic acid	Excitation-dependent	Multicolor	180
Kidney	Diamine-terminated oligomeric poly	420 and 700	520 and 800	171
Liver	Aqueous beetroot	Excitation-dependent	Multicolor	172
Liver	O-Phenylenediamine	Excitation-independent	630	183
Liver	Watermelon juice	808	925	36

**Table 6:** A summary of syntheses and FL properties of CDs with red/NIR

	Carbon resource	N resource or others	Synthetic approach	Excitation wavelength (nm)	Emission wavelength (nm)	Mechanism	References
Top-Down	Tire soot	Nitric acid	Oxidation	430-710	500-800	Surface defects	218
	Carbon fibers	Nitric acid	Oxidation	Excitation‐independent	430-610	Size and degree of surface oxidation	219
	Coal pitch powder	Formic acid and H_2_O_2_	Oxidation	Excitation‐independent	630	Intermolecular forces	220
	Graphite	K_2_S_2_O_8_ solution	Electrochemical	Excitation‐independent	610	Conjugated sp^2^	221
	Alcohols		Electrochemical	390-410	485-505	Conjugated sp^2^	222
	Toluene		Laser ablation	Excitation‐independent	600	Quantum confinement effect	223
	Graphite Powders		Laser ablation	400	650	Size	224
	Graphite	NH_2_	Ultrasonic	543	580-750	Surface defects	225
Bottom-Up	P-phenylenediamine	Phosphorus acid	Hydrothermally	Excitation‐independent	622	Surface Molecular Fluorescence	226
	CA	Neutral red	Hydrothermally	Excitation‐independent	632	Compositions and structures	163
	P-phenylenediamine	Ni^2+^	Hydrothermally	Excitation‐independent	535-700	Size and degree of surface oxidation	50
	Trimesic acid	4-aminoacetanilide	Hydrothermally	520	582	Size and emissive trap sites	227
	2,5-diaminobenzenesulfonic acid		Hydrothermally	Excitation‐independent	593	Size and surface defects	228
	FA	Phenylenediamine isomers	Hydrothermally	365	530, 429, and 612	Surface state	229
	3-aminobenzeneboronic acid	2,5-diaminobenzenesulfonic acid	Hydrothermally	520	605	Size and surface state	230
	Cresyl violet	Boric acid	Hydrothermally	520	616	Surface state	139
	CA	Ethanediamine and lignin	Hydrothermally	375-460	454-535	Aromatic π systems and n - p* transitions	231
	O‐phenylenediamine	Dopamine and HNO3	Hydrothermally	Excitation‐independent	630	Conjugated aromatic π systems and hydrogen bonds	226
	P‐phenylenediamine	O‐phenylenediamine and dopamine	Hydrothermally	573	640	Conjugated sp^2^	232
	CA	Urea and N,N-dimethylformamide	Hydrothermally	553	606	n-π* transition of heteroatomic	233
	P-phenylenediamine	Urea	Hydrothermally	Excitation‐independent	440-625	Degree of oxidation	234
	CA	5-amino-1,10-phenanthroline	Hydrothermally	560	630	Phenanthroline part	235
	CA	1,4,5,8-tetraminoanthraquinone	Hydrothermally	600	700	C=C and C=N	236
	L‐methionine	urea	Hydrothermally	550	625	Conjugated sp^2^	237
	Selenourea	O-phenylenediamine and hydrochloric acid	Hydrothermally	Excitation‐independent	625 and 679	Low-energy surface state	238
	CA	Ethanediamine and meso-tetra (4-carboxyphenyl) porphin	Hydrothermally	Excitation‐independent	645	The surface structures and states	239
	1,3-dihydroxynaphthalene	KIO_4_	Solvothermal	530	628	Conjugated sp^2^	240
	Perylene	HNO3 and NaOH	Solvothermal	560	610	Surface electronic state induced by the alkali	241
	Taxus leaves	Acetone	Solvothermal	413	722	Excited state relaxation channels and single PL center	219
	4-aminophenol	KIO_4_ and ethanol	Solvothermal	540	620	C=C and C=N	242
	CA	PEI and formamide	Solvothermal	550	640	Surface state	54
	Alizarin	Urea	Microwave-assisted	Excitation‐independent	605	C=C and C=N	243
	Glutathione	Formamide	Microwave-assisted	420	683	Surface molecular state	244

## References

[1] Smith BR, Gambhir SS (2017). Nanomaterials for *in vivo* imaging. Chem Rev.

[2] Tallury P, Malhotra A, Byrne LM, Santra S (2010). Nanobioimaging and sensing of infectious diseases. Adv Drug Deliv Rev.

[3] Kircher MF, Mahmood U, King RS, Weissleder R, Josephson L (2003). A multimodal nanoparticle for preoperative magnetic resonance imaging and intraoperative optical brain tumor delineation. Cancer Res.

[4] Yang Y, Wang L, Wan B, Gu Y, Li X (2019). Optically active nanomaterials for bioimaging and targeted therapy. Front Bioeng Biotechnol.

[5] Aung W, Hasegawa S, Koshikawa-Yano M, Obata T, Ikehira H, Furukawa T (2009). Visualization of *in vivo* electroporation-mediated transgene expression in experimental tumors by optical and magnetic resonance imaging. Gene Ther.

[6] Lyngbaek S, Ripa RS, Haack-Sorensen M, Cortsen A, Kragh L, Andersen CB (2010). Serial *in vivo* imaging of the porcine heart after percutaneous, intramyocardially injected 111In-labeled human mesenchymal stromal cells. Int J Cardiovasc Imaging.

[7] Etrych T, Lucas H, Janouskova O, Chytil P, Mueller T, Mader K (2016). Fluorescence optical imaging in anticancer drug delivery. J Control Release.

[8] Kang HG, Yamamoto S, Takyu S, Nishikido F, Mohammadi A, Horita R (2019). Optical imaging for the characterization of radioactive carbon and oxygen ion beams. Phys Med Biol.

[9] Ghassaban K, Liu S, Jiang C, Haacke EM (2019). Quantifying iron content in magnetic resonance imaging. Neuroimage.

[10] Gianolio E, Arena F, Di Gregorio E, Pagliarin R, Delbianco M, Baio G (2015). MEMRI and tumors: a method for the evaluation of the contribution of Mn(II) ions in the extracellular compartment. NMR Biomed.

[11] Kostelnik TI, Orvig C (2019). Radioactive main group and rare earth metals for imaging and therapy. Chem Rev.

[12] Constantinides C, Maguire M, McNeill E, Carnicer R, Swider E, Srinivas M (2018). Fast, quantitative, murine cardiac 19F MRI/MRS of PFCE-labeled progenitor stem cells and macrophages at 9.4T. PLoS One.

[13] Bengtsson NE, Brown G, Scott EW, Walter GA (2010). LacZ as a genetic reporter for real-time MRI. Magn Reson Med.

[14] Jovanovic Z, Krstic D, Nikezic D, Ros JMG, Ferrari P (2018). Mcnpx calculations of specific absorbed fractions in some organs of the human body due to application of 133xe, 99mtc and 81mkr radionuclides. Radiat Prot Dosimetry.

[15] Blaire T, Bailliez A, Ben Bouallegue F, Bellevre D, Agostini D, Manrique A (2018). First assessment of simultaneous dual isotope (123I/99mTc) cardiac SPECT on two different CZT cameras: A phantom study. J Nucl Cardiol.

[16] Follacchio GA, De Feo MS, De Vincentis G, Monteleone F, Liberatore M (2018). Radiopharmaceuticals labelled with copper radionuclides: clinical results in human beings. Curr Radiopharm.

[17] Lodi F, Malizia C, Castellucci P, Cicoria G, Fanti S, Boschi S (2012). Synthesis of oncological [11C]radiopharmaceuticals for clinical PET. Nucl Med Biol.

[18] Wunder A, Klohs J, Dirnagl U (2009). Non-invasive visualization of CNS inflammation with nuclear and optical imaging. Neuroscience.

[19] Claudon M, Guillaume L (2015). Diagnostic imaging and radiation hazards. Rev Prat.

[20] Dunger D, Krause M, Grafe D, Merkenschlager A, Roth C, Sorge I (2018). Do we need gadolinium-based contrast medium for brain magnetic resonance imaging in children?. Pediatr Radiol.

[21] Boakye-Yiadom KO, Kesse S, Opoku-Damoah Y, Filli MS, Aquib M, Joelle MMB (2019). Carbon dots: Applications in bioimaging and theranostics. Int J Pharm.

[22] Remington SJ (2011). Green fluorescent protein: a perspective. Protein Sci.

[23] Egloff-Juras C, Bezdetnaya L, Dolivet G, Lassalle HP (2019). NIR fluorescence-guided tumor surgery: new strategies for the use of indocyanine green. Int J Nanomedicine.

[24] Wei S, Yin X, Li H, Du X, Zhang L, Yang Q (2020). Multi-color fluorescent carbon dots: graphitized sp2 conjugated domains and surface state energy level co-modulate band gap rather than size effects. Chemistry.

[25] Wang X, Cao L, Yang ST, Lu F, Meziani MJ, Tian L (2010). Bandgap-like strong fluorescence in functionalized carbon nanoparticles. Angew Chem Int Ed Engl.

[26] Liu J, Kong T, Xiong HM (2022). Mulberry-leaves-derived red-emissive carbon dots for feeding silkworms to produce brightly fluorescent silk. Adv Mater.

[27] Xu Y, Wang B, Zhang M, Zhang J, Li Y, Jia P (2022). Carbon dots as a potential therapeutic agent for the treatment of cancer-related anemia. Adv Mater.

[28] Hu C, Li M, Qiu J, Sun YP (2019). Design and fabrication of carbon dots for energy conversion and storage. Chem Soc Rev.

[29] Chen Y, Sun X, Wang X, Pan W, Yu G, Wang J (2020). Carbon dots with red emission for bioimaging of fungal cells and detecting Hg2+ and ziram in aqueous solution. Spectrochim Acta A Mol Biomol Spectrosc.

[30] Hua XW, Bao YW, Zeng J, Wu FG (2019). Nucleolus-targeted red emissive carbon dots with polarity-sensitive and excitation-independent fluorescence emission: high-resolution cell imaging and *in vivo* tracking. ACS Appl Mater Interfaces.

[31] Han G, Zhao J, Zhang R, Tian X, Liu Z, Wang A (2019). Membrane-penetrating carbon quantum dots for imaging nucleic acid structures in live organisms. Angew Chem Int Ed Engl.

[32] Lesani P, Singh G, Viray CM (2020). Two-photon dual-emissive carbon dot-based probe: deep-tissue imaging and ultrasensitive sensing of intracellular ferric ions. ACS Appl Mater Interfaces.

[33] Hua XW, Bao YW, Zeng J, Wu FG (2019). Nucleolus-targeted red emissive carbon dots with polarity-sensitive and excitation-independent fluorescence emission: high-resolution cell imaging and *in vivo* tracking. ACS Appl Mater Interfaces.

[34] Li Y, Bai G, Zeng S, Hao J (2019). Theranostic carbon dots with innovative nir-ii emission for *in vivo* renal-excreted optical imaging and photothermal therapy. ACS Appl Mater Interfaces.

[35] Sun S, Chen J, Jiang K, Tang Z, Wang Y, Li Z (2019). Ce6-modified carbon dots for multimodal-imaging-guided and single-nir-laser-triggered photothermal/photodynamic synergistic cancer therapy by reduced irradiation power. ACS Appl Mater Interfaces.

[36] Jia Q, Zheng X, Ge J, Liu W, Ren H, Chen S (2018). Synthesis of carbon dots from Hypocrella bambusae for bimodel fluorescence/photoacoustic imaging-guided synergistic photodynamic/photothermal therapy of cancer. J Colloid Interface Sci.

[37] Ren W, Nan F, Li S, Yang S, Ge J, Zhao Z (2021). Red emissive carbon dots prepared from polymers as an efficient nanocarrier for coptisine delivery *in vivo* and *in vitro*. ChemMedChem.

[38] Li R, Wei F, Wu X, Zhou P, Hu Q (2021). PEI modified orange emissive carbon dots with excitation-independent fluorescence emission for cellular imaging and siRNA delivery. Carbon.

[39] Vilela D, Cossio U, Parmar J, Martinez-Villacorta AM, Gomez-Vallejo V, Llop J (2018). Medical imaging for the tracking of micromotors. ACS Nano.

[40] An F, Chen N, Conlon WJ, Hachey JS, Xin J, Aras O (2020). Small ultra-red fluorescent protein nanoparticles as exogenous probes for noninvasive tumor imaging *in vivo*. Int J Biol Macromol.

[41] Lazaro-Ibanez E, Faruqu FN, Saleh AF, Silva AM, Tzu-Wen Wang J, Rak J (2021). Selection of fluorescent, bioluminescent, and radioactive tracers to accurately reflect extracellular vesicle biodistribution *in vivo*. ACS Nano.

[42] Jin T, Huang C, Cui M, Yang Y, Wang Z, Zhu W (2020). Supramolecular ensembles modified by near-infrared dyes and their biological applications. J Mater Chem B.

[43] Burbelo PD, Riedo FX, Morishima C, Rawlings S, Smith D, Das S (2020). Sensitivity in detection of antibodies to nucleocapsid and spike proteins of severe acute respiratory syndrome coronavirus 2 in patients with coronavirus disease 2019. J Infect Dis.

[44] Zheng S, Shi J, Fu X, Wang C, Sun X, Chen C Nanoscale X-ray recharged long afterglow luminescent nanoparticles MgGeO3:Mn2+,Yb3+,Li+ in the first and second biological windows for long-term bioimaging. 2020; 12: 14037-14046.

[45] Martynenko IV, Litvin AP, Purcell-Milton F, Baranov AV, Fedorov AV, Gun'ko YK (2017). Application of semiconductor quantum dots in bioimaging and biosensing. J Mater Chem B.

[46] Li H, Wang X, Huang D, Chen G (2020). Recent advances of lanthanide-doped upconversion nanoparticles for biological applications. Nanotechnology.

[47] Liu X, Wang H, Zuo S, Zhang T, Dong Y, Li D (2020). Dispersible and manipulable magnetic L10-FePt nanoparticles. Nanoscale.

[48] Karasev MM, Stepanenko OV, Rumyantsev KA, Turoverov KK, Verkhusha VV (2019). Near-infrared fluorescent proteins and their applications. Biochemistry (Mosc).

[49] Truong L, Ferre-D'Amare AR (2019). From fluorescent proteins to fluorogenic RNAs: Tools for imaging cellular macromolecules. Protein Sci.

[50] Qian Y, Zhang J, Zou J, Wang X, Meng X, Liu H (2022). NIR-II responsive PEGylated nickel nanoclusters for photothermal enhanced chemodynamic synergistic oncotherapy. Theranostics.

[51] Pramod AG, Nadaf YF, Renuka CG (2019). Synthesis, photophysical, quantum chemical investigation, linear and non-linear optical properties of coumarin derivative: Optoelectronic and optical limiting application. Spectrochim Acta A Mol Biomol Spectrosc.

[52] Liu W, Wang Y, Wu N, Feng W, Li Z, Wei L (2020). A mitochondrion-targeting fluorescent probe for hypochlorite anion in living cells. Spectrochim Acta A Mol Biomol Spectrosc.

[53] Grzybowski M, Taki M, Kajiwara K, Yamaguchi S (2020). Effects of amino group substitution on the photophysical properties and stability of near-infrared fluorescent p-rhodamines. Chemistry.

[54] Xu C, Wang Y, Yu H, Tian H, Chen X (2018). Multifunctional theranostic nanoparticles derived from fruit-extracted anthocyanins with dynamic disassembly and elimination abilities. ACS Nano.

[55] Guo C, Sun J, Dong J, Cai W, Zhao X, Song B (2021). A natural anthocyanin-based multifunctional theranostic agent for dual-modal imaging and photothermal anti-tumor therapy. J Mater Chem B.

[56] Dart DA, Bevan CL (2016). *In vivo* imaging of nuclear receptor transcriptional activity. Methods Mol Biol.

[57] Abdukayum A, Chen JT, Zhao Q, Yan XP (2013). Functional near infrared-emitting Cr3+/Pr3+ co-doped zinc gallogermanate persistent luminescent nanoparticles with superlong afterglow for *in vivo* targeted bioimaging. J Am Chem Soc.

[58] Lv Z, Wang Y, Chen J, Wang J, Zhou Y, Han ST (2020). Semiconductor quantum dots for memories and neuromorphic computing systems. Chem Rev.

[59] Ge X, Dong L, Sun L, Song Z, Wei R, Shi L (2015). New nanoplatforms based on UCNPs linking with polyhedral oligomeric silsesquioxane (POSS) for multimodal bioimaging. Nanoscale.

[60] Pastucha M, Farka Z, Lacina K, Mikusova Z, Skladal P (2019). Magnetic nanoparticles for smart electrochemical immunoassays: a review on recent developments. Mikrochim Acta.

[61] Duchardt-Ferner E, Juen M, Bourgeois B, Madl T, Kreutz C, Ohlenschlager O (2020). Structure of an RNA aptamer in complex with the fluorophore tetramethylrhodamine. Nucleic Acids Res.

[62] Kumar Kulabhusan P, Hussain B, Yuce M (2020). Current perspectives on aptamers as diagnostic tools and therapeutic agents. Pharmaceutics.

[63] Wu S, Helal-Neto E, Matos A, Jafari A, Kozempel J, Silva YJA (2020). Radioactive polymeric nanoparticles for biomedical application. Drug Deliv.

[64] Navalkissoor S, Grossman A (2019). Targeted alpha particle therapy for neuroendocrine tumours: the next generation of peptide receptor radionuclide therapy. Neuroendocrinology.

[65] Hong G, Antaris AL, Dai H (2017). Near-infrared fluorophores for biomedical imaging. Nat Biomed Eng.

[66] Hong G, Diao S, Antaris AL, Dai H (2015). Carbon nanomaterials for biological imaging and nanomedicinal therapy. chem rev.

[67] Zhang H, Wang G, Zhang Z, Lei JH, Liu TM, Xing G (2022). One step synthesis of efficient red emissive carbon dots and their bovine serum albumin composites with enhanced multi-photon fluorescence for *in vivo* bioimaging. Light Sci Appl.

[68] Liu Y, Lei JH, Wang G, Zhang Z, Wu J, Zhang B (2022). Toward strong near-infrared absorption/emission from carbon dots in aqueous media through solvothermal fusion of large conjugated perylene derivatives with post-surface engineering. Adv Sci (Weinh).

[69] Del Rosal B, Villa I, Jaque D, Sanz-Rodríguez F (2016). *In vivo* autofluorescence in the biological windows: the role of pigmentation. J Biophotonics.

[70] Han Y, Liu H, Fan M, Gao S, Fan D, Wang Z (2022). Near-infrared-II photothermal ultra-small carbon dots promoting anticancer efficiency by enhancing tumor penetration. J Colloid Interface Sci.

[71] Li Y, Bai G, Zeng S, Hao J (2019). Theranostic carbon dots with innovative nir-ii emission for *in vivo* renal-excreted optical imaging and photothermal therapy. ACS Appl Mater Interfaces.

[72] Li D, Jing P, Sun L, An Y, Shan X, Lu X (2018). Near-infrared excitation/emission and multiphoton-induced fluorescence of carbon dots. Adv Mater.

[73] Liu H, Wang X, Wang H, Nie R (2019). Synthesis and biomedical applications of graphitic carbon nitride quantum dots. J Mater Chem B.

[74] Qu D, Zheng M, Du P, Zhou Y, Zhang L, Li D (2013). Highly luminescent S, N co-doped graphene quantum dots with broad visible absorption bands for visible light photocatalysts. Nanoscale.

[75] Chen YJ, Liu ZE, Yang Q, Wang CF, Zhuo KL (2019). Nitrogen-doped highly photoluminescent carbon dots derived from citric acid and guanidine carbonate. J Nanosci Nanotechnol.

[76] Dong Y, Pang H, Yang HB, Guo C, Shao J, Chi Y (2013). Carbon-based dots co-doped with nitrogen and sulfur for high quantum yield and excitation-independent emission. Angew Chem Int Ed Engl.

[77] Shangguan J, Huang J, He D, He X, Wang K, Ye R (2017). Highly Fe3+-selective fluorescent nanoprobe based on ultrabright n/p codoped carbon dots and its application in biological samples. Anal Chem.

[78] Fei H, Ye R, Ye G, Gong Y, Peng Z, Fan X (2014). Boron- and nitrogen-doped graphene quantum dots/graphene hybrid nanoplatelets as efficient electrocatalysts for oxygen reduction. ACS Nano.

[79] Li F, Li T, Sun C, Xia J, Jiao Y, Xu H (2017). Selenium-doped carbon quantum dots for free-radical scavenging. Angew Chem Int Ed Engl.

[80] Xie Y, Zheng J, Wang Y, Wang J, Yang Y, Liu X (2019). One-step hydrothermal synthesis of fluorescence carbon quantum dots with high product yield and quantum yield. Nanotechnology.

[81] Li C, Zheng Y, Ding H, Jiang H, Wang X (2019). Chromium(III)-doped carbon dots: fluorometric detection of p-nitrophenol via inner filter effect quenching. Mikrochim Acta.

[82] Wang W, Peng J, Li F, Su B, Chen X, Chen X (2018). Phosphorus and chlorine co-doped carbon dots with strong photoluminescence as a fluorescent probe for ferric ions. Mikrochim Acta.

[83] Xu Q, Liu Y, Su R, Cai L, Li B, Zhang Y (2016). Highly fluorescent Zn-doped carbon dots as Fenton reaction-based bio-sensors: an integrative experimental-theoretical consideration. Nanoscale.

[84] Pal A, Ahmad K, Dutta D, Chattopadhyay A (2019). Boron doped carbon dots with unusually high photoluminescence quantum yield for ratiometric intracellular pH sensing. Chemphyschem.

[85] Qu D, Miao X, Wang X, Nie C, Li Y, Luo L (2017). Se & N co-doped carbon dots for high-performance fluorescence imaging agent of angiography. J Mater Chem B.

[86] Khan WU, Wang D, Wang Y (2018). Highly green emissive nitrogen-doped carbon dots with excellent thermal stability for bioimaging and solid-state LED. Inorg Chem.

[87] Abdullah Issa M, Z ZA, Sobri S, Rashid S, Adzir Mahdi M, Azowa Ibrahim N (2019). Facile synthesis of nitrogen-doped carbon dots from lignocellulosic waste. Nanomaterials (Basel).

[88] Liu C, Bao L, Yang M, Zhang S, Zhou M, Tang B (2019). Surface sensitive photoluminescence of carbon nanodots: coupling between the carbonyl group and π-electron system. J Phys Chem Lett.

[89] Wang S, Wu SH, Fang WL, Guo XF, Wang H (2019). Synthesis of non-doped and non-modified carbon dots with high quantum yield and crystallinity by one-pot hydrothermal method using a single carbon source and used for ClO- detection. Dyes And Pigments.

[90] Hu Y, Yang J, Jia L, Yu JS (2015). Ethanol in aqueous hydrogen peroxide solution: Hydrothermal synthesis of highly photoluminescent carbon dots as multifunctional nanosensors. Carbon.

[91] Ding H, Wei JS, Zhong N, Gao QY, Xiong HM (2017). Highly efficient red-emitting carbon dots with gram-scale yield for bioimaging. Langmuir.

[92] Liu H, Li Z, Sun Y, Geng X, Hu Y, Meng H (2018). Synthesis of luminescent carbon dots with ultrahigh quantum yield and inherent folate receptor-positive cancer cell targetability. Sci Rep.

[93] Rao L, Tang Y, Lu H, Yu S, Ding X, Xu K (2018). Highly photoluminescent and stable n-doped carbon dots as nanoprobes for Hg2+ detection. Nanomaterials (Basel).

[94] Anjana RR, Anjali Devi JS, Jayasree M, Aparna RS, Aswathy B, Praveen GL (2017). S,N-doped carbon dots as a fluorescent probe for bilirubin. Mikrochim Acta.

[95] Nozaki T, Kakuda T, Pottathara YB, Kawasaki H (2019). A nanocomposite of N-doped carbon dots with gold nanoparticles for visible light active photosensitisers. Photochem Photobiol Sci.

[96] Edison TN, Atchudan R, Sethuraman MG, Shim JJ, Lee YR (2016). Microwave assisted green synthesis of fluorescent N-doped carbon dots: Cytotoxicity and bio-imaging applications. J Photochem Photobiol B.

[97] Jiang K, Sun S, Zhang L, Wang Y, Cai C, Lin H Bright-yellow-emissive n-doped carbon dots: preparation, cellular imaging, and bifunctional sensing, ACS Appl Mater Interfaces. 2015; 7: 23231-23238.

[98] Sun X, Yang S, Guo M, Ma S, Zheng M, He J (2017). Reversible fluorescence probe based on n-doped carbon dots for the determination of mercury ion and glutathione in waters and living cells. Anal Sci.

[99] Wang B, Lin Y, Tan H, Luo M, Dai S, Lu H (2018). One-pot synthesis of N-doped carbon dots by pyrolyzing the gel composed of ethanolamine and 1-carboxyethyl-3-methylimidazolium chloride and their selective fluorescence sensing for Cr(vi) ions. Analyst.

[100] Zhang Y, Yuan R, He M, Hu G, Jiang J, Xu T (2017). Multicolour nitrogen-doped carbon dots: tunable photoluminescence and sandwich fluorescent glass-based light-emitting diodes. Nanoscale.

[101] Li Y, Bai G, Zeng S, Hao J (2019). Theranostic carbon dots with innovative nir-ii emission for *in vivo* renal-excreted optical imaging and photothermal therapy. ACS Appl Mater Interfaces.

[102] Bao L, Liu C, Zhang ZL, Pang DW (2015). Photoluminescence-tunable carbon nanodots: surface-state energy-gap tuning. Adv Mater.

[103] Yuan F, He P, Xi Z (2019). Highly efficient and stable white LEDs based on pure red narrow bandwidth emission triangular carbon quantum dots for wide-color gamut backlight displays. Nano Res.

[104] Liu Y, Gou H, Huang X, Zhang G, Xi K, Jia X (2020). Rational synthesis of highly efficient ultra-narrow red-emitting carbon quantum dots for NIR-II two-photon bioimaging. Nanoscale.

[105] Lin S, Lin C, He M (2017). Solvatochromism of bright carbon dots with tunable long-wavelength emission from green to red and their application as solid-state materials for warm WLEDs. Rsc Advances.

[106] Jia Q, Ge J, Liu W (2018). A Magnetofluorescent carbon dot assembly as an acidic H_2_O_2_ -driven oxygenerator to regulate tumor hypoxia for simultaneous bimodal imaging and enhanced photodynamic therapy. Adv Mater.

[107] Ding H, Wei JS, Zhang P, Zhou ZY, Gao QY, Xiong HM (2018). Solvent-controlled synthesis of highly luminescent carbon dots with a wide color gamut and narrowed emission peak widths. Small.

[108] Liang T, Liu E, Li M, Ushakova EV, Kershaw SV, Rogach AL (2021). Morphology control of luminescent carbon nanomaterials: from dots to rolls and belts. ACS Nano.

[109] Wang C, Jiang K, Wu Q, Wu J, Zhang C (2016). Green synthesis of red-emitting carbon nanodots as a novel "turn-on" nanothermometer in living cells. Chemistry.

[110] Hu H, Tian X, Gong Y, Ren G, Liang J (2019). N-doped carbon dots under Xenon lamp irradiation: Fluorescence red-shift and its potential mechanism. Spectrochim Acta A Mol Biomol Spectrosc.

[111] Geng X, Sun Y, Li Z, Yang R, Zhao Y, Guo Y (2019). Retrosynthesis of tunable fluorescent carbon dots for precise long-term mitochondrial tracking. Small.

[112] Zhi B, Gallagher MJ, Frank BP, Lyons TY, Qiu TA, Da J (2018). Investigation of phosphorous doping effects on polymeric carbon dots: Fluorescence, photostability, and environmental impact. Carbon.

[113] Long P, Feng Y, Cao CL, Yu H (2018). Junkai. Self-protective room-temperature phosphorescence of fluorine and nitrogen codoped carbon dots. Adv Funct Mater.

[114] Jiang L, Ding H, Lu S, Geng T, Xiao G, Zou B (2020). Photoactivated fluorescence enhancement in f,n-doped carbon dots with piezochromic behavior. Angew Chem Int Ed Engl.

[115] Pang LF, Wu H, Fu MJ, Guo XF, Wang H (2019). Red emissive boron and nitrogen co-doped "on-off-on" carbon dots for detecting and imaging of mercury(II) and biothiols. Mikrochim Acta.

[116] Das P, Ganguly S, Maity PP, Srivastava HK, Bose M, Dhara S (2019). Converting waste Allium sativum peel to nitrogen and sulphur co-doped photoluminescence carbon dots for solar conversion, cell labeling, and photobleaching diligences: A path from discarded waste to value-added products. J Photochem Photobiol B.

[117] Li J, Tang K, Yu J, Wang H, Tu M, Wang X (2019). Nitrogen and chlorine co-doped carbon dots as probe for sensing and imaging in biological samples. R Soc Open Sci.

[118] Tiwari A, Verma NC, Singh A, Nandi CK, Randhawa JK (2018). Carbon coated core-shell multifunctional fluorescent SPIONs. Nanoscale.

[119] Fu CC, Wu CY, Chien CC, Hsu TH, Ou SF, Chen ST (2020). Polyethylene glycol6000/carbon nanodots as fluorescent bioimaging agents. Nanomaterials (Basel).

[120] Mazrad ZAI, Kang EB, In I, Park SY (2018). Preparation of carbon dot-based ratiometric fluorescent probes for cellular imaging from Curcuma longa. Luminescence.

[121] Wang Y, Cao P, Li S, Zhang X, Hu J, Yang M (2018). Layer-by-layer assembled PEI-based vector with the upconversion luminescence marker for gene delivery. Biochem Biophys Res Commun.

[122] Liu C, Zhang P, Zhai X, Tian F, Li W, Yang J (2012). Nano-carrier for gene delivery and bioimaging based on carbon dots with PEI-passivation enhanced fluorescence. Biomaterials.

[123] Aspa B, Rdw B, Sppa B, Sts A, Gbkb C, Ds C (2020). Photophysical insights of highly transparent, flexible and re-emissive PVA @ WTR-CDs composite thin films: A next generation food packaging material for UV blocking applications. J Photochem Photobiol A Chem.

[124] Huang SW, Lin YF, Li YX, Hu CC, Chiu TC (2019). Synthesis of fluorescent carbon dots as selective and sensitive probes for cupric ions and cell imaging. Molecules.

[125] Liang P, Zheng Y, Zhang X, Wei H, Xu X, Yang X (2022). Carbon dots in hydroxy fluorides: achieving multicolor long-wavelength room-temperature phosphorescence and excellent stability via crystal confinement. Nano Lett.

[126] Chatterjee A, Ruturaj, Chakraborty MP, Nandi S, Purkayastha P (2022). Biocompatible and optically stable hydrophobic fluorescent carbon dots for isolation and imaging of lipid rafts in model membrane. Anal Bioanal Chem.

[127] Terracina A, Armano A, Meloni M, Panniello A, Minervini G, Madonia A (2022). Photobleaching and Recovery Kinetics of a Palette of Carbon Nanodots Probed by *In situ* Optical Spectroscopy. ACS Appl Mater Interfaces.

[128] Wu RS, Lin YS, Nain A, Unnikrishnan B, Lin YF, Yang CR (2022). Evaluation of chemotherapeutic response in living cells using subcellular Organelle-Selective amphipathic carbon dots. Biosens Bioelectron.

[129] Zhang Q, Wang R, Feng B, Zhong X, Ostrikov KK (2021). Photoluminescence mechanism of carbon dots: triggering high-color-purity red fluorescence emission through edge amino protonation. Nat Commun.

[130] Wang X, Zhang Y, Zhang M, Kong H, Wang S, Cheng J (2019). Novel carbon dots derived from puerariae lobatae radix and their anti-gout effects. Molecules.

[131] Wang M, Liu Y, Ren G, Wang W, Wu S, Shen J (2018). Bioinspired carbon quantum dots for sensitive fluorescent detection of vitamin B12 in cell system. Anal Chim Acta.

[132] Long R, Guo Y, Xie L, Shi S, Xu J, Tong C (2020). White pepper-derived ratiometric carbon dots for highly selective detection and imaging of coenzyme A. Food Chem.

[133] Pleskova SN, Mikheeva ER, Gornostaeva EE (2018). The interaction between human blood neutrophil granulocytes and quantum dots. Micron.

[134] Wang L, Zhang X, Yang K, Wang L, Lee CS (2020). Oxygen/nitrogen-related surface states controlled carbon nanodots with tunable full-color luminescence: Mechanism and bio-imaging. Carbon.

[135] Hong W, Liu Y, Li MH, Xing YX, Chen T, Fu YH (2018). *In vivo* toxicology of carbon dots by ^1^H NMR-based metabolomics. Toxicol Res (Camb).

[136] Li S, Guo Z, Zhang Y, Xue W, Liu Z (2015). Blood compatibility evaluations of fluorescent carbon dots. ACS Appl Mater Interfaces.

[137] Hu Y, Yang Z, Lu X, Guo J, Cheng R, Zhu L (2020). Facile synthesis of red dual-emissive carbon dots for ratiometric fluorescence sensing and cellular imaging. Nanoscale.

[138] Lu X, Zhang Z, Xia Q, Hou M, Yan C, Chen Z (2018). Glucose functionalized carbon quantum dot containing organic radical for optical/MR dual-modality bioimaging. Mater Sci Eng C Mater Biol Appl.

[139] Lu W, Jiao Y, Gao Y, Qiao J, Mozneb M, Shuang S (2018). Bright yellow fluorescent carbon dots as a multifunctional sensing platform for the label-free detection of fluoroquinolones and histidine. ACS Appl Mater Interfaces.

[140] Ding YY, Gong XJ, Liu Y, Lu WJ, Gao YF, Xian M (2018). Facile preparation of bright orange fluorescent carbon dots and the constructed biosensing platform for the detection of pH in living cells. Talanta.

[141] Bagheri Z, Ehtesabi H, Hallaji Z, Aminoroaya N, Tavana H, Behroodi E (2018). On-chip analysis of carbon dots effect on yeast replicative lifespan. Anal Chim Acta.

[142] Vasimalai N, Vilas-Boas V, Gallo J, Cerqueira MF, Menendez-Miranda M, Costa-Fernandez JM (2018). Green synthesis of fluorescent carbon dots from spices for *in vitro* imaging and tumour cell growth inhibition. Beilstein J Nanotechnol.

[143] Sayes CM, Liang F, Hudson JL, Mendez J, Guo W, Beach JM (2006). Functionalization density dependence of single-walled carbon nanotubes cytotoxicity *in vitro*. Toxicol Lett.

[144] Xiao A, Wang C, Chen J, Guo R, Yan Z, Chen J (2016). Carbon and metal quantum dots toxicity on the microalgae chlorella pyrenoidosa. Ecotoxicol Environ Saf.

[145] Pereira MM, Mouton L, Yepremian C, Coute A, Lo J, Marconcini JM (2014). Ecotoxicological effects of carbon nanotubes and cellulose nanofibers in Chlorella vulgaris. J Nanobiotechnology.

[146] Jiang Y, Zhang H, Wang Y, Chen M, Ye S, Hou Z (2013). Modulation of apoptotic pathways of macrophages by surface-functionalized multi-walled carbon nanotubes. PLoS One.

[147] Liu S, Tian J, Wang L, Zhang Y, Qin X, Luo Y (2012). Hydrothermal treatment of grass: a low-cost, green route to nitrogen-doped, carbon-rich, photoluminescent polymer nanodots as an effective fluorescent sensing platform for label-free detection of Cu(II) ions. Adv Mater.

[148] Wang J, Wang CF, Chen S (2012). Amphiphilic egg-derived carbon dots: rapid plasma fabrication, pyrolysis process, and multicolor printing patterns. Angew Chem Int Ed Engl.

[149] Zhu C, Zhai J, Dong S (2012). Bifunctional fluorescent carbon nanodots: green synthesis via soy milk and application as metal-free electrocatalysts for oxygen reduction. Chem Commun (Camb).

[150] Vadivel R, Thiyagarajan SK, Raji K, Suresh R, Sekar R, Ramamurthy PJASC (2016). Outright green synthesis of fluorescent carbon dots from eutrophic algal blooms for *in vitro* imaging. ACS Sustain Chem Eng.

[151] Wang Z, Han L, Hao W, Wang B, Zhao H, Tan M (2015). Fluorescent carbon dots from beer for breast cancer cell imaging and drug delivery. Analytical Methods.

[152] Xu J, Zhou Y, Cheng G, Dong M, Liu S, Huang C (2015). Carbon dots as a luminescence sensor for ultrasensitive detection of phosphate and their bioimaging properties. Luminescence.

[153] Jiang C, Wu H, Song X, Ma X, Wang J, Tan M (2014). Presence of photoluminescent carbon dots in Nescafe® original instant coffee: applications to bioimaging. Talanta.

[154] Xu ZQ, Yang LY, Fan XY, Jin JC, Liu Y (2014). Low temperature synthesis of highly stable phosphate functionalized two color carbon nanodots and their application in cell imaging. Carbon.

[155] Chaudhry N, Gupta PK, Eremin S, Solanki PR (2020). One-step green approach to synthesize highly fluorescent carbon quantum dots from banana juice for selective detection of copper ions. J Environ Chem Eng.

[156] Jahan S, Mansoor F, Naz S, Lei J, Kanwal S (2013). Oxidative synthesis of highly fluorescent boron/nitrogen co-doped carbon nanodots enabling detection of photosensitizer and carcinogenic dye. Anal Chem.

[157] Wu ZL, Zhang P, Gao MX, Liu CF, Wang W, Leng F (2013). One-pot hydrothermal synthesis of highly luminescent nitrogen-doped amphoteric carbon dots for bioimaging from Bombyx mori silk - natural proteins. J Mater Chem B.

[158] Lai CW, Hsiao YH, Peng YK, Chou PT (2012). Facile synthesis of highly emissive carbon dots from pyrolysis of glycerol; gram scale production of carbon dots/mSiO(2) for cell imaging and drug release. J Mater Chem B.

[159] Chen QL, Wang CF, Chen SJJoMS (2013). One-step synthesis of yellow-emitting carbogenic dots toward white light-emitting diodes. J Mater Sci Mater Med.

[160] Tabak M, Armon R, Neeman I (1999). Cinnamon extracts' inhibitory effect on Helicobacter pylori. J Ethnopharmacol.

[161] Geng BJ, Hu JY, Li P, Pan DY, Shen LX (2022). DNA binding graphene quantum dots inhibit dual topoisomerases for cancer. Carbon.

[162] Jiang BP, Zhou B, Shen XC, Yu YX, Ji SC, Wen CC (2015). Selective probing of gaseous ammonia using red-emitting carbon dots based on an interfacial response mechanism. Chemistry.

[163] Kasibabu BS, D'Souza S L, Jha S, Kailasa SK (2015). Imaging of bacterial and fungal cells using fluorescent carbon dots prepared from carica papaya juice. J Fluoresc.

[164] Ding H, Ji Y, Wei JS, Gao QY, Zhou ZY, Xiong HM (2017). Facile synthesis of red-emitting carbon dots from pulp-free lemon juice for bioimaging. J Mater Chem B.

[165] Qi HY, Huang DM, Jing J, Rano M, Jing T, Zhao M (2021). Simple and eco-friendly synthesis of crude orange-peel-derived carbon nanoparticles for detection of Fe3+ and ascorbic acid. Luminescence.

[166] Pal T, Mohiyuddin S, Packirisamy G (2018). Facile and green synthesis of multicolor fluorescence carbon dots from curcumin: *in vitro* and *in vivo* bioimaging and other applications. ACS Omega.

[167] Hu Y, Ji W, Sun J, Liu X, Zhou R, Yan J (2021). Simple and eco-friendly synthesis of crude orange-peel-derived carbon nanoparticles for detection of Fe3+ and ascorbic acid. Luminescence.

[168] Bayda S, Hadla M, Palazzolo S, Kumar V, Caligiuri I, Ambrosi E (2017). Bottom-up synthesis of carbon nanoparticles with higher doxorubicin efficacy. J Control Release.

[169] Zhang J, Yuan Y, Liang G, Yu SH (2015). Scale-up synthesis of fragrant nitrogen-doped carbon dots from bee pollens for bioimaging and catalysis. Adv Sci (Weinh).

[170] Alam AM, Park BY, Ghouri ZK, Park M, Kim HY (2015). Synthesis of carbon quantum dots from cabbage with down- and up-conversion photoluminescence properties: excellent imaging agent for biomedical applications. Green Chem.

[171] Bandi R, Gangapuram BR, Dadigala R, Eslavath R, Singh SS, Guttena V (2016). Facile and green synthesis of fluorescent carbon dots from onion waste and their potential applications as sensor and multicolour imaging agents. RSC Adv.

[172] Kumar A, Chowdhuri AR, Laha D, Mahto TK, Karmakar P, Sahu SK (2016). Green synthesis of carbon dots from Ocimum sanctum for effective fluorescent sensing of Pb2+ ions and live cell imaging. Sens Actuators B Chem.

[173] Bhamore JR, Jha S, Park TJ, Kailasa SK (2019). Green synthesis of multi-color emissive carbon dots from Manilkara zapota fruits for bioimaging of bacterial and fungal cells. J Photochem Photobiol B.

[174] Xia DL, Chen YP, Chen C, Wang YF, Li XD, He H (2015). Comparative study of biosafety, dna, and chromosome damage of different-materials-modified fe3o4 in rats. Appl Biochem Biotechnol.

[175] Bao X, Yuan Y, Chen J, Zhang B, Li D, Zhou D (2018). *In vivo* theranostics with near-infrared-emitting carbon dots-highly efficient photothermal therapy based on passive targeting after intravenous administration. Light Sci Appl.

[176] Du F, Zhang L, Zhang L, Zhang M, Gong A, Tan Y (2017). Engineered gadolinium-doped carbon dots for magnetic resonance imaging-guided radiotherapy of tumors. Biomaterials.

[177] Song Y, Wu Y, Wang H, Liu S, Song L, Li S (2019). Carbon quantum dots from roasted Atlantic salmon (Salmo salar L.): Formation, biodistribution and cytotoxicity. Food Chem.

[178] Kang YF, Li YH, Fang YW, Xu Y, Wei XM, Yin XB (2015). Carbon quantum dots for zebrafish fluorescence imaging. Sci Rep.

[179] Chen H, Wang GD, Tang W, Todd T, Zhen Z, Tsang C (2014). Gd-encapsulated carbonaceous dots with efficient renal clearance for magnetic resonance imaging. Adv Mater.

[180] Huang X, Zhang F, Zhu L, Choi KY, Guo N, Guo J (2013). Effect of injection routes on the biodistribution, clearance, and tumor uptake of carbon dots. ACS Nano.

[181] Singh V, Rawat KS, Mishra S, Baghel T, Fatima S, John AA (2018). Biocompatible fluorescent carbon quantum dots prepared from beetroot extract for *in vivo* live imaging in C. elegans and BALB/c mice. J Mater Chem B.

[182] Liu J, Li D, Zhang K, Yang M, Sun H, Yang B (2018). One-step hydrothermal synthesis of nitrogen-doped conjugated carbonized polymer dots with 31% efficient red emission for *in vivo* imaging. Small.

[183] Gao J, Chen K, Xie R, Xie J, Lee S, Cheng Z (2010). Ultrasmall near-infrared non-cadmium quantum dots for *in vivo* tumor imaging. Small.

[184] Li J, Rao J, Pu K (2018). Recent progress on semiconducting polymer nanoparticles for molecular imaging and cancer phototherapy. Biomaterials.

[185] Ma J, Kang K, Zhang Y, Yi Q, Gu Z (2018). Detachable polyzwitterion-coated ternary nanoparticles based on peptide dendritic carbon dots for efficient drug delivery in cancer therapy. ACS Appl Mater Interfaces.

[186] Yu Y, Song M, Chen C, Du Y, Li C, Han Y (2020). Bortezomib-encapsulated cus/carbon dot nanocomposites for enhanced photothermal therapy via stabilization of polyubiquitinated substrates in the proteasomal degradation pathway. ACS Nano.

[187] Kim SH, In I, Park SY (2017). pH-Responsive nir-absorbing fluorescent polydopamine with hyaluronic acid for dual targeting and synergistic effects of photothermal and chemotherapy. Biomacromolecules.

[188] Lesani P, Singh G, Viray CM, Ramaswamy Y, Zhu M, Kingshott P (2020). Two-photon dual-emissive carbon dot-based probe: deep-tissue imaging and ultrasensitive sensing of intracellular ferric ions. ACS Appl Mater Interfaces.

[189] Zhong H, Yu S, Li B, He K, Li D, Wang X (2021). Two-photon fluorescence and MR bio-imaging of endogenous H2O2 in the tumor microenvironment using a dual-mode nanoprobe. Chem Commun (Camb).

[190] Liu Y, Song Y, Zhang J, Yang Z, Peng X, Yan W (2021). Responsive carbonized polymer dots for optical super-resolution and fluorescence lifetime imaging of nucleic acids in living cells. ACS Appl Mater Interfaces.

[191] Ding F, Zhan Y, Lu X, Sun Y (2018). Recent advances in near-infrared II fluorophores for multifunctional biomedical imaging. Chem Sci.

[192] Zhang H, Wang G, Zhang Z, Lei JH, Liu TM, Xing G (2022). One step synthesis of efficient red emissive carbon dots and their bovine serum albumin composites with enhanced multi-photon fluorescence for *in vivo* bioimaging. Light Sci Appl.

[193] Zhao Y, Xie Y, Liu Y, Tang X, Cui S (2022). Comprehensive exploration of long-wave emission carbon dots for brain tumor visualization. J Mater Chem B.

[194] Liu Y, Lei JH, Wang G, Zhang Z, Wu J, Zhang B (2022). Toward strong near-infrared absorption/emission from carbon dots in aqueous media through solvothermal fusion of large conjugated perylene derivatives with post-surface engineering. Adv Sci (Weinh).

[195] Li H, Vaughan JC (2018). Switchable fluorophores for single-molecule localization microscopy. Chem Rev.

[196] Heilemann M, Margeat E, Kasper R, Sauer M, Tinnefeld P (2005). Carbocyanine dyes as efficient reversible single-molecule optical switch. J Am Chem Soc.

[197] Patterson GH, Lippincott-Schwartz J (2002). A photoactivatable GFP for selective photolabeling of proteins and cells. Science.

[198] Hoyer P, Staudt T, Engelhardt J, Hell SW (2011). Quantum dot blueing and blinking enables fluorescence nanoscopy. Nano Lett.

[199] Dempsey GT, Vaughan JC, Chen KH, Bates M, Zhuang X (2011). Evaluation of fluorophores for optimal performance in localization-based super-resolution imaging. Nat Methods.

[200] Shroff H, Galbraith CG, Galbraith JA, Betzig E (2008). Live-cell photoactivated localization microscopy of nanoscale adhesion dynamics. Nat Methods.

[201] Wang Y, Fruhwirth G, Cai E, Ng T, Selvin PR (2013). 3D super-resolution imaging with blinking quantum dots. Nano Lett.

[202] Hua XW, Bao YW, Zeng J, Wu FG (2019). Nucleolus-Targeted Red Emissive Carbon Dots with Polarity-Sensitive and Excitation-Independent Fluorescence Emission: High-Resolution Cell Imaging and *in vivo* Tracking. ACS Appl Mater Interfaces.

[203] Yu Y, Wang X, Jia X, Feng Z, Zhang L, Li H (2021). Aptamer probes labeled with lanthanide-doped carbon nanodots permit dual-modal fluorescence and mass cytometric imaging. Adv Sci (Weinh).

[204] Liang T, Liu E, Li M, Ushakova EV, Kershaw SV, Rogach AL (2021). Morphology control of luminescent carbon nanomaterials: from dots to rolls and belts. ACS Nano.

[205] Zhang Y, Song H, Wang L, Yu J, Wang B, Hu Y (2021). Solid-state red laser with a single longitudinal mode from carbon dots. Angew Chem Int Ed Engl.

[206] Karakoçak BB, Laradji A, Primeau T, Berezin MY, Li S, Ravi N (2021). Hyaluronan-conjugated carbon quantum dots for bioimaging use. ACS Appl Mater Interfaces.

[207] Li L, Zhang Q, Li J, Tian Y, Kang Y, Ren G (2021). Targeted delivery of doxorubicin using transferrin-conjugated carbon dots for cancer therapy. ACS Appl Bio Mater.

[208] Liu Z, Li B, Feng Y, Jia D, Li C, Zhou Y (2022). N-doped sp2 /sp3 carbon derived from carbon dots to boost the performance of ruthenium for efficient hydrogen evolution reaction. Small Methods.

[209] Lu SY, Wang J, Wang X, Yang W, Jin M, Xu L (2022). Janus-like bx c/c quantum sheets with z-scheme mechanism strengthen tumor photothermal-immunotherapy in nir-ii biowindow. Small Methods.

[210] Ye Y, Wang H, Liu H, Xiang Y, Liu L, Deng W (2022). Carbon dots-regulated pomegranate-like metal oxide composites: from growth mechanism to lithium storage. Small Methods.

[211] Wang X, Zhang Y, Li J, Liu G, Gao M, Ren S (2022). Platinum cluster/carbon quantum dots derived graphene heterostructured carbon nanofibers for efficient and durable solar-driven electrochemical hydrogen evolution. Small methods.

[212] Wu RS, Lin YS, Nain A, Unnikrishnan B, Lin YF, Yang CR (2022). Evaluation of chemotherapeutic response in living cells using subcellular organelle-selective amphipathic carbon dots. Biosens Bioelectron.

[213] Lu S, Sui L, Liu J, Zhu S, Chen A, Jin M (2017). Near-infrared photoluminescent polymer-carbon nanodots with two-photon fluorescence. Adv Mater.

[214] Li H, Yan X, Qiao S, Lu G, Su X (2018). Yellow-emissive carbon dot-based optical sensing platforms: cell imaging and analytical applications for biocatalytic reactions. ACS Appl Mater Interfaces.

[215] Liu J, Dong Y, Ma Y, Han Y, Ma S, Chen H (2018). One-step synthesis of red/green dual-emissive carbon dots for ratiometric sensitive ONOO- probing and cell imaging. Nanoscale.

[216] Krysmann MJ, Kelarakis A, Dallas P, Giannelis EP (2012). Formation mechanism of carbogenic nanoparticles with dual photoluminescence emission. J Am Chem Soc.

[217] Shamsipur M, Barati A, Taherpour AA, Jamshidi M (2018). Resolving the multiple emission centers in carbon dots: from fluorophore molecular states to aromatic domain states and carbon-core states. J Phys Chem Lett.

[218] Ko HY, Chang YW, Paramasivam G, Jeong MS, Cho S, Kim S (2013). *In vivo* imaging of tumour bearing near-infrared fluorescence-emitting carbon nanodots derived from tire soot. Chem Commun (Camb).

[219] Bao L, Liu C, Zhang ZL, Pang DW (2015). Photoluminescence-tunable carbon nanodots: surface-state energy-gap tuning. Adv Mater.

[220] Bai J, Xiao N, Wang Y, Li H, Qiu J (2021). Coal tar pitch derived nitrogen-doped carbon dots with adjustable particle size for photocatalytic hydrogen generation. Carbon.

[221] Tan X, Li Y, Li X, Zhou S, Fan L, Yang S (2015). Electrochemical synthesis of small-sized red fluorescent graphene quantum dots as a bioimaging platform. Chem Commun.

[222] Deng J, Lu Q, Mi N, Li H, Liu M, Xu M (2014). Electrochemical synthesis of carbon nanodots directly from alcohols. Chemistry.

[223] Yu H, Li X, Zeng X, Lu Y (2016). Preparation of carbon dots by non-focusing pulsed laser irradiation in toluene. Chem Commun (Camb).

[224] Nguyen V, Yan L, Si J, Xun HJ (2015). Femtosecond laser-induced size reduction of carbon nanodots in solution: Effect of laser fluence, spot size, and irradiation time. J appl phys.

[225] Wu Y, Liu Y, Yin J, Li H, Huang J (2019). Facile ultrasonic synthesized NH2-carbon quantum dots for ultrasensitive Co2+ ion detection and cell imaging. Talanta.

[226] Chen J, Wei JS, Zhang P, Niu XQ, Zhao W, Zhu ZY (2017). Red-emissive carbon dots for fingerprints detection by spray method: coffee ring effect and unquenched fluorescence in drying process. ACS Appl Mater Interfaces.

[227] Mutuyimana FP, Liu J, Na M, Nsanzamahoro S, Rao Z, Chen H (2018). Synthesis of orange-red emissive carbon dots for fluorometric enzymatic determination of glucose. Mikrochim Acta.

[228] Yang X, Cui F, Ren R, Sun J, Ji J, Pi F (2019). Red-emissive carbon dots for "switch-on" dual function sensing platform rapid detection of ferric ions and l-cysteine in living cells. ACS Omega.

[229] Li S, Jiang J, Yan Y, Wang P, Huang G, Kim NH (2018). Red, green, and blue fluorescent folate-receptor-targeting carbon dots for cervical cancer cellular and tissue imaging. Mater Sci Eng C Mater Biol Appl.

[230] Huang S, Yang E, Yao J, Liu Y, Xiao Q (2018). Red emission nitrogen, boron, sulfur co-doped carbon dots for "on-off-on" fluorescent mode detection of Ag+ ions and l-cysteine in complex biological fluids and living cells. Anal Chim Acta.

[231] Xue B, Yang Y, Sun Y, Fan J, Li X, Zhang Z (2019). Photoluminescent lignin hybridized carbon quantum dots composites for bioimaging applications. Int J Biol Macromol.

[232] Ye X, Xiang Y, Wang Q, Li Z, Liu Z (2019). A Red emissive two-photon fluorescence probe based on carbon dots for intracellular pH detection. Small.

[233] Li T, Xie L, Long R, Tong C, Guo Y, Tong X (2019). Cetyltrimethyl ammonium mediated enhancement of the red emission of carbon dots and an advanced method for fluorometric determination of iron(III). Mikrochim Acta.

[234] Ding H, Yu SB, Wei JS, Xiong HM (2016). Full-color light-emitting carbon dots with a surface-state-controlled luminescence mechanism. ACS Nano.

[235] Zhang T, Zhao F, Li L, Qi B, Zhu D, Lu J (2018). Tricolor white-light-emitting carbon dots with multiple-cores@shell structure for wled application. ACS Appl Mater Interfaces.

[236] Li S, Su W, Wu H, Yuan T, Yuan C, Liu J (2020). Targeted tumour theranostics in mice via carbon quantum dots structurally mimicking large amino acids. Nat Biomed Eng.

[237] Xu J, Li J, Wang C, Zhao W (2020). Preparation and application of solvent-modulated self-doped N-S multicolour fluorescence carbon quantum dots. Luminescence.

[238] Hu Y, Gao Z, Luo J (2021). Fluorescence detection of malachite green in fish tissue using red emissive Se,N,Cl-doped carbon dots. Food Chem.

[239] Wu J, Wang W, Wang Z (2020). Porphin-based carbon dots for "turn off-on" phosphate sensing and cell imaging. Nanomaterials (Basel).

[240] Wang Z, Yuan F, Li X, Li Y, Zhong H, Fan L (2017). 53% efficient red emissive carbon quantum dots for high color rendering and stable warm white-light-emitting diodes. Adv Mater.

[241] Yuan B, Guan S, Sun X, Li X, Zeng H (2018). Highly efficient carbon dots with reversibly switchable green-red emissions for trichromatic white light-emitting diodes. ACS Appl Mater Interfaces.

[242] Su W, Guo R, Yuan F, Li Y, Fan L (2020). Red-emissive carbon quantum dots for nuclear drug delivery in cancer stem cells. J Phys Chem Lett.

[243] Tan J, Yi Z, Ye Y, Ren X, Li Q (2020). Achieving red room temperature afterglow carbon dots in composite matrices through chromophore conjugation degree controlling. J Lumin.

[244] Pan L, Sun S, Zhang L, Jiang K, Lin H (2016). Near-infrared emissive carbon dots for two-photon fluorescence bioimaging. Nanoscale.

[245] Yuan F, Wang Z, Li X, Li Y, Tan Z, Fan L (2017). Bright multicolor bandgap fluorescent carbon quantum dots for electroluminescent light-emitting diodes. Adv Mater.

[246] Kumar A, Chowdhuri AR, Sahu SK (2018). Green synthesis of carbon dots from Ocimum sanctum for effective fluorescent sensing of Pb2+ ions and live cell imaging. Sensor actuat b-chem.

[247] Tian Z, Zhang XT, Li D, Zhou D, Jing PT, Shen DZ (2017). Full-color inorganic carbon dot phosphors for white-light-emitting diodes. Adv opt mater.

[248] Wang L, Li W, Yin L, Liu Y, Guo H, Lai J (2020). Full-color fluorescent carbon quantum dots. Sci Adv.

[249] Sciortino A, Marino E, Dam B, Schall P, Cannas M, Messina F (2016). Solvatochromism unravels the emission mechanism of carbon nanodots. J Phys Chem Lett.

[250] Nguyen HA, Srivastava I, Pan D, Gruebele M (2020). Unraveling the fluorescence mechanism of carbon dots with sub-single-particle resolution. ACS Nano.

[251] Srivastava I, Misra SK, Tripathi I, Duval AS, Pan D (2018). Synthesis of chiral carbo-nano-tweezers for enantiospecific recognition and DNA duplex winding in cancer cells. Adv biosyst.

[252] Jiang K, Feng X, Gao X, Wang Y, Cai C, Li Z (2019). Preparation of multicolor photoluminescent carbon dots by tuning surface states. Nanomaterials (Basel).

[253] Miao X, Qu D, Yang D, Nie B, Zhao Y, Fan H (2018). Synthesis of carbon dots with multiple color emission by controlled graphitization and surface functionalization. Adv Mater.

[254] Kundelev EV, Tepliakov NV, Leonov MY, Maslov VG, Baranov AV, Fedorov AV (2019). Amino functionalization of carbon dots leads to red emission enhancement. J Phys Chem Lett.

[255] Shao M, Yu Q, Jing N, Cheng Y, Wang D, Wang YD (2019). Continuous synthesis of carbon dots with full spectrum fluorescence and the mechanism of their multiple color emission. Lab Chip.

[256] Do S, Kwon W, Kim YH, Kang SR, Lee T, Lee TW (2016). N,S-induced electronic states of carbon nanodots toward white electroluminescence. Adv opt mater.

[257] Qu D, Sun ZC, Zheng M, Li J, Zhang YQ, Zhang GQ (2015). Three colors emission from s,n co-doped graphene quantum dots for visible light h-2 production and bioimaging. Adv opt mater.

[258] Zhao X, Dong L, Ming Y, Wang M, Lu Z, Xu Y (2019). A magnetofluorescent boron-doped carbon dots as a metal-free bimodal probe. Talanta.

[259] Choi Y, Kang B, Lee J, Kim S, Kim GT, Kang H (2016). Integrative approach toward uncovering the origin of photoluminescence in dual heteroatom-doped carbon nanodots. Chem Mater.

[260] Ding HZ, Xu JH, Jiang L, Dong C, Meng Q, Rehman, SU (2021). Fluorine-defects induced solid-state red emission of carbon dots with an excellent thermosensitivity. Chin Chem Lett.

[261] Jiang L, Ding H, Xu M, Hu X, Li S, Zhang M (2020). UV-Vis-NIR full-range responsive carbon dots with large multiphoton absorption cross sections and deep-red fluorescence at nucleoli and *in vivo*. Small.

[262] Zuo G, Xie A, Li J, Su T, Pan X, Dong W (2017). A global method for handling fluorescence spectra at high concentration derived from the competition between emission and absorption of colloidal CdTe quantum dots. J Phys Chem C.

[263] Luo TY, He X, Zhang J, Chen P, Liu YH, Wang HJ (2018). Photoluminescent F-doped carbon dots prepared by ring-opening reaction for gene delivery and cell imaging. RSC Adv.

[264] Zuo G, Xie A, Pan X, Su T, Li J, Dong W (2018). Fluorine-doped cationic carbon dots for efficient gene delivery. ACS Appl Nano Mater.

[265] Yang W, Zhang H, Lai J, Peng X, Hu Y, Gu W (2017). Excitation wavelength independent visible color emission of carbon dots. Nanoscale.

[266] Wang H, Sun C, Chen X, Zhang Y, Colvin VL, Rice Q (2017). Excitation wavelength independent visible color emission of carbon dots. Nanoscale.

[267] Jing P, Han D, Li D, Zhou D, Shen D, Xiao G (2019). Surface related intrinsic luminescence from carbon nanodots: solvent dependent piezochromism. Nanoscale Horiz.

[268] Meng X, Chang Q, Xue C, Yang J, Hu S (2017). Full-colour carbon dots: from energy-efficient synthesis to concentration-dependent photoluminescence properties. Chem Commun (Camb).

[269] Wang Y, Kalytchuk S, Zhang Y, Shi H, Kershaw SV, Rogach AL (2014). Thickness-dependent full-color emission tunability in a flexible carbon dot ionogel. J Phys Chem Lett.

[270] Sun C, Zhang Y, Kalytchuk S, Wang Y, Zhang X (2015). Down-conversion monochromatic light-emitting diodes with the color determined by the active layer thickness and concentration of carbon dots. J Mater Chem C Mater.

[271] Ju YJ, Li N, Liu SG, Liang JY, Gao X, Fan YZ (2019). Proton-controlled synthesis of red-emitting carbon dots and application for hematin detection in human erythrocytes. Anal Bioanal Chem.

[272] Wu S, Li W, Sun Y, Zhang X, Zhuang J, Hu H (2019). Synthesis of dual-emissive carbon dots with a unique solvatochromism phenomenon. J Colloid Interface Sci.

[273] Duan J, Yu J, Feng S, Su L (2016). A rapid microwave synthesis of nitrogen-sulfur co-doped carbon nanodots as highly sensitive and selective fluorescence probes for ascorbic acid. Talanta.

[274] Lu H, Yu C, Zhang Y, Xu S (2019). Efficient core shell structured dual response ratiometric fluorescence probe for determination of H2O2 and glucose via etching of silver nanoprisms. Anal Chim Acta.

[275] Wu JX, Yan B (2017). Visible detection of copper ions using a fluorescent probe based on red carbon dots and zirconium metal-organic frameworks. Dalton Trans.

[276] Yuan F, Ding L, Li Y, Li X, Fan L, Zhou S (2015). Multicolor fluorescent graphene quantum dots colorimetrically responsive to all-pH and a wide temperature range. Nanoscale.

[277] Peng Z, Miyanji EH, Zhou Y, Pardo J, Hettiarachchi SD, Li S (2017). Carbon dots: promising biomaterials for bone-specific imaging and drug delivery. Nanoscale.

[278] Li S, Skromne I, Peng Z, Dallman J, Al-Youbi AO, Bashammakh AS (2016). "Dark" carbon dots specifically "light-up" calcified zebrafish bones. J Mater Chem B.

[279] Cao L, Yang ST, Wang X, Luo PG, Liu JH, Sahu S (2012). Competitive performance of carbon "quantum" dots in optical bioimaging. Theranostics.

[280] Pardo J, Peng Z, Leblanc RM (2018). Cancer targeting and drug delivery using carbon-based quantum dots and nanotubes. Molecules.

[281] Qin H, Sun Y, Geng X, Zhao K, Meng H, Yang R (2020). A wash-free lysosome targeting carbon dots for ultrafast imaging and monitoring cell apoptosis status. Anal Chim Acta.

[282] Li S, Peng Z, Dallman J, Baker J, Othman AM, Blackwelder PL (2016). Crossing the blood-brain-barrier with transferrin conjugated carbon dots: A zebrafish model study. Colloids Surf B Biointerfaces.

[283] Li S, Amat D, Peng Z, Vanni S, Raskin S, De Angulo G (2016). Transferrin conjugated nontoxic carbon dots for doxorubicin delivery to target pediatric brain tumor cells. Nanoscale.

[284] Mintz KJ, Mercado G, Zhou Y, Ji Y, Hettiarachchi SD, Liyanage PY (2019). Tryptophan carbon dots and their ability to cross the blood-brain barrier. Colloids Surf B Biointerfaces.

[286] Yang X, Wang Y, Shen X, Su C, Yang J, Piao M (2017). One-step synthesis of photoluminescent carbon dots with excitation-independent emission for selective bioimaging and gene delivery. J Colloid Interface Sci.

[287] Song Y, Shi W, Chen W, Li X, Ma H (2012). Fluorescent carbon nanodots conjugated with folic acid for distinguishing folate-receptor-positive cancer cells from normal cells. J Mater Chem C.

[288] Liu Q, Xu S, Niu C, Li M, He D, Lu Z (2015). Distinguish cancer cells based on targeting turn-on fluorescence imaging by folate functionalized green emitting carbon dots. Biosens Bioelectron.

[289] Aiyer S, Prasad R, Kumar M, Nirvikar K, Jain B, Kushwaha OS (2016). Fluorescent carbon nanodots for targeted *in vitro* cancer cell imaging. Appl Mater Today.

[290] Li W, Liu Q, Zhang P, Liu L (2016). Zwitterionic nanogels crosslinked by fluorescent carbon dots for targeted drug delivery and simultaneous bioimaging. Acta Biomater.

[291] Zhang J, Zhao X, Xian M, Dong C, Shuang S (2018). Folic acid-conjugated green luminescent carbon dots as a nanoprobe for identifying folate receptor-positive cancer cells. Talanta.

[292] Mahani M, Mousapour Z, Divsar F, Nomani A, Ju H (2019). A carbon dot and molecular beacon based fluorometric sensor for the cancer marker microRNA-21. Mikrochim Acta.

[293] Jiang X, Zong S, Chen C, Zhang Y, Wang Z, Cui Y (2018). Gold-carbon dots for the intracellular imaging of cancer-derived exosomes. Nanotechnology.

[294] Xu LL, Zhang W, Shang L, Ma RN, Jia LP, Jia WL (2018). Perylenetetracarboxylic acid and carbon quantum dots assembled synergistic electrochemiluminescence nanomaterial for ultra-sensitive carcinoembryonic antigen detection. Biosens Bioelectron.

[295] Alarfaj NA, El-Tohamy MF, Oraby HF (2018). CA 19-9 Pancreatic tumor marker fluorescence immunosensing detection via immobilized carbon quantum dots conjugated gold nanocomposite. Int J Mol Sci.

[296] Mohammadi S, Salimi A, Hamd-Ghadareh S, Fathi F, Soleimani F (2018). A FRET immunosensor for sensitive detection of CA 15-3 tumor marker in human serum sample and breast cancer cells using antibody functionalized luminescent carbon-dots and AuNPs-dendrimer aptamer as donor-acceptor pair. Anal Biochem.

[297] Hamd-Ghadareh S, Salimi A, Parsa S, Fathi F (2018). Simultaneous biosensing of CA125 and CA15-3 tumor markers and imaging of OVCAR-3 and MCF-7 cells lines via bi-color FRET phenomenon using dual blue-green luminescent carbon dots with single excitation wavelength. Int J Biol Macromol.

[298] Hamd-Ghadareh S, Salimi A, Fathi F, Bahrami S (2017). An amplified comparative fluorescence resonance energy transfer immunosensing of CA125 tumor marker and ovarian cancer cells using green and economic carbon dots for bio-applications in labeling, imaging and sensing. Biosens Bioelectron.

[299] Li CF, Yan ZK, Chen LB, Jin JP, Li DD (2017). Desmin detection by facile prepared carbon quantum dots for early screening of colorectal cancer. Medicine (Baltimore).

[300] Gu C, Guo C, Li Z, Wang M, Zhou N, He L (2019). Bimetallic ZrHf-based metal-organic framework embedded with carbon dots: Ultra-sensitive platform for early diagnosis of HER2 and HER2-overexpressed living cancer cells. Biosens Bioelectron.

[301] Sun C, Pan L, Zhang L, Huang J, Yao D, Wang CZ (2019). A biomimetic fluorescent nanosensor based on imprinted polymers modified with carbon dots for sensitive detection of alpha-fetoprotein in clinical samples. Analyst.

[302] Gao G, Jiang YW, Yang J, Wu FG (2017). Mitochondria-targetable carbon quantum dots for differentiating cancerous cells from normal cells. Nanoscale.

[303] He X, Luo Q, Zhang J, Chen P, Wang HJ, Luo K (2019). Gadolinium-doped carbon dots as nano-theranostic agents for MR/FL diagnosis and gene delivery. Nanoscale.

[304] Lin B, Yu Y, Liu F, Cao Y, Guo M (2017). Tunable and nontoxic fluorescent probes based on carbon dots for imaging of indole propionic acid receptor in plant tissues *in situ*. J Fluoresc.

[305] Wang H, Mu Q, Wang K, Revia RA, Yen C, Gu X (2019). Nitrogen and boron dual-doped graphene quantum dots for near-infrared second window imaging and photothermal therapy. Appl Mater Today.

[306] Ren W, Chen S, Liao Y, Li S, Ge J, Tao F (2019). Near-infrared fluorescent carbon dots encapsulated liposomes as multifunctional nano-carrier and tracer of the anticancer agent cinobufagin *in vivo* and *in vitro*. Colloids Surf B Biointerfaces.

[307] Zhou N, Hao Z, Zhao X, Maharjan S, Zhu S, Song Y (2015). A novel fluorescent retrograde neural tracer: cholera toxin B conjugated carbon dots. Nanoscale.

[308] Anand A, Manavalan G, Mandal RP, Chang HT, Chiou YR, Huang CC (2019). Carbon dots for bacterial detection and antibacterial applications-a minireview. Curr Pharm Des.

[309] Kong T, Hao L, Wei Y, Cai X, Zhu B (2018). Doxorubicin conjugated carbon dots as a drug delivery system for human breast cancer therapy. Cell Prolif.

[310] Hua XW, Bao YW, Wu FG (2018). Fluorescent carbon quantum dots with intrinsic nucleolus-targeting capability for nucleolus imaging and enhanced cytosolic and nuclear drug delivery. ACS Appl Mater Interfaces.

[311] Kumari S, Solanki A, Mandal S, Subramanyam D, Das P (2018). Creation of linear carbon dot array with improved optical properties through controlled covalent conjugation with DNA. Bioconjug Chem.

[312] Song Y, Li X, Cong S, Zhao H, Tan M (2019). Nuclear-targeted of TAT peptide-conjugated carbon dots for both one-and two-photon fluorescence imaging. Colloids Surf B Biointerfaces.

[313] Shirata C, Kaneko J, Inagaki Y, Kokudo T, Sato M, Kiritani S (2017). Near-infrared photothermal/photodynamic therapy with indocyanine green induces apoptosis of hepatocellular carcinoma cells through oxidative stress. Sci Rep.

[314] Li X, Peng XH, Zheng BD, Tang J, Zhao Y, Zheng BY (2018). New application of phthalocyanine molecules: from photodynamic therapy to photothermal therapy by means of structural regulation rather than formation of aggregates. Chem Sci.

[315] Cai Y, Liang P, Tang Q, Si W, Chen P, Zhang Q (2017). Diketopyrrolopyrrole-based photosensitizers conjugated with chemotherapeutic agents for multimodal tumor therapy. ACS Appl Mater Interfaces.

[316] Harmatys KM, Battles PM, Peck EM, Spence GT, Roland FM, Smith BD (2017). Selective photothermal inactivation of cells labeled with near-infrared croconaine dye. Chem Commun (Camb).

[317] Ding K, Zhang Y, Si W, Zhong X, Cai Y, Zou J (2018). Zinc(II) metalated porphyrins as photothermogenic photosensitizers for cancer photodynamic/photothermal synergistic therapy. ACS Appl Mater Interfaces.

[318] Lal S, Clare SE, Halas NJ (2008). Nanoshell-enabled photothermal cancer therapy: impending clinical impact. Acc Chem Res.

[319] Xiao JW, Fan SX, Wang F, Sun LD, Zheng XY, Yan CH (2014). Porous Pd nanoparticles with high photothermal conversion efficiency for efficient ablation of cancer cells. Nanoscale.

[320] Li N, Sun Q, Yu Z, Gao X, Pan W, Wan X (2018). Nuclear-targeted photothermal therapy prevents cancer recurrence with near-infrared triggered copper sulfide nanoparticles. ACS Nano.

[321] Yang D, Yang GX, Yang PP, Lv RC (2017). Assembly of au plasmonic photothermal agent and iron oxide nanoparticles on ultrathin black phosphorus for targeted photothermal and photodynamic cancer therapy. Adv Funct Mater.

[322] Liu H, Li C, Qian Y, Hu L, Fang J, Tong W (2020). Magnetic-induced graphene quantum dots for imaging-guided photothermal therapy in the second near-infrared window. Biomaterials.

[323] Das RK, Panda S, Bhol CS, Bhutia SK, Mohapatra S (2019). N-doped carbon quantum dot (ncqd)-deposited carbon capsules for synergistic fluorescence imaging and photothermal therapy of oral cancer. Langmuir.

[324] Weng Y, Guan S, Wang L, Qu X, Zhou S (2019). Hollow carbon nanospheres derived from biomass by-product okara for imaging-guided photothermal therapy of cancers. J Mater Chem B.

[325] Ge J, Jia Q, Liu W, Guo L, Liu Q, Lan M (2015). Red-emissive carbon dots for fluorescent, photoacoustic, and thermal theranostics in living mice. Adv Mater.

[326] Zheng M, Li Y, Liu S, Wang W, Xie Z, Jing X (2016). One-pot to synthesize multifunctional carbon dots for near infrared fluorescence imaging and photothermal cancer therapy. ACS Appl Mater Interfaces.

[327] Lan M, Zhao S, Zhang Z, Li Y, Liang G, Niu G (2017). Two-photon-excited near-infrared emissive carbon dots as multifunctional agents for fluorescence imaging and photothermal therapy. Nano Res.

[328] Geng B, Qin H, Zheng F, Shen W, Li P, Wu K (2019). Carbon dot-sensitized MoS2 nanosheet heterojunctions as highly efficient NIR photothermal agents for complete tumor ablation at an ultralow laser exposure. Nanoscale.

[329] Arosio D, Casagrande C, Manzoni L (2012). Integrin-mediated drug delivery in cancer and cardiovascular diseases with peptide-functionalized nanoparticles. Curr Med Chem.

[330] Liu Z, Chen W, Li Y, Xu Q (2016). Integrin αvβ3-targeted c-dot nanocomposites as multifunctional agents for cell targeting and photoacoustic imaging of superficial malignant tumors. Anal Chem.

[331] Xiong XB, Huang Y, Lu WL, Zhang X, Zhang H, Nagai T (2005). Enhanced intracellular delivery and improved antitumor efficacy of doxorubicin by sterically stabilized liposomes modified with a synthetic RGD mimetic. J Control Release.

[332] Wang H, Wang K, Tian B, Revia R, Mu Q, Jeon M (2016). Preloading of hydrophobic anticancer drug into multifunctional nanocarrier for multimodal imaging, NIR-responsive drug release, and synergistic therapy. Small.

[333] Lu H, Zhao Q, Wang X, Mao Y, Chen C, Gao Y (2020). Multi-stimuli responsive mesoporous silica-coated carbon nanoparticles for chemo-photothermal therapy of tumor. Colloids Surf B Biointerfaces.

[334] Liu H, Lv X, Qian J, Li H, Qian Y, Wang X (2020). Graphitic carbon nitride quantum dots embedded in carbon nanosheets for near-infrared imaging-guided combined photo-chemotherapy. ACS Nano.

[335] He H, Zheng X, Liu S, Zheng M, Xie Z, Wang Y (2018). Diketopyrrolopyrrole-based carbon dots for photodynamic therapy. Nanoscale.

[336] Wang J, Xu M, Wang D, Li Z, Primo FL, Tedesco AC (2019). Copper-doped carbon dots for optical bioimaging and photodynamic therapy. Inorg Chem.

[337] Celli JP, Spring BQ, Rizvi I, Evans CL, Samkoe KS, Verma S (2010). Imaging and photodynamic therapy: mechanisms, monitoring, and optimization. Chem Rev.

[338] Cheng L, Kamkaew A, Sun H, Jiang D, Valdovinos HF, Gong H (2016). Dual-modality positron emission tomography/optical image-guided photodynamic cancer therapy with chlorin e6-containing nanomicelles. ACS Nano.

[339] Yang D, Yang G, Sun Q, Gai S, He F, Dai Y (2018). Carbon-dot-decorated TIO2 nanotubes toward photodynamic therapy based on water-splitting mechanism. Adv Healthc Mater.

[340] Zheng DW, Li B, Li CX, Fan JX, Lei Q, Li C (2016). Carbon-dot-decorated carbon nitride nanoparticles for enhanced photodynamic therapy against hypoxic tumor via water splitting. ACS Nano.

[341] Irmania N, Dehvari K, Gedda G, Tseng PJ, Chang JY (2020). Manganese-doped green tea-derived carbon quantum dots as a targeted dual imaging and photodynamic therapy platform. J Biomed Mater Res B Appl Biomater.

[342] Li Y, Zheng X, Zhang X, Liu S, Pei Q, Zheng M (2017). Porphyrin-based carbon dots for photodynamic therapy of hepatoma. Adv Healthc Mater.

[343] Arcudi F, Dordevic L, Prato M (2016). Synthesis, separation, and characterization of small and highly fluorescent nitrogen-doped carbon nanodots. Angew Chem Int Ed Engl.

[344] Zhang M, Wang W, Cui Y, Zhou N, Shen J (2018). Near-infrared light-mediated photodynamic/photothermal therapy nanoplatform by the assembly of Fe3O4 carbon dots with graphitic black phosphorus quantum dots. Int J Nanomedicine.

[345] Jia Q, Ge J, Liu W, Liu S, Niu G, Guo L (2016). Gold nanorod@silica-carbon dots as multifunctional phototheranostics for fluorescence and photoacoustic imaging-guided synergistic photodynamic/photothermal therapy. Nanoscale.

[346] Chen D, Tang Q, Zou J, Yang X, Huang W, Zhang Q (2018). PH-responsive peg-doxorubicin-encapsulated aza-bodipy nanotheranostic agent for imaging-guided synergistic cancer therapy. Adv Healthc Mater.

[347] Sun S, Chen J, Jiang K, Tang Z, Wang Y, Li Z (2019). Ce6-modified carbon dots for multimodal-imaging-guided and single-nir-laser-triggered photothermal/photodynamic synergistic cancer therapy by reduced irradiation power. ACS Appl Mater Interfaces.

[348] Zeng Q, Shao D, He X, Ren Z, Ji W, Shan C (2016). Carbon dots as a trackable drug delivery carrier for localized cancer therapy *in vivo*. J Mater Chem B.

[349] Wen Y, Xu M, Liu X, Jin X, Kang J, Xu D (2019). Magnetofluorescent nanohybrid comprising polyglycerol grafted carbon dots and iron oxides: Colloidal synthesis and applications in cellular imaging and magnetically enhanced drug delivery. Colloids Surf B Biointerfaces.

[350] Gao N, Yang W, Nie H, Gong Y, Jing J, Gao L (2017). Turn-on theranostic fluorescent nanoprobe by electrostatic self-assembly of carbon dots with doxorubicin for targeted cancer cell imaging, *in vivo* hyaluronidase analysis, and targeted drug delivery. Biosens Bioelectron.

[351] Jiang Q, Liu L, Li Q, Cao Y, Chen D, Du Q (2021). NIR-laser-triggered gadolinium-doped carbon dots for magnetic resonance imaging, drug delivery and combined photothermal chemotherapy for triple negative breast cancer. J Nanobiotechnology.

[352] Wang H, Mukherjee S, Yi J, Banerjee P, Chen Q, Zhou S (2017). Biocompatible chitosan-carbon dot hybrid nanogels for NIR-imaging-guided synergistic photothermal-chemo therapy. ACS Appl Mater Interfaces.

[353] Liu Z, Chen W, Li Y, Xu Q (2016). Integrin αvβ3-targeted c-dot nanocomposites as multifunctional agents for cell targeting and photoacoustic imaging of superficial malignant tumors. Anal Chem.

[354] Feng T, Ai X, An G, Yang P, Zhao Y (2016). Charge-convertible carbon dots for imaging-guided drug delivery with enhanced *in vivo* cancer therapeutic efficiency. ACS Nano.

[355] Feng T, Ai X, Ong H, Zhao Y (2016). Dual-responsive carbon dots for tumor extracellular microenvironment triggered targeting and enhanced anticancer drug delivery. ACS Appl Mater Interfaces.

[356] Zhang Y, Li S, Ma XT, He XW, Li WY, Zhang YK (2020). Carbon dots-embedded epitope imprinted polymer for targeted fluorescence imaging of cervical cancer via recognition of epidermal growth factor receptor. Mikrochim Acta.

[357] Loh XJ, Lee TC, Dou Q, Deen GR (2016). Utilising inorganic nanocarriers for gene delivery. Biomater Sci.

[358] Mohajeri M, Behnam B, Sahebkar A (2018). Biomedical applications of carbon nanomaterials: Drug and gene delivery potentials. J Cell Physiol.

[359] Mohammadinejad R, Karimi S, Iravani S, Varma RS (2016). Plant-derived nanostructures: types and applications. Green Chem.

[360] He X, Chen P, Zhang J, Luo TY, Wang HJ, Liu YH (2019). Cationic polymer-derived carbon dots for enhanced gene delivery and cell imaging. Biomater Sci.

[361] Lin G, Zhang H, Huang L (2015). Smart polymeric nanoparticles for cancer gene delivery. Mol Pharm.

[362] Hasanzadeh A, Radmanesh F, Kiani J, Bayandori M, Fatahi Y, Aref AR (2019). Photoluminescent functionalized carbon dots for CRISPR delivery: synthesis, optimization and cellular investigation. Nanotechnology.

[363] Jaleel JA, Ashraf SM, Rathinasamy K, Pramod K (2019). Carbon dot festooned and surface passivated graphene-reinforced chitosan construct for tumor-targeted delivery of TNF-α gene. Int J Biol Macromol.

[364] Jing N, Tian M, Wang YT, Zhang Y (2019). Nitrogen-doped carbon dots synthesized from acrylic acid and ethylenediamine for simple and selective determination of cobalt ions in aqueous media. J Lumin.

[365] Ghosh S, Ghosal K, Mohammad SA, Sarkar K (2019). Dendrimer functionalized carbon quantum dot for selective detection of breast cancer and gene therapy. Chem Eng J.

[366] Wang HJ, He X, Luo TY, Zhang J, Liu YH, Yu XQ (2017). Amphiphilic carbon dots as versatile vectors for nucleic acid and drug delivery. Nanoscale.

[367] Chen P, He X, Tian XL, Zhang J, Yu XQ (2021). One-step fabrication of functional carbon dots with long wavelength emission for gene delivery and bio-imaging. J Mater Chem B.

[368] Cao X, Wang J, Deng W, Chen J, Wang Y, Zhou J (2018). Photoluminescent cationic carbon dots as efficient non-viral delivery of plasmid SOX9 and chondrogenesis of fibroblasts. Sci Rep.

[369] Zhao A, Chen Z, Zhao C, Gao N, Ren J, Qu X (2015). Recent advances in bioapplications of C-dots. Carbon.

[370] Chen F, Qian K, Ailing F (2017). Arginine-modified carbon dots probe for live cell imaging and sensing by increasing cellular uptake efficiency. Mat Sci Eng C-Mater.

[371] Geng BJ, Hu JY, Li Y, Feng SN, Pan DY (2022). Near-infrared phosphorescent carbon dots for sonodynamic precision tumor therapy. Nat Commun.

